# OpiumPlex is a novel microsatellite system for profiling opium poppy (*Papaver somniferum* L.)

**DOI:** 10.1038/s41598-021-91962-1

**Published:** 2021-06-17

**Authors:** Jakub Vašek, Daniela Čílová, Martina Melounová, Pavel Svoboda, Kamila Zdeňková, Eliška Čermáková, Jaroslava Ovesná

**Affiliations:** 1 Department of Genetics and Breeding, FAFNR, Czech University of Life Sciences Prague, Prague, Czech Republic; 2 Division of Crop Genetics and Breeding, Crop Research Institute, Prague, Czech Republic; 3 Department of Biochemistry and Microbiology, University of Chemistry and Technology Prague, Prague, Czech Republic

**Keywords:** Genetics, Agricultural genetics, Genetic markers, Plant genetics, Plant sciences, Plant genetics

## Abstract

Opium poppy (*Papaver somniferum* L.) is a versatile plant exploited by the pharmaceutical and food industries. Unfortunately, it is also infamously known as a source of highly addictive narcotics, primarily heroin. Drug abuse has devastating consequences for users and also has many direct or indirect negative impacts on human society as a whole. Therefore, developing a molecular genetic tool for the individualization of opium poppy, raw opium or heroin samples could help in the fight against the drug trade by retrieving more information about the source of narcotics and linking isolated criminal cases. Bioinformatic analysis provided insight into the distribution, density and other characteristics of roughly 150 thousand microsatellite loci within the poppy genome and indicated underrepresentation of microsatellites with the desired attributes. Despite this fact, 27 polymorphic STR markers, divided into three multiplexed assays, were developed in this work. Internal validation confirmed species-specific amplification, showed that the optimal amount of DNA is within the range of 0.625–1.25 ng per reaction, and indicate relatively well balanced assays according to the metrics used. Moreover, the stutter ratio (mean + 3 SD 2.28–15.59%) and allele-specific stutters were described. The analysis of 187 individual samples led to the identification of 158 alleles in total, with a mean of 5.85 alleles and a range of 3–14 alleles per locus. Most of the alleles (151) were sequenced by the Sanger method, which enabled us to propose standardized nomenclature and create three allelic ladders. The OpiumPlex system discriminates most of the varieties from each other and pharmaceutical varieties from the others (culinary, dual and ornamental).

## Introduction

*Papaver somniferum* L. (opium poppy; Papaveraceae family), a traditional medicinal plant since the Neolithic age^1^, is the source of up to 140 alkaloids^2,3^, with morphine, thebaine, codeine, papaverine and noscapine being the most prominent. These alkaloids are used by the pharmaceutical industry for the development of medications utilized as pain relievers, cough suppressants, antispasmotic or antitumour agents or sedatives^4,5^. On the other hand, raw opium (latex obtained from the seed pods of *P. somniferum*) can be exploited as a starting material for the illicit manufacture of morphine or its semisynthetic derivate heroin.

The global production of illicit opium has seen an increasing trend and reached as high as 10 thousand tons in 2017, which is the largest amount recorded over the last two decades^6^. This alarming situation demonstrates another statistic provided by the United Nations Office on Drugs and Crime (UNODC): 29 million people used opiates in 2017, which is 50% more than in 2016^6^, although this shift is partially caused by an improvement in statistical methodology and the retrieval of new information from highly populated countries such as India and Nigeria. Nevertheless, these numbers lead to the conclusion that the situation is even worse than expected. Therefore, the great concern of governmental agencies about all aspects of drug use is not surprising.

One of the responses to the drug outbreak was the establishment of the Heroin Signature Programme (HSP) in many countries as part of their strategy for retrieving information on heroin trafficking and geographical origin^7^. Currently, heroin profiling mostly depends on advanced techniques of analytical chemistry, such as ultrahigh-performance liquid chromatography (UHPLC) or gas chromatography (GS), used in conjunction with different types of mass spectrometry (MS)^8,9^. Various combinations of highly sensitive techniques allow the identification and quantification of impurities arising during heroin processing along with the determination of the presence of adulterants^7^. Of course, a necessary foundation is the knowledge of alkaloid profiles of different opium poppy cultivars, which is also influenced by the variability of environmental conditions such as temperature^10^, soil type or seasonal distribution and total rainfall. An alternative approach based on the assessment of the isotope ratio of chosen elements was also proposed and successfully applied^11,12^.

Another way to retrieve more information involves molecular genetic methods, because opium or heroin contains plant (cell) parts and thus DNA, although in limited amounts and degraded^13^. However, there are two main obstacles: (1) problems with DNA isolation from opium or heroin and (2) a lack of appropriate DNA markers have prevented the application of these methods. The first obstacle was cleared when Marciano et al.^13^ announced successful DNA extraction from opium and heroin. Nevertheless, there is no forensically relevant DNA marker system implementing polymorphisms of short tandem repeats (STRs) or single nucleotide polymorphisms (SNPs).

The opium poppy genome has not been published until recently^14^. Therefore, most of the STR markers were developed by the data mining of sequences from transcriptomic studies^15–18^ or by screening sequences of unplaced genomic fragments retrieved via next-generation sequencing (NGS)^19^. Unfortunately, many of these markers suffer from one or more weaknesses, such as low polymorphism^20^, short lengths of repeated motifs or unknown localization on a chromosome. Therefore, the purpose of this study is to develop a new set of genomic STR markers with potential for application in the field of plant forensics.

## Material and methods

### Plant material

In this study, 187 accessions of individual poppy plants (Supplementary Table S1) and 44 “variety standards of poppy” (Supplementary Table S2; see further explanation) were analysed. This plant material comprises 36 varieties (Table 1) and two unregistered breeding materials of the opium poppy (*Papaver somniferum* L.), representing all recognized categories defined according to the main purposes of use (culinary, dual, pharmaceutical and ornamental^21^).

**Table 1:** List of analysed poppy cultivars.

Cultivar	Category	Country of registration^a^	Morphine [%]^b^ ^10,67–71^
Akvarel	Dual	CZ	0.61
Albín	Culinary	SK	0.33–0.39
Ametiszt	Culinary	HU	0.25
Aplaus	Dual	CZ	0.51–0.75
Bergam	Dual	CZ/SK	0.43–0.70
Buddha	Pharmaceutical	HU	1.80–2.20^c^
Danish Flag	Ornamental	–	0.10–0.25
Florian	Culinary	AT	0.14
Fortemo	Pharmaceutical	HU	1.20–1.60^c^
Frosted Salmon	Ornamental	–	–
Gerlach	Dual	CZ/SK	0.44–0.59
Kék Duna	Dual	HU	0.61–0.66
Korneuburger	–	–	–
Kozmosz	Dual	HU	0.37–0.67
Lazur	Pharmaceutical	PL	0.91–1.22
Lina	Pharmaceutical	HU	–
Major	Dual	SK	0.37–0.69
Malsar	Dual	SK	0.43–1.02
Maraton	Dual	CZ/SK	0.30–0.70
Marianne	Dual	NL	0.33–0.43
Morvital	Pharmaceutical	HU	1.90–2.30^c^
Morwin	Pharmaceutical	HU	1.00–1.40^c^
Onyx	Dual	CZ	0.77–0.85
Opal	Dual	CZ/SK	0.29–0.86
Opex	Dual	CZ	0.80–0.93
Orbis	Dual	CZ	0.72–0.92
Orel	Culinary	CZ	0.26–0.44
Orfeus	Dual	CZ	0.37
paeniflorum	Ornamental	–	–
Postomi	Pharmaceutical	HU	2.00–3.00^c^
Racek	Culinary	CZ	0.31–0.45
Redy	Culinary	CZ	0.16–0.24
Rosemarie	Dual	NL	0.43–0.61
Sokol	Dual	CZ	0.42–0.70
Tatransky	Culinary	SK	0.21
Zeno	Culinary	AT	0.38–0.49

^a^AT—Austria, CZ—Czech Republic, HU—Hungary, NL—Nederlands, PL—Poland, SK—Slovak Republic.

^b^Lowest and highest value found.

^c^Values provided by Research Institute for Medicinal Plants and Herbs Ltd.

Seeds were the starting material for all accessions, and plants intended for individual genotyping were cultivated in a greenhouse under regulated conditions. Each variety was grown in separate flowerpots containing ~ 10 seeds per variety. Later, several leaves from each plant were taken and placed into a 2 mL polypropylene column after 3–4 weeks of growth. This approach yielded 4–9 individual plant samples per variety.

To obtain better insight into intracultivar variability, another type of samples was prepared. These “variety standards of poppy” (VSP) were created after we acquired a sufficient number of seeds of the given variety. Approximately 200 seeds were put in moistened filter paper, placed in Petri dishes and left to germinate at room temperature for 3–5 days (the amount of time was cultivar-dependent). VSP were created by putting ~ 100–120 sprouts into one 2 mL column as a source material for DNA isolation. The whole procedure was repeated three times for each variety.

Both types of samples were frozen in liquid nitrogen and immediately used as a starting material for DNA extraction or stored at − 20 °C for later use. DNA extraction was performed using a DNeasy Plant Mini Kit (Qiagen, Hilden, Germany) according to the manufacturer’s recommendations. After the extraction, DNA samples were checked for purity and quantified via UV spectrophotometry with a S-111107AW nanophotometer (Implen, München, Germany). Moreover, the high molecularity of isolated DNA was verified by electrophoretic separation in a 1% (w/v) agarose gel. Depending on the purpose, the final concentration of the samples was unified to 5 ng per µL (preliminary screening, Sanger sequencing) or 1.25 ng per µL (capillary electrophoresis—CE).

Plant study with *Papaver somniferum* L. species was carried out in accordance with relevant institutional, national (Act No. 148/2003 Coll.; Decree No. 458/2003 Coll.) or international (Nagoya Protocol; International Treaty on Plant Genetic Resources for Food and Agriculture; Convention on Biological Diversity) guidelines and regulations. Pharmaceutical varieties provided by the Research Institute for Medicinal Plants and Herbs Ltd. were obtained in accordance with legislation of Hungary and the Czech Republic (The Ministry of Health of the Czech Republic, Inspectorate of Narcotic Drugs and Psychotropic Substances, certificate Nr. 27215/2019-2/OPL).

### Bioinformatic analysis and primer design

The nuclear genome of opium poppy cultivar HN1 (GCA_003573695.1)^14^ was downloaded from the genome database of the National Center for Biotechnology Information (NCBI). Searching for STR was performed with the help of Genome-wide Microsatellite Analyzing Tool Package v2.0 (GMATA)^22^ in a two-phase process. The first phase served as a global overview of microsatellite locus abundance, chromosomal density and distribution pattern according to motif length and number of repeats. The second phase was aimed at the selection of candidate loci, where we started with tetranucleotide STR, followed by pentanucleotide, tri and hexanucleotide microsatellites.

The GMATA algorithm only allows searches for perfect (pure) microsatellites, which were used for further evaluation. Moreover, only microsatellites with known localization on one of the eleven chromosomes were accepted. The GMATA setup was as follows: motif length 2–6 nt, with a minimum number of repeats 6 (for dinucleotide STR) and 5 (for tri to hexanucleotide STR). For the second phase, only microsatellites with motif lengths of 3–6 nt and number of repeats of 7 or more (tetranucleotide STR), 8 or more (penta to hexanucleotide STR) and trinucleotide STR within the range of 10–15 repeats were further processed. As the number of loci reached thousands of trinucleotide microsatellites, it would be time consuming to analyse them manually. Thus, a homemade R script^23^ was written for the raw selection of candidate loci. The optimization procedure of the most important parameters (degree of best hit masking, total count of sequences producing significant alignment and number of sequences with high query cover) was based on a training dataset containing approximately 400 manually evaluated loci. A file with preselected trinucleotide microsatellite loci could be downloaded (Supplementary Table S3), and the script is available upon request from the authors.

Each locus identified during the second phase was aligned against the whole poppy genome using BLASTN v2.10.0 + ^24^ with algorithm settings for highly similar sequences (megablast) and more dissimilar sequences (discontinuous megablast). The retrieved results were taken into consideration as an important selection criterion for candidate markers and during the primer design process to ensure single locus specificity. Furthermore, GMATA software was utilized for primer design (settings: T_m_ = 60 °C, amplicon size 100–300 bp) at the initial stage, and new primers were prepared with the help of the software Primer3web v4.1.0^25,26^ and OligoEvaluator (an online web tool provided by Sigma-Aldrich—http://www.oligoevaluator.com/Login.jsp) under the same conditions. Loci without primers proposed by GMATA were excluded from further analysis.

### Preliminary screening

The chosen loci were amplified in a singleplex PCR arrangement via a C-1000 (Bio-Rad, Hercules, CA, USA) or T-gradient Thermo (Analytic Jena, Jena, Germany) thermocycler and tested on 8 samples. The PCR mixture (10 µL of final volume) contained 5 ng of DNA, 0.2 µM F and R primers and 1 × Multiplex PCR Master Mix Plus (Qiagen). The amplification conditions were as follows: one cycle of initial denaturation at 95 °C for 10 min, followed by 35 cycles of denaturation at 94 °C for 30 s, annealing at 60 °C for 90 s and elongation at 72 °C for 60 s. The final step comprised the final elongation at 72 °C for 10 min.

Amplification products were electrophoretically separated in a 5% (w/v) agarose gel, and the results were visualized and documented with the GelDocXR system (Bio-Rad) after 3 h of separation. Only markers with one band and a clear profile were used for the next step of analysis (sequencing) described in the Sanger sequencing section.

### Singleplex PCR and CE

Markers for separation in CE were amplified in a similar manner as candidate markers for preliminary screening. Only a few changes were made—the amount of DNA was lowered to 1.25 ng per reaction, and the number of cycles was decreased to 31. Furthermore, the temperature and time for the final extension were changed from 72 °C for 10 min to 60 °C for 80 min to eliminate split peaks, and F primers were labelled with 6-FAM, VIC, NED or PET fluorescent dye. The next step included pooling four differently labelled markers and dilution with double deionized water at a ratio of 1:1:1:1:26 before CE. Then, 1 µL of PCR product was added to 12 µL of Hi-Di formamide (Thermo Fisher Scientific, Waltham, MA, USA) with 0.2 µL of GeneScan LIZ600 Dye Size Standard v2.0 (Thermo Fisher Scientific) and denatured at 95 °C for 5 min. The samples were separated in an ABI PRISM 310 Genetic Analyser (Thermo Fisher Scientific) using a 47-cm-long capillary filled with POP4 polymer. The following setup was applied: temperature 60 °C, injection 7 kV for 5 s, run 15 kV for 24 min, dye set G5. Fragment analysis and allele identification were performed with the help of the program GeneMapper v4.1 (Thermo Fisher Scientific).

The first screening included only 4 samples to check peak morphology, the occurrence of nonspecific amplicons and the profile as a whole. Then, the second screening of 41 reference samples (32 samples of *P. somniferum*, 9 samples of species *Papaver* and *Argemone* genera) was performed to estimate the polymorphism and specificity of each candidate marker. At least 3 alleles within *P. somniferum* samples were required to accept the tested microsatellite as a marker.

### Multiplex PCR optimization and CE

Three multiplex assays (MTP1-3) were designed on the basis of the number of markers, size ranges and potential for labelling by four different fluorochromes. To avoid excess primer-primer interactions, an online version of AutoDimer software^27^ was used with the settings T_m_ = 60 °C and score 5 or lower as a threshold for including markers in one of the three assays. The optimization procedure was mainly focused on interloci balance via changes in primer concentrations (tested range 0.08–0.4 µM) and the optimal number of PCR cycles (tested range 28–31 cycles). It was found that several markers suffered from a higher occurrence of split peaks in the multiplex arrangement, although this problem had not been observed before. To avoid this problem, a pig tail sequence (5′ GTTTCTT 3′)^28^ was added to each reverse primer to retain interloci distances.

PCR master mix with 10 µL of total volume contained 1.25 ng of DNA, one of the assay sets of F and R primers with various concentrations and 1 × Multiplex PCR Master Mix Plus (Qiagen). Optimized PCR started with 1 × initial denaturation at 95 °C for 10 min, followed by 28 cycles of denaturation at 94 °C for 30 s, annealing at 60 °C for 90 s, and elongation at 72 °C for 60 s, and a final elongation at 60 °C for 80 min. PCR products were diluted 1:9 with ddH_2_O, and 1 µL was added to 12 µL of Hi-Di formamide (Thermo Fisher Scientific) with 0.2 µL of GeneScan LIZ600 Dye Size Standard v2.0 (Thermo Fisher Scientific). The mixture was denatured at 95 °C for 5 min and separated in an ABI PRISM 310 (Thermo Fisher Scientific) under the same conditions as described before. The coincidence of several events (ABI 310 failure, COVID-19 outbreaks leading to university lockdown and discovery of null alleles—see “Results and Discussion”sections) forced us to finalize the analysis with a different CE instrument. Therefore, the second separation of all samples was performed on an ABI 3500 (Thermo Fisher Scientific) with the following settings: temperature 60 °C, injection 3 kV for 5 s, run 19.5 kV for 40 min., dye set G5. One microlitre of each PCR product was separated in a 50-cm-long capillary filled with POP7 polymer. Moreover, SeqStudio Genetic Analyser (Thermo Fisher Scientific) was also utilized for comparing of spectral calibration on various CE instruments with the following set-up: injection 1.6 kV for 7 s, run 19.5 kV for 45 min, dye set G5. One microlitre of each PCR product was separated in POP1 polymer.

The optimization of multiplex PCR was performed using a set of 7 samples, which served as positive and run-to-run precision controls during the analysis of a large group of samples. Moreover, one sample serving as a negative control and 1 µL of appropriate allelic ladder injected 3 times per run were also included.

### Sanger sequencing—preliminary screening and allele sequencing

Two samples per candidate marker were selected for direct PCR sequencing during the preliminary screening phase. The PCR profile and master mix compound were the same as described in the Preliminary screening section*,* but 3 PCR tubes per sample were amplified. The obtained PCR products were separated in a 1% (w/v) agarose gel, excised with a clean scalpel after 30–40 min of separation, and purified using a GeneJET Gel Extraction Kit (Thermo Fisher Scientific).

A similar approach was applied to the allele sequencing of homozygotes for the purpose of standardization. There was only one substantial change, as modified primers with M13 tails (F primer—5′ TGTAAAACGACGGCCAGT 3′ + locus specific sequence; R primer—5′ CAGGAAACAGCTATGACC 3′ + locus specific sequence) were used to obtain whole amplicon sequences including the primer regions.

Several alleles were only found in heterozygotes, and the size difference between the alleles was often too small to use standard agarose gel electrophoresis. Thus, acrylamide gel electrophoresis (i) or TA cloning (ii) was used instead.

(i) Samples and the internal size standard were separated in a 5% denaturation (7 M urea) polyacrylamide gel (19:1). Separation was performed in a vertical cell Sequi-Gen (Bio-Rad) in 0.5 × TBE buffer at 45 °C and 15 W for 3.5 h. The preparation of the samples included mixing with a denaturation dye^29^ in a 1:1 ratio, heating at 95 °C for 5 min, and leaving on ice until loading onto the gel. The desired fragments were carefully scratched from the gel after silver staining^29^ and left overnight in PCR columns with 20 µL of ddH_2_O. Four microlitres of water with released DNA was reamplified and purified via agarose gel separation and extraction as described earlier.
(ii) Purified gel amplicons were ligated with pGEM-T Easy Vector System I (Promega, Madison, WI, USA), incubated overnight at 4 °C, and transformed into DH11S competent cells using the heat shock method. The selection of colonies with the insert was made by blue/white screening, where bacteria were grown on LB medium containing ampicillin (100 µg/mL), IPTG (0.5 mM) and X-Gal (80 µg/mL). The presence of the insert was verified by colony PCR, and plasmids with the insert were extracted with a GeneJET Plasmid Miniprep Kit (Thermo Fisher Scientific).

Regardless of the method used, DNA fragments of all samples were quantified via UV spectrophotometry, and DNA concentration was adjusted according to the requirements of the sequencing service provider (Eurofins Genomics Germany GmbH, Ebersberg, Germany) and bidirectionally sequenced. There was one exception where ligated alleles were only sequenced with the forward M13 primer.

A quality control check of the raw data, both visual and according to the quality value (QV), was carried out with the help of Sequencing Analysis Software v5.2 (Thermo Fisher Scientific). Further manipulation comprising sequence assembly, multiple alignment against the reference sequence and a necessary manual correction was carried out in the software BioEdit v7.0.9.0^30^ and the online version of MUSCLE software^31^, available on the webpage of The European Bioinformatics Institute (EMBL-EBI).

### Allelic ladder construction and nomenclature establishment

The first generation of allelic ladders (ALs) was created by the individual amplification of each allele (same PCR conditions as for preliminary screening), mixing all alleles within each locus and dilution to 1:19 with ddH_2_O. These “within locus” mixtures served as a starting material for a series of optimization steps aimed at balancing intra- and interlocus signals. The second-generation ladders were created via reamplification using 1 µL of diluted PCR product (1:50) from the previous amplification. The obtained amplicons were diluted and treated in the same manner as the first-generation ladders.

The allele nomenclature followed international forensic guidelines^32–34^ and is in accordance with the “one-change-rule” mentioned by Butler^35^. Moreover, short identifier (SID) labels^36^ for sequence-based nomenclature were created.

### Internal validation

#### Species specificity

The specificity of the markers was tested on both plant (9) and animal (7) species. Animal species included human (*Homo sapiens sapiens* L.), dog (*Canis lupus* f. *familiaris* L.), cat (*Felis catus* L.), horse (*Equus caballus* L.), pig (*Sus scrofa* f. *domestica* L.), domestic ferret (*Mustela putorius* f. *furo* L.), and Colorado potato beetle (*Leptinotarsa decemlineata* Say). Plant samples included hop (*Humulus lupulus* L.), potato (*Solanum tuberosum* L.), apple (*Malus domestica* Borkh), Mexican poppy (*Argemone mexicana* L.), and several closely related species represented by Turkish red poppy (*Papaver glaucum* Boiss. & Hausskn.), Iceland poppy (*Papaver nudicaule* L.), oriental poppy (*Papaver orientale* L.), corn poppy (*Papaver rhoeas* L.), and Persian poppy (*Papaver bracteatum* Lindl.) All samples were provided by our colleagues as purified DNA samples with the exception of *A. mexicana* and species belonging to the *Papaver* genus, which were obtained and treated in the same way as *P. somniferum* samples (see “Plant material”section).

#### Balance metrics

Three balance metrics were calculated: interloci balance (IELB), intracolour balance (ICB) and heterozygote balance (Hb), also known as peak height ratio (PHR). Interloci balance was calculated as the peak height at a given locus divided by the mean peak height across all loci in a given assay^37^. ICB was determined as the lowest rfu value across all loci labelled with the same dye divided by the highest rfu value across all loci labelled with the same dye^38^. Data normalization reflecting the codominant status of the used markers preceded ICB calculation, where two peaks in heterozygotes were averaged and peaks in homozygotes were halved^38^. The last metric, Hb, was calculated according to both commonly used methods^39–41^. The first method is defined as:

1 $${\text{Hb}} = {\text{h}} =\upphi _{HMW} /\upphi _{LMW}$$

where Hb denotes heterozygote balance, HMW—high molecular weight allele, LMW—low molecular weight allele, ϕ—peak height. The second method is defined as:

2 $${\text{Hb}}^{\prime } = {\text{h}}^{(2)} =\upphi _{smaller} /\upphi _{larger}$$

where Hb′ denotes heterozygote balance, smaller—smaller peak height, larger—larger peak height and ϕ refers to peak height. Both equations and terminology were adopted from Kelly et al.^40^. For the metrics mentioned above, the mean, median, standard deviation (SD), minimum and maximum (mean ± 3*SD) were calculated.

#### Sensitivity test

The samples (n = 21), chosen according to the highest number of heterozygous loci, were serially diluted to five different DNA concentrations (b–f variant): 1.25 ng/µL (b), 0.625 ng/µL (c), 0.3125 ng/µL (d), 0.1563 ng/µL (e) and 0.0781 ng/µL (f) in nuclease-free water (Promega). First, the concentration of stock solution (a variant) was measured with a Qubit dsDNA HS Assay Kit in a Qubit fluorimeter (Thermo Fisher Scientific) according to the manufacturer’s recommendations. The concentration of diluted samples was also measured by fluorimetry as described above and by digital droplet PCR (ddPCR) using a QX200 Droplet Digital PCR System (Bio-Rad). The ddPCR mixture (40 µL final volume) included 1 × QX200 EvaGreen ddPCR Supermix (Bio-Rad), each pair of primers at 0.1 μM (F-OPTET063, Rpig-OPTET063) and 1 μL of DNA (variant b–f). The ddPCR conditions followed the manufacturer’s instructions with one modification: the addition of an extension step (72 °C, 30 s) after annealing (at 58 °C). The results were analysed with QuantaSoft v1.7.4 (Bio-Rad).

#### Stutter ratio

For the purpose of stutter analysis, all parameters for the stutter filter were set to 0, and the minimum rfu signal was lowered to 50 rfu for homozygotes and 25 rfu for heterozygotes in GeneMapper v4.1 (Thermo Fisher Scientific). Stutter peak size was characterized numerically using the stutter ratio (S_R_) formula^42^:

3 $$S_{R} =\upphi _{{\text{S}}} /\upphi _{{\text{A}}}$$

where ϕ_S_ is the height of the stutter peak, and ϕ_A_ is the height of the allelic peak.

The stutter ratios of forward (n + k; k = one repeat unit), backward and double backward stutters (n − k and n − 2 k) were determined for each marker (where applicable). Considering unexpected stutter profiles with a series of n − 1 stutters observed for OPPEN22 and OPPEN09 markers, up to three stutter ratios (n − 2/3/4 bp) were calculated. It is worth mentioning that for many alleles/samples, no stutter was observed or recognized by the program algorithm. Therefore, such samples were excluded from this analysis, although this would substantially lower the stutter ratio.

#### Precision and accuracy test

Each of the three allelic ladders was injected at least 9 times (range 9–14 depending on the ladder) and separated to determine the sizing precision and accuracy of the developed assays. The mean size and SD for each allele were calculated, and the result was summarized as the averaged SD along with the standard error of the mean (SEM) and minimum and maximum size SD.

### Descriptive statistics and population analysis

The genetic variability of all individual samples was described by several basic parameters, such as the number of alleles (N_al_) and their frequency or observed heterozygosity (H_Obs_). Furthermore, to establish the degree of similarity among the varieties, identity analysis (ID), principal component analysis (PCA) and cluster analysis (CLU) were used as exploratory methods. ID analysis was performed by a series of pairwise comparisons of all individual samples with 0 allele mismatches using the program Cervus v3.0.3^43^. Functions implemented in adegenet v2.1.3. package^44^ were utilized for data preparation, whereas PCA itself was performed in the ade4 v1.7-15 package^45,46^. CLU analysis on 3 latent variables (PC axes) was done using the UPGMA clustering algorithm in order to depict both cultivar category and country of registration. The matrix with euclidean distances and graphical visualization was made using functions in the packages amap v0.8-18^47^ and ComplexHeatmap v2.6.2^48^.

## Results

### Bioinformatics and marker development

The assembled poppy genome (2.72 Gb)^14^ contains approximately 150 thousand microsatellite loci (Supplementary Fig. S1), with mean microsatellite density ranging between 53 and 58 loci per Mb (Supplementary Fig. S2). Over 96% of loci are comprised of di- and trinucleotide microsatellites. Therefore, microsatellites with longer motifs (tetra- to hexa-) account for only a few thousand loci (Supplementary Fig. S1). Moreover, consecutive analysis of microsatellites sorted according to the number of repeats (NR) showed a sharp decrease in the number of loci with higher NR (Supplementary Fig. S3).

**Figure 1:**
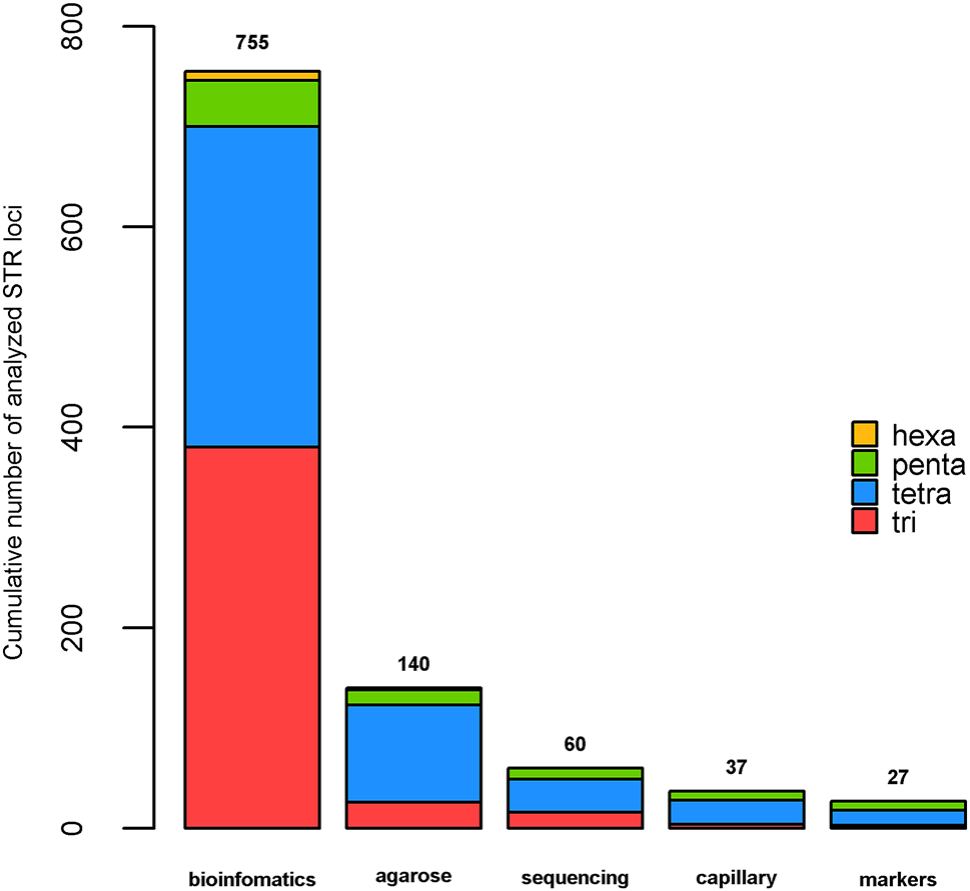
Overview of marker development. Tri to hexa refers to the proportion of tri- to hexanucleotide microsatellites. Created in R v4.0.3 (https://cran.r-project.org/)^23^.

The whole process of marker development consisted of several quality control steps (Fig. 1). It is evident, that more than 80% of the microsatellites were excluded in the initial phase of the process, and the overall success rate was 3.6% or 1:29 (accepted:discarded markers). The primary targets were tetranucleotide microsatellites with higher NR, where 57 (NR ≥ 10), 91 (NR = 8–9) and 172 (NR = 7) candidate loci were found, but only 15 of them (4, 9 and 2, respectively) became part of the STR profiling system (Supplementary Table S4). By subsequent analysis of all penta- (46) and hexanucleotide (9) microsatellites with NR ≥ 8 obtained nine pentanucleotide markers and no hexanucleotides. Finally, the group of 24 markers was extended by three trinucleotide microsatellites to maximize the total number of markers. Since trinucleotide microsatellites represent the most frequent type, many more candidate loci were available (Supplementary Table S3), but their usable number was intentionally limited by the requirement of weak or no linkage.

**Figure 2:**
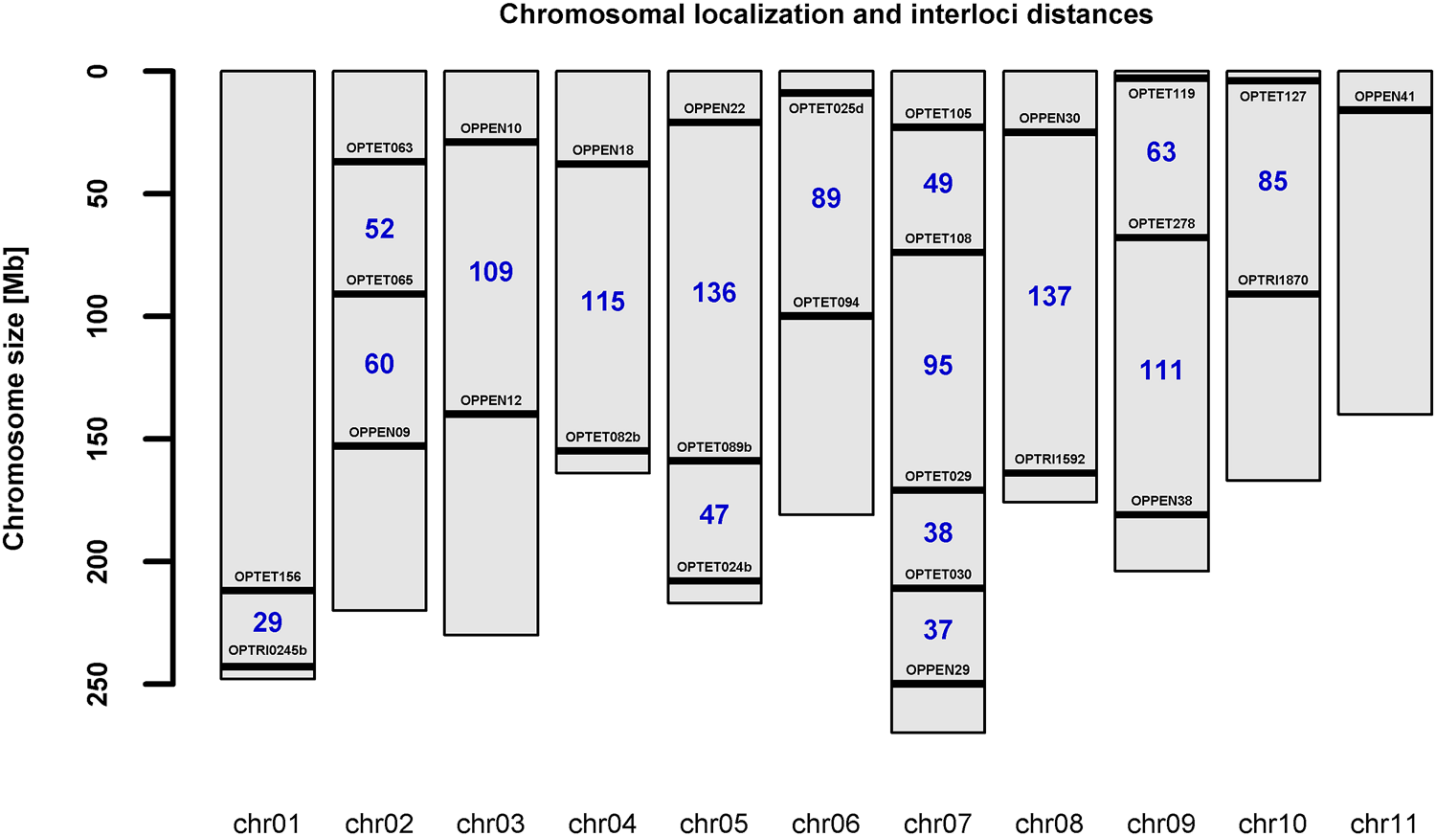
Chromosomal localization of the markers and interloci distances. Created in R v4.0.3 (https://cran.r-project.org/)^23^.

Most of the new markers are located more than or close to 50 Mb (Fig. 2) from each other, and it is probably safe to assume independent or nearly independent segregation in these cases. On the other hand, a weak to moderate linkage can be expected between the markers OPTET029 and OPTET030 (38 Mb), OPTET030 and OPPEN29 (37 Mb), and OPTET156 and OPTRI0245b (29 Mb), as it was not always possible to meet the 50 Mb distance requirement with regard to other parameters influencing marker choice.

### Allelic ladders and proposed nomenclature

Three allelic ladders containing 151 out of 158 (~ 96%) alleles were created via the combination of appropriate samples (Supplementary Figs. S4, S5 and S6, respectively). The exclusion of some alleles was caused by an unsuitable combination of genotypes or lack of the sample DNA consumed during the testing phases and sequencing. The nomenclature for most of the alleles was straightforward (Supplementary Presentation S1), but there were a few cases requiring special treatment. For three alleles, the nomenclature would be impractically long and thus a shortened nomenclature that preserve most of the information was proposed. The first synonymous name 6.2 (D1-9del; D33TCdel) is for allele 6.2 (D1AGATAAAGAdel; D33TCdel) of locus OPTET089b (Table XVII in Supplementary Presentation S1), the second name 3 (U11-10del) is a substitution for allele 3 (U11TGTTAAAATGdel) of locus OPTRI0245b (Table XXV in Supplementary Presentation S1), and the last synonymous name 14 (U25-8del) is for allele 14 (U25TTGACCGAdel) of locus OPTRI1592 (Table XXVI in Supplementary Presentation S1).

An alternative nomenclature using SID labels was also established (Supplementary Table S5).

### Null alleles

An unusually high prevalence of missing signals in several loci (OPPEN22, OPPEN38, OPTET119, OPTRI0432b and OPTRI1592) exhibiting a cultivar-dependent pattern was noticed. This nonrandom distribution supported the assumption of the occurrence of mutations in one or both priming sites leading to the null allele. Therefore, new sequencing primers localized upstream of F and downstream of the original R primer were designed to reveal the true cause of the null alleles (Supplementary Table S6).

Locus OPPEN22 contained the substitution G- > A next to the last base of the 3′ end of the F primer (Table V, Fig. V in Supplementary Presentation S1), and locus OPTRI0432b even had two substitutions (C- > T, G- > A) in the F primer site and one substitution (C- > A) in the R priming site (Supplementary Fig. S7). The situation at locus OPTRI1592 was substantially different, where an 8-nucleotide deletion was found (Fig. XXVI in Supplementary Presentation S1). On the other hand, the sequencing of OPTET119 showed no changes in the primer regions but revealed unexpectedly long stretches of microsatellite repetitions leading to size overlap with the OPPEN09 locus labelled with the same dye. For the last tested locus (OPPEN38), all attempts to obtain locus-specific amplicons suitable for sequencing failed, despite many trials with 3 different primer sets and all of their possible combinations (Supplementary Table S6).

### Internal validation

#### Species specificity

Nonspecific peaks were detected in 13 out of 27 markers (~ 48%) during the singleplex screening with 5 closely related species of the *Papaver* and *Argemone* genera. The number of loci with nonspecific peaks was seemingly higher (14) when multiplex assays were used, but in the tested dataset, *P. bracteatum* was newly included (we obtained it in the later phase of the project), which substantially increased the number of amplifying loci (without *P. bracteatum,* the number of amplifying loci was 10). Moreover, most of the detected signals were close to the set analytical threshold (200 rfu), and therefore very weak when compared to the typical signal of *P. somniferum* (several thousand rfu—Supplementary Table S7).

For other tested samples of both the Plantae and Animalia kingdom, cross-reactivity was not observed, which is primarily important for human samples as the main potential source of contamination.

#### Interloci balance, intracolour balance and heterozygote balance

Overall mean interloci balance (IELB) values were 1.07 ± 0.2 (MTP1), 1.09 ± 0.29 (MTP2) and 1.06 ± 0.18 (MTP3), respectively. Mean IELB values for individual loci ranged between 0.55 and 1.25 for MTP1, between 0.49 and 2.26 for MTP2 and between 0.78 and 1.65 for MTP3 (Supplementary Table S8, Fig. S8 upper row). Further, the influence of the spectral calibration was studied, based on the observed contrast between the relative signal strength of some dyes in the ABI 310, ABI 3500 machines and for comparison purposes on SeqStudio as well. Observed differences are demonstrated for the same sample, where increased signal in the yellow and red dye channels and decreased signal in the blue and green dye channel on the ABI 3500 are clearly visible for all multiplex assays (Supplementary Fig. S9).

This situation complicated the analysis on ABI3500, because it led to a 2 × times stronger signal above the average in OPTET025d marker (MTP2 assay) and the potential risk of an “off-scale” signal. To obtain the optimal result for ABI 3500, the concentration of OPTET025d primers was changed from 0.2 to 0.1 μM, and OPTET105 primers from 0.2 to 0.3 μM. The modified MTP2 assay was subsequently retested on 46 randomly selected samples. This led to some improvement according to the results (Supplementary Table S9, Fig. S10). Therefore, we suggest using the modified version of MTP2 for ABI 3500 machines with slightly different primer concentrations.

For intracolour balance (ICB), an overall mean of 0.74 was reached for MTP1 with a range of 0.61–0.84, an overall mean of 0.66 for MTP2 with a range of 0.49–0.75, and an overall mean of 0.73 for MTP3 with a range of 0.66–0.76 (Supplementary Fig. S8 bottom row).

Exploratory data analysis revealed several outliers. Therefore, 8 (5 for MTP1, 1 for MTP2 and 2 for MTP3) samples were excluded before the heterozygote balance was calculated. These outliers could strongly bias the results (mostly for the Hb method), as the average number of heterozygotes per locus was limited (n ~ 11). According to the Hb method, the mean values ranged from 0.83 to 1.07 across all loci, while according to the Hb′ method, values ranged from 0.75 to 0.93 across all loci (Supplementary Table S8, Supplementary Fig. S11).

#### Sensitivity

The sensitivity of the three multiplex assays was tested on 21 highly heterozygous samples diluted to 1.25 ng, 0.625 ng, 0.313 ng, 0.156 ng and 0.078 ng DNA per reaction. A closer look at the data revealed one non-amplifying locus for the variant with 1.25 ng of DNA and one case of multiple non-amplifying loci for the variant with 0.625 ng of DNA. A full profile was observed in 18 out of 21 samples for variants with 0.313 ng of DNA, but an elevated number of locus dropouts (3x) and allelic dropouts (1x) occurred. The final dilutions (0.156 and 0.078 ng of DNA) were highly error prone, where both locus dropout and allelic dropout were detected for half or more samples. Moreover, the analysis was complicated by the presence of background noise with peaks at 200–300 rfu at several loci.

Highly heterozygous genotypes also allowed us to analyse changes in heterozygote balance connected to the amount of DNA in the reaction (Supplementary Table S10 and S11). A general trend across the loci leads to a poorer balance according to the Hb′ method (overall means 0.91–0.65) and surprising preferential amplification of the high molecular weight allele (Hb overall means 0.97–1.3). Furthermore, a lower amount of DNA was connected with greater variability between the samples (Hb SD 0.11–0.72; Hb′ SD 0.06–0.2), as stochastic effects start to play a major role. Nevertheless, it is necessary to take into account the limitations of these results given by the small number of heterozygotes per locus (n ~ 6).

#### Stutter ratio

We paid attention to the backward stutter (n − k), as it is the most prevalent type, but other types were also analysed, namely, double backward (n − 2 k) and forward (n + k) stutters. Moreover, two pentanucleotide loci (OPPEN09 and OPPEN22) produced an unusually complex profile with an array of consecutive n − 1 stutters, which was noticed on ABI 3500 and confirmed on SeqStudio thanks to the better separation capabilities of the POP7 and POP1 polymers (compare the last NED peak in Supplementary Fig. S9 part a vs b or c and the second VIC peak in Supplementary Fig. S9 part g vs h or i). The mean stutter ratio of the backward stutter ranged from 4.23 to 8.65% for the trinucleotide markers, from 1.36 to 7.01% for the tetranucleotide markers and from 1.92 to 9.07% for the pentanucleotide markers (Supplementary Table S12).

To obtain further knowledge about stutter behaviour, we also characterized allele-specific stutters. Roughly two-thirds of markers followed a trend of an increased stutter ratio with increasing allele size, i.e., with the number of repeats (Supplementary Figs. S12, S13 and S14, respectively), although not necessarily in a linear fashion (see OPTET024b in Supplementary Fig. S13 and OPTET082b in Supplementary Fig. S14).

#### Precision and accuracy test

The analysis was performed on both capillary machines (ABI 310 and ABI 3500) using the first generation of allelic ladders. A precision test on 144 alleles (older version of allelic ladders) separated on ABI 310 led to an average standard deviation of 0.059 (SEM = 0.005) across all loci. The minimum SD value (0.019) was detected for allele 14 of the locus OPTET025d, whereas the maximum SD value (0.218) was detected for allele 16 of the locus OPTRI1870. Separation of the final version of the allelic ladders with 151 alleles in the ABI 3500 led to similar results, with an average standard deviation of 0.068 (SEM = 0.006) across all loci. Minimum and maximum SD values reached 0.032 (allele 8.2 of the locus OPTET089b) and 0.109 (allele 6 of the locus OPTET119), respectively. The average values of three standard deviations (0.177 for ABI 310 and 0.204 for ABI 3500) were roughly within the default binning range (± 0.4 bp), but some alleles could still exhibit more dynamic mobility, as demonstrated by allele 16 of locus OPTRI1870. Therefore, our results confirm the necessity of using an allelic ladder with every run, as is generally recommended^49^.

### Descriptive statistics and population study

158 alleles were found across all loci, and most of the alleles (~ 96%) were characterized by both fragment analysis and Sanger sequencing (Supplementary Table S13). The number of alleles varied from 3 to 14, with a mean of 5.85 (median 5) alleles per locus and observed heterozygosity per locus ranged from 0.022 to 0.128 with a mean of 0.076.

Pairwise comparison showed that the new system is capable of recognizing 30 out of 36 varieties plus both new breeding materials, and revealed the existence of two indistinguishable groups. One group comprises four varieties (Sokol, Racek, Orel and Akvarel), and the second group comprises two other varieties (Postomi and Buddha).

**Figure 3:**
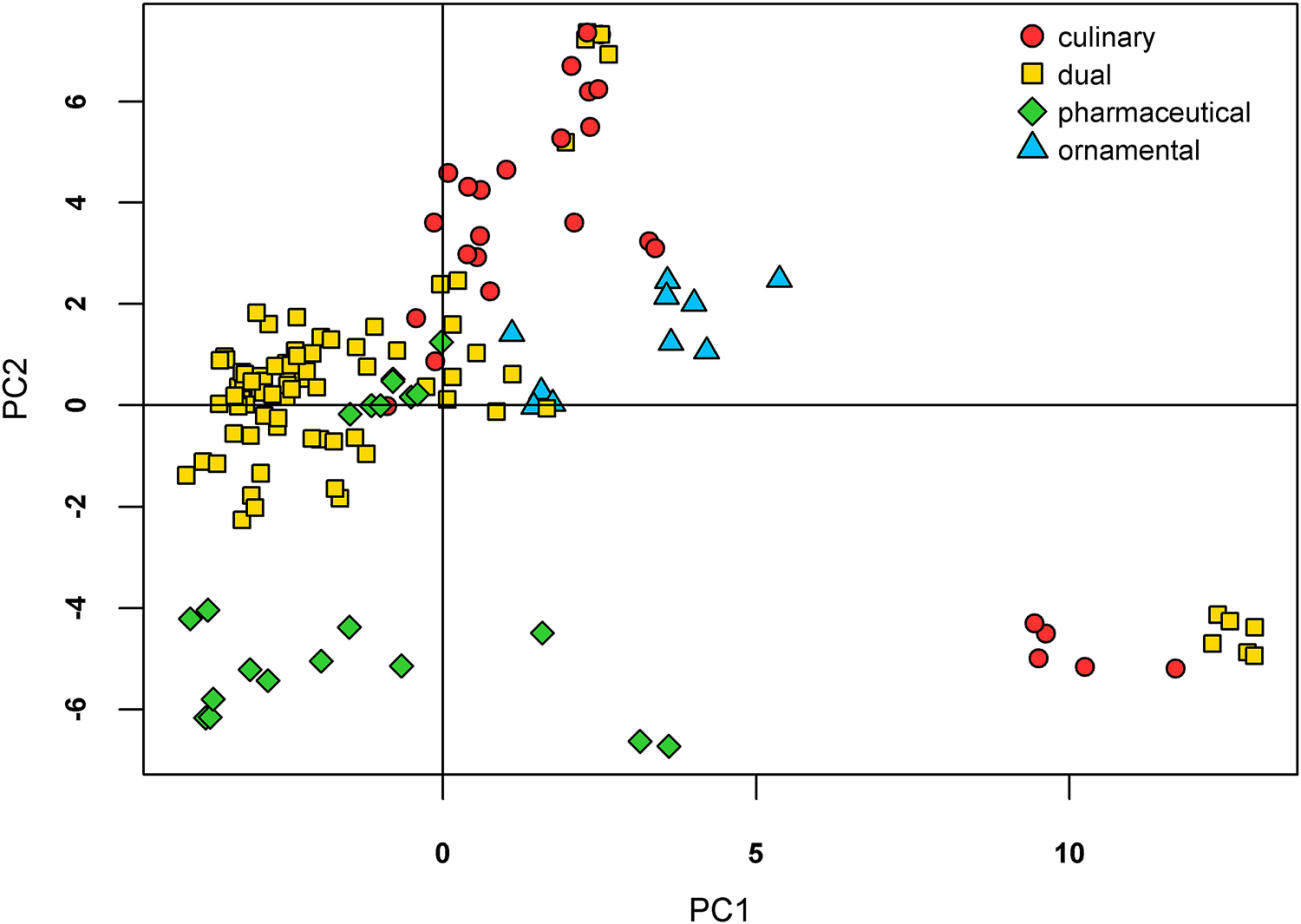
Exploratory analysis via PCA. The projection of two synthetic variables (PC1 and PC2) onto the factorial plane is shown. Created in R v4.0.3 (https://cran.r-project.org/)^23^.

**Figure 4:**
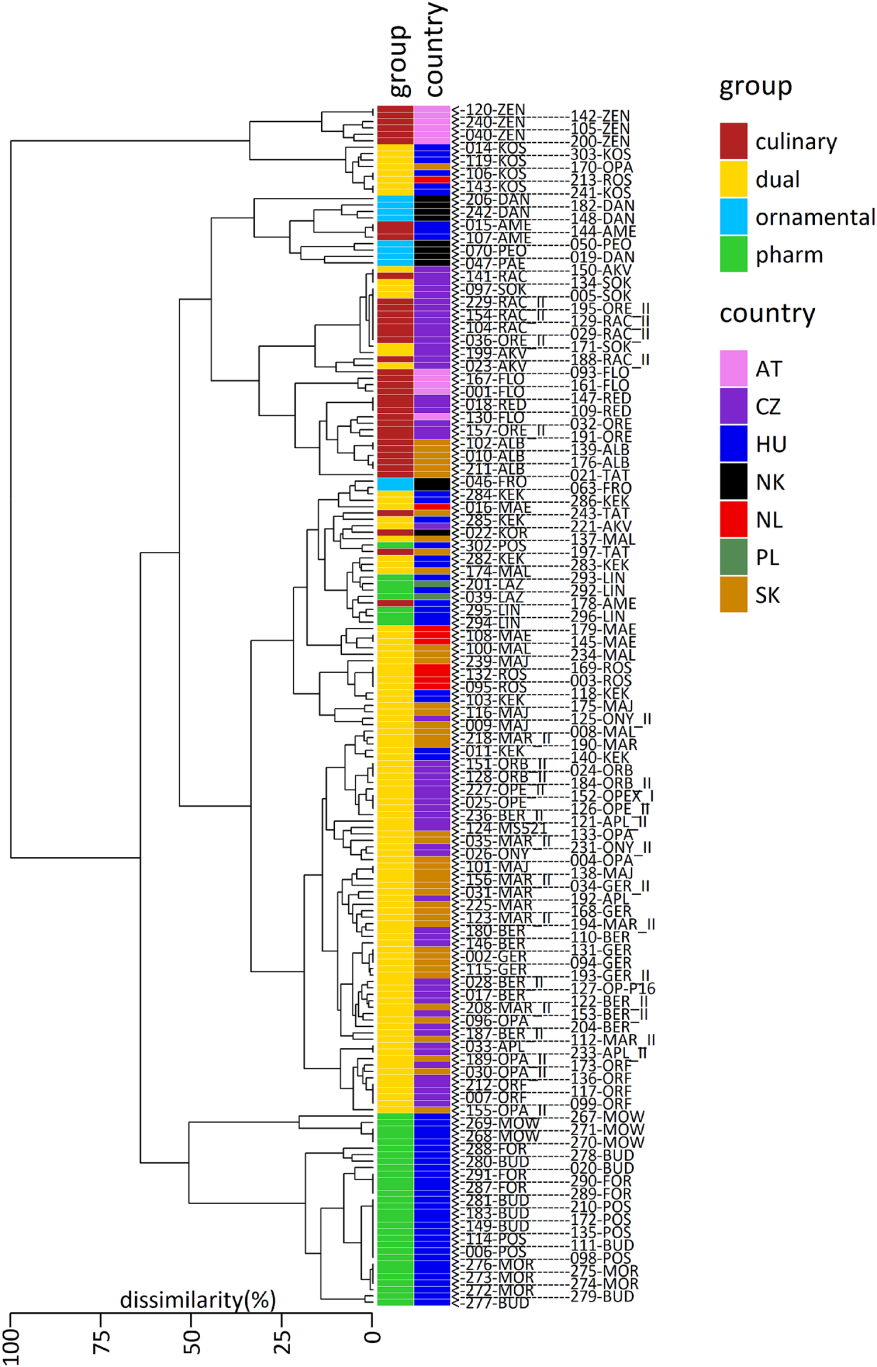
Exploratory analysis via CLU. AT—Austria, CZ—Czech Republic, HU—Hungary, NK—not known, NL—Nederlands, PL—Poland, SK—Slovak Republic. List of individual genotypes can be found in Supplementary Table S1. Created in R v4.0.3 (https://cran.r-project.org/)^23^.

Analysis by the PCA method enabled the identication of several clusters of varieties (Fig. 3). The first principal component separated two extremely distincts varieties, Zeno and Kozmosz (Fig. 3; bottom right), and delimited the groups of ornamental, culinary and dual poppies. More importantly, the second principal component almost completely separated all pharmaceutical varieties from the others, with the exception of the Lina and Lazur variety. On the other hand, a group of accessions belonging to all recognized categories was detected (Fig. 3; coordinates [0,1]). PCA was unable to separate this group with the third principal component either (data not shown), which catches some variability within the identified groups. CLU analysis showed that varieties are clustered more according to recognized categories of purpose than country of registration (Fig. 4). It also helped to determine some mislabeled genotypes such as sample nr. 302, recognized as Postomi variety (POS), or nr. 170, labeled as Opal variety (OPA), which were clearly different from the other samples of the given variety (Fig. 4).

## Discussion

### Bioinformatics and marker development

Bioinformatic analyses led us to the conclusion that the number of available microsatellite loci with desired characteristics is low and confirmed the considerably limited potential for the creation of de novo markers with regards to typical forensic standards. Moreover, most of the remaining candidate loci were excluded during the marker development. The main reason was the multicopy nature of many loci, and their high similarity often reaching 98–99%, caused by the occurrence of relatively recent whole-genome duplication dated to 7.8 ± 4.35 million years ago^14^. This situation led to difficulties with the design of single locus-specific primers and prevented precise the planning of amplicon sizes for highly multiplexed STR systems.

The total number of markers (27) represents a compromise between the effort to maximize discriminatory power, as we were aware of the relatively low genetic variability reported for opium poppy^16,18,19^, and obtain independently segregating loci. Unfortunately, there is a lack of information on the recombination rate in opium poppy in the literature, and it can substantially vary among species, chromosomes of the same species or even a chromosome itself^50–52^. Therefore, a rather conservative value of 1 cM = 1 Mb was chosen, and we can expect that the majority of the loci meet the independent segregation criterion. Nevertheless, it is still necessary to take into account that the real recombination rate is unknown and remains to be determined in the future when more information becomes available.

### Proposed nomenclature

First, it is necessary to mention that we accepted a nomenclature system according to Gusmão et al.^34^ that distinguises the source of the length variation within the microsatellite repetition itself and within the flanking regions. Although the authors only mentioned this rule for intermediate alleles, i.e., alleles with additional variation to the integral number of repeats, it should also be valid for other situations to preserve logical consistency. For example, we detected two alleles with sizes of 184 and 186 bp in the locus OPTET089b with the same repeat structure (AAGA)_7_AG(AAGA)_1_, but the smaller allele contained dinucleotide deletions downstream of STR repeats. Following Gusmão et al.^34^, the smaller variants were named 8.2 (D42TCdel), and the larger variant was named 8.2. This example shows how an indel (in this case TC) in the flanking region “masked” incomplete repeats (in this case AG). Therefore, if we accept the view of authors such as Valverde et al.^53^ or Eichman et al.^54^, the nomenclature would change to 8 (smaller allele) and 8.2 (larger allele) despite the same repeat structure. We prefer the logic of Gusmão et al.^34^ for several reasons, although it seems to be less practical than the nomenclature according to the aforementioned authors. Our opinion is that this approach provides more information about the alleles and better reflects the true nature of the repeat structure. More importantly, there are cases where this is the only nomenclature that makes sense. We can demonstrate it for locus OPTRI0245b with two alleles containing the same repeats (AGA)_3,_ but one of the alleles also has a 10-nucleotide long deletion in the flanking region (Table XXV, Fig. XXV in Supplementary Presentation S1). This allele could only be named according to Gusmão et al.^34^ because according to Valverde et al.^53^ or Eichman et al.^54^, the subtraction of 10 nucleotides would completely eliminate whole microsatellite repeats and even lead to negative numbering. Nonetheless, we decided to create an alternative nomenclature based on recently developed SID labels^36^. This nomenclature has two advantages. The first one is optional shorthand nomenclature for routine analysis, which could be used instead of or along with the suggested nomenclature. The first three letters alone uniquely identified any detected allele in our study, and the number of coding letters could be substantially increased (over fifty letters) if needed. The second advantage is the foundation for fast allele comparison if massively parallel sequencing is applied^36^.

### Null alleles

The occurence of null alleles complicates population genetic, parentage or forensic analyses as they can bias the estimation of various population parameters or cause the exclusion of true parents and misidentification in forensic cases. Generally, there are several ways to solve the problem with null alleles during the marker development. One of them is locus exclusion, as we did with OPTRI0432b. Another option is the addition of an extra F primer specific to the null allele for loci OPPEN22 and OPTRI1592, although a primer shift for OPTRI1592 was also considered. Several factors discouraged us from this solution, such as the required resequencing of all alleles, optimization and whole assay redesign and mainly the lack of appropriate positions to place new primer(s) due to high sequence similarity with other loci. Fortunately, it was possible to design a new F primer leading to the same length of the flanking region as for wild-type alleles. Therefore, the size changes should only be caused by changes in the number of repeats, as verified by comparison the sequencing and CE results. Furthermore, we decided to keep OPPEN38 as part of the MTP3 assay despite the occurrence of null allele and unsuccessful trials to characterize it. This seems to be a rather minor issue because of its occurrence in only 2 out of 36 tested cultivars, and we believe that it is more valuable to have another well-characterized marker given the current lack of such markers. The size overlap between OPTET119 and OPPEN09 was solved by changing the fluorophore for the marker OPPEN09.

### Internal validation

Internal validation makes it possible to objectively evaluate the performance of the OpiumPlex assays from many points of view including species specificity, balance characteristics, sensitivity, stutter effect, precision and accuracy, and represents the first important step towards a universally accepted tool for analysis of opium poppy DNA.

Testing on seven animal and nine plant species confirmed the specificity of the developed assays. The amplification of some markers within other species of the *Papaver* genus was not surprising, because these species share the same evolutionary history to some degree^55^, and one of the tested species, *P. glaucum*, is also considered a candidate species that contributes to the origin of *P. somniferum* as a species^55^. Moreover, marker cross-amplification within a group of closely related species is a common phenomenon^37,38^ and not a serious problem in most cases.

The OpiumPlex system seems to be relatively well balanced according to the metrics used. IELB data showed that the higher risk of signal loss could only occur for two markers (OPTET156 and OPTET105), with roughly half the signal strength of the others. The opposite problem, off-scale signal, could cause marker OPTET025d to have double the signal strength on average. This problem was caused by differences in the spectral calibration of the ABI 310 and ABI 3500 machines, and we solved it by making changes in the concentration of primers. This observation led us to the conclusion that a short optimization step might be necessary when researchers use other capillary machines. All but one peak height in the four dye channels met the recommended criterion of 50% or more^56^ for intracolour balance (ICB). Only the FAM-dye channel fell slightly under the borderline (0.49) in the MTP2 assay, which is probably connected with the generally weaker signal of OPTET105. For the last balance metric, heterozygote balance, we applied both routinely used methods, because each of them has its own advantages. The Hb method provides information about the directionality of preferential amplification, but is less robust against influential points, unlike the Hb′ method, which is also easier to interpret^41^. Our data showed that the typical threshold for heterozygote balance, set to 0.6^39,57^ or even 0.7 by some authors^20,37^, was exceeded for all loci on average. The newly developed markers seem to be more stable than the markers tested in the study of Young et al.^20^, who reported a common drop in the Hb′ ratio below the 0.7 threshold, but the presence of several outliers reminds us of the potential occurrence of suboptimal heterozygote balance in multiplexed assays. Only 4 out of 27 markers slightly favoured the amplification of high molecular weight (HMW) alleles over low molecular weight (LMW) alleles. Therefore, amplification bias towards the LMW allele is a more likely scenario. A typical example could be the most extreme Hb outlier in the locus OPPEN41, with a ratio value of 0.33, probably caused by the largest size difference between the alleles (37 bp) found among our samples.

According to the results, the optimal amount of DNA is within the range of 0.625–1.25 ng per reaction, which is equivalent to the nuclear content of 107–213 diploid cells based on poppy genome size estimation^14^. Our data are in accordance with an earlier study of opium poppy using different multiplexed STR assays^20^ and correspond to commonly reported sensitivities in other nonhuman organisms if genome size is taken into account^37,58^. Samples with less than 0.625 ng DNA suffered from an elevated error rate, and we can expect a low amount of extracted DNA from complex matrices such as heroin. Therefore, protocols developed for the analysis of low-copy DNA^59,60^ and/or more intense assay optimization would be necessary to increase the chances of retrieving full profiles with a suboptimal quantity of DNA.

The occurrence and dynamics of PCR artefacts known as stutter products are another important attribute of STR markers. We noticed two anomalies during the stutter ratio analysis. The first anomaly was a higher stutter ratio than expected for some tetra- and pentanucleotide microsatellites. Obviously, this observations did not fit the general rule where increasing the length of the motif leads to a lower stutter effect. Of course, other circumstances, such as the whole repeat structure^61^, number of repeat units^42^ or prevalence of AT pairs within the repetition^42^, can substantially influence the stutter profile of a given locus. The second phenomenon was the complex stutter pattern of the OPPEN09 and OPPEN22 loci comprising a series of n − 1 consecutive stutters. Although such a stutter profile is generally undesirable due to the incorporation of other noisy peaks in the system, it does not represent a serious problem, because the stutter values were still within the setup of the typical stutter filter (10–20%)^62^, even for the 99^th^ percentile, which is also true for the remaining markers. Detailed analysis of the allele-dependent stutter pattern confirmed that most of the alleles follow the expected trend where an increased number of repeats correspond to a higher stutter ratio. The noncanonical behaviour of the several remaining alleles is difficult to explain without further research. One possibility is the lack of a sufficient amount of measurements, as some alleles were rare. The other is a different repeat composition compared to “typical” alleles within the locus. The behaviour of these alleles would probably be better explained by the idea of the longest uninterrupted sequence (LUS)^42,61^. Moreover, the complexity of the problem could be increased thanks to the presence of isoalleles. We noticed at least one case within the locus OPPEN22, where 3 variants of allele 8 (size 208 bp) were detected. Furthermore, the pool of these isoalleles probably leads to an interesting stutter pattern with two groups of measurements divided by an apparent gap (Supplementary Fig. S12). The same pattern was observed not only for allele 8, but also for alleles 7 (203 bp) and 9 (213 bp) of the same locus, which might suggest the existence of other isoalleles. This hypothesis could be proven or disproven in the future when more sequenced genotypes become available.

### Descriptive statistics and population study

The average number of alleles per locus in this study is substantially higher than the variability in any study dealing with poppy STR reported to date^15–17,19,20^ with the exception of our previous research, where we found a similar variability for 17 genic STR markers^18^. The reason could be the different strategy of analysis and more stringent criteria for accepting STR locus as a marker compared to other studies (for details see discussion in the article Vašek et al.^18^). The observed heterozygosity was very low, because we were working with pure breeding lines and each registered variety has to met the DUS requirements (Distinctness, Uniformity and Stability, respectively^63^) typically reached through several generations of selfing. Only this population descriptive parameter was calculated, since our samples did not meet even loosely defined criteria to be classified as a population in the framework of population genetic theory. Therefore, calculating of typical population or forensic parameters together with testing Hardy–Weinberg and linkage equilibrium did not make sense. The only relevant parameter is the capability to discriminate varieties, which was the reason why we made pairwise comparisons of each sample. We considered one variety to be different from another only if all individual plants of a given variety were different. The reasoning behind this stem from the simple fact that any DNA profiling system needs to address two levels of variability—variability within and between varieties. The variety of sexually propagated plants, unlike clones, cannot be defined by only one genotype, even for (highly inbred) pure breeding lines. There is always variability to some degree, because variety registration is made according to phenotype, not genotype, and the purity of the breeding line depends on many factors, such as breeding process (e.g., how many generations of selfing were made), quality of maintenance breeding or degree of certified seeds (C1 vs E). One can argue that our number of samples per variety (typically 4–5) was still small, but we also have results from the variety standards of poppy. These VSP helped us to determine wheter we covered most of the intracultivar variability, and no new alleles were found for any VSP.

The pairwise comparison showed that the OpiumPlex system was unable to discriminate several varieties from each other and these varieties consist of two groups. One group comprises four varieties (Sokol, Racek, Orel and Akvarel), and the second group comprises two other varieties (Postomi and Buddha). Unfortunately, information about the breeding history of the above varieties is scarce. The first three varieties within the former group have several things in common—breeder, white colour of seeds and starting breeding material^64,65^. Each variety was bred through repeated individual selection^64,65^. Therefore, we can speculate that the genetic differences between these varieties are very subtle. We have almost no information about the Akvarel variety, but it has a different breeder and colour of seeds (ochre). The latter group consists of two pharmaceutical varieties with high morphine content and the same country of origin, but no other information is available.

PCA and CLU analysis help us decipher the similarities and genetic structure of the studied accessions. One thing we were curious about was whether the varieties would be clustered according to recognized categories (culinary, dual, pharmaceutical, ornamental). The delimited categories roughly reflect morphine content, but the boundary seems to be relatively gradual for dual/culinary varieties and sharper for pharmaceutical varieties vs other types. It is interesting that markers scattered throughout the whole genome (Supplementary Table S13, Fig. 2) follow this categorization, although they do not directly correlate with the genes of the morphine pathway, as they are probably located either on chromosomes 1, 2 and 7^14^ or 1, 3, 4 and 8^66^, depending on the study. This might be the consequence of long-term selection, which shapes the whole genome together with the utilization of different plant reservoirs for breeding high/low content varieties or breeding for other characteristics (sensory or aesthetic). The most notable exception is the pharmaceutical variety Lina, which is more similar to dual than pharmaceutical varieties (Fig. 4). On the other hand, Lina is a high noscapine variety, unlike the others. Therefore, it is possible that its morphine content corresponds more closely to dual varieties (information is not available). Similarly, Lazur is labeled as a pharmaceutical variety, but its morphine content varies between dual and pharmaceutical varieties (Table 1). Further, CLU analysis confirmed that classification according to country of registration is less important than classification according to categories of purpose. This result is in congruence with our knowledge about the breeding practices where the breeder can use a whole repertoire of available cultivars registered in the European catalogue, local varieties or gene resources from genebanks. It is also in agreement with the politics of the given country, because in the Czech or Slovak Republic *P. somniferum* is grown for seeds (breeding for culinary or dual varieties) whereas in Hungary it is bred mostly for secondary metabolites (breeding for pharmaceutical or dual varieties).

In conclusion, empirical evaluation of the system showed that there is room for improvement, and other tests (e.g., interlaboratory comparison or analysis of real casework samples) would be necessary to reach its full potential. Nonetheless, the introduced assays represent the first important step towards a universal genotyping system that provides a practical new tool for the individualization of opium poppy, raw opium or heroin samples. Therefore, we hope that the scientific community finds our OpiumPlex system useful and worthy of further development.

## Supplementary Information

Supplementary Information 1.

Supplementary Information 2.

Supplementary Information 3.

Supplementary Information 4.

Supplementary Information 5.

## Data Availability

Sequencing data were deposited in the GenBank repository (accession numbers MW364129—MW364241). All STR whole genome analysis, raw capillary and graphs input data are available upon reasonable request from the corresponding author (J.V.). There are no restrictions on data availability.
