# Supplementary material for: OpiumPlex is a novel microsatellite system for profiling opium poppy (Papaver somniferum L.)
Source: Sci Rep. 2021 Jun 17;11:12799. doi: 10.1038/s41598-021-91962-1 (PMC8211840; doi:10.1038/s41598-021-91962-1)
Supplement: Supplementary file 1 — Supplementary Information 1. [file 41598_2021_91962_MOESM1_ESM.pptx]

## Slide 1
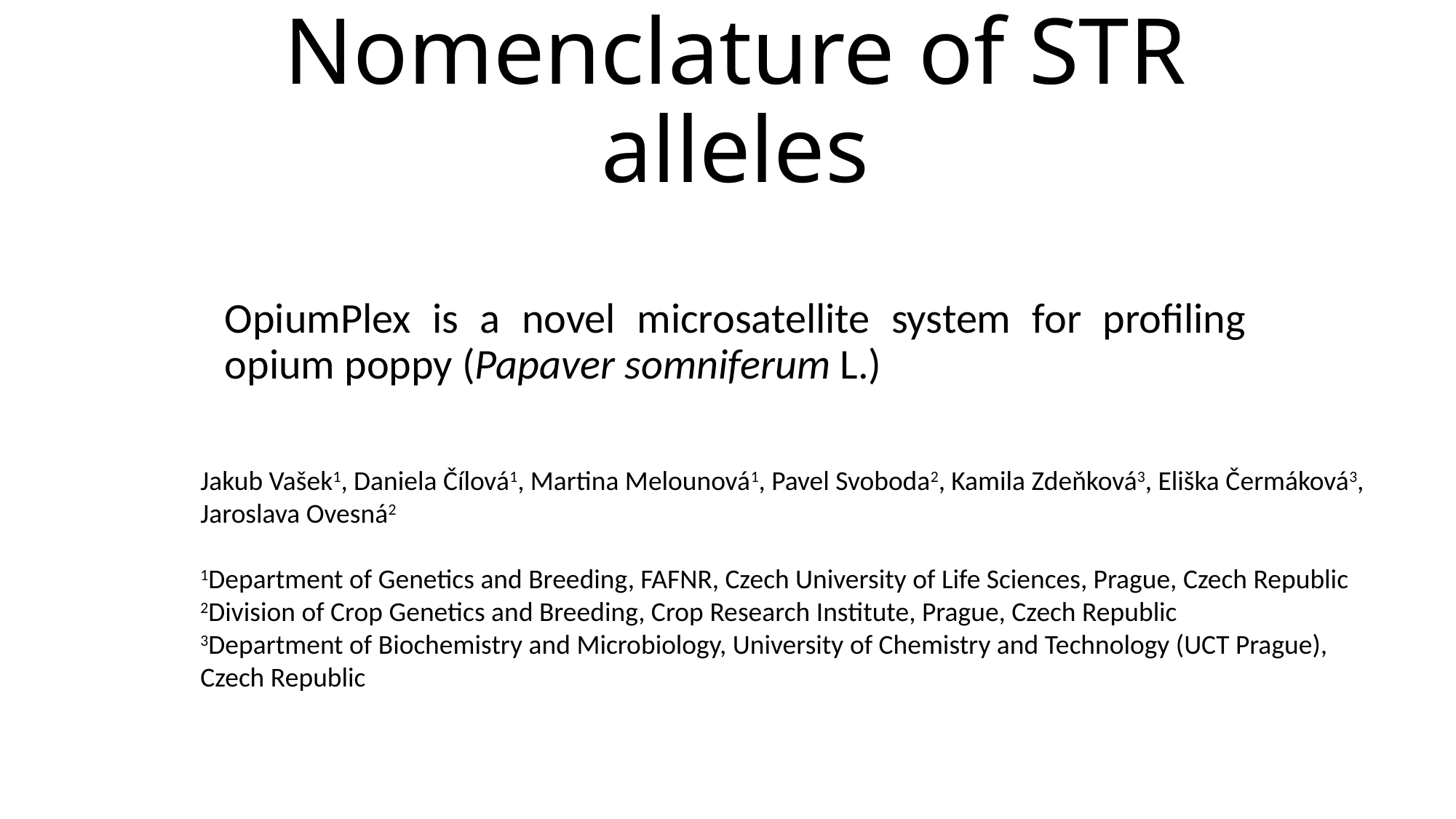

# Nomenclature of STR alleles
OpiumPlex is a novel microsatellite system for profiling opium poppy (Papaver somniferum L.)
Jakub Vašek1, Daniela Čílová1, Martina Melounová1, Pavel Svoboda2, Kamila Zdeňková3, Eliška Čermáková3, Jaroslava Ovesná2
1Department of Genetics and Breeding, FAFNR, Czech University of Life Sciences, Prague, Czech Republic
2Division of Crop Genetics and Breeding, Crop Research Institute, Prague, Czech Republic
3Department of Biochemistry and Microbiology, University of Chemistry and Technology (UCT Prague), Czech Republic

## Slide 2
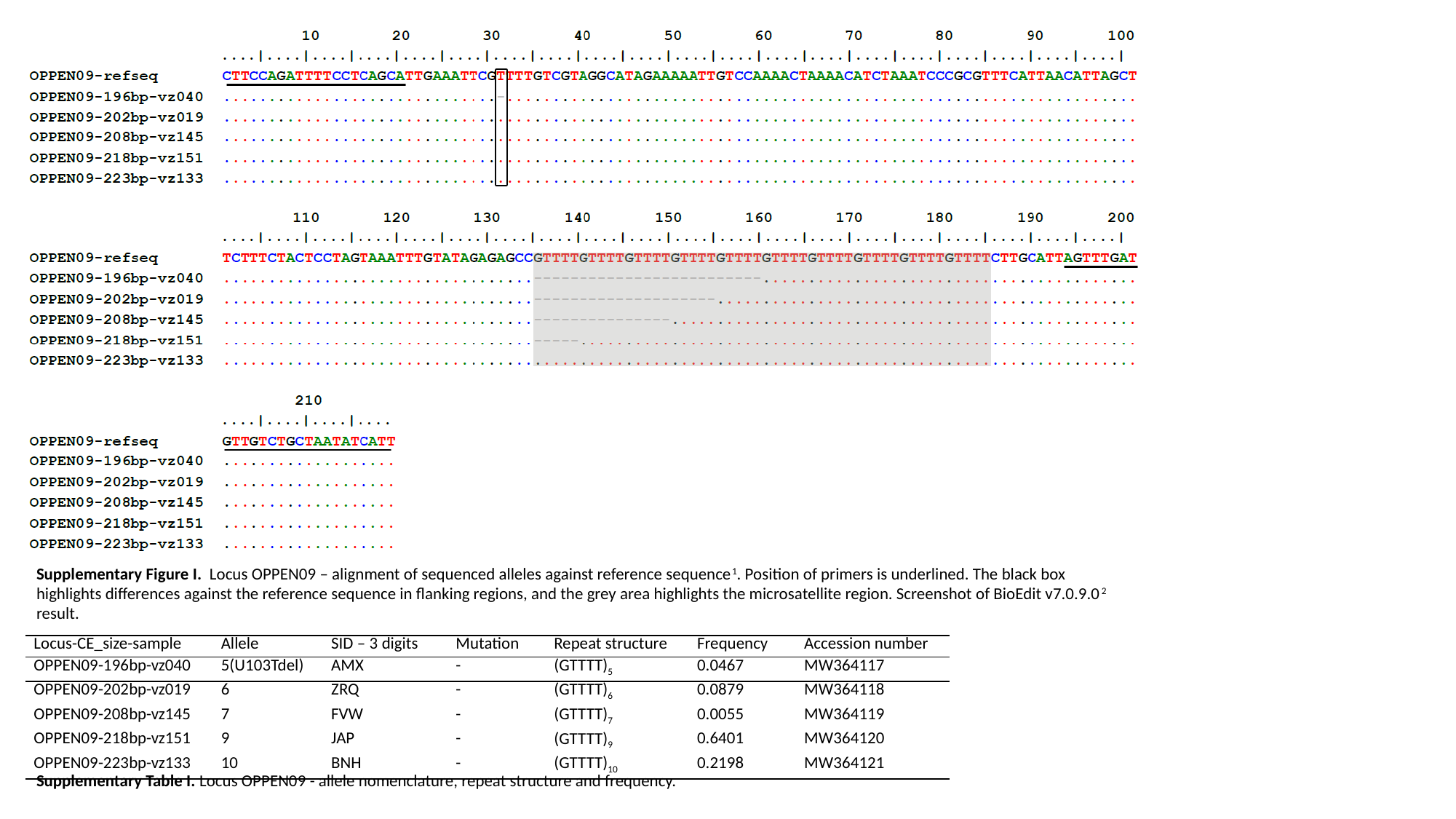

Supplementary Figure I. Locus OPPEN09 – alignment of sequenced alleles against reference sequence1. Position of primers is underlined. The black box highlights differences against the reference sequence in flanking regions, and the grey area highlights the microsatellite region. Screenshot of BioEdit v7.0.9.02 result.
| Locus-CE\_size-sample | Allele | SID – 3 digits | Mutation | Repeat structure | Frequency | Accession number |
| --- | --- | --- | --- | --- | --- | --- |
| OPPEN09-196bp-vz040 | 5(U103Tdel) | AMX | - | (GTTTT)5 | 0.0467 | MW364117 |
| OPPEN09-202bp-vz019 | 6 | ZRQ | - | (GTTTT)6 | 0.0879 | MW364118 |
| OPPEN09-208bp-vz145 | 7 | FVW | - | (GTTTT)7 | 0.0055 | MW364119 |
| OPPEN09-218bp-vz151 | 9 | JAP | - | (GTTTT)9 | 0.6401 | MW364120 |
| OPPEN09-223bp-vz133 | 10 | BNH | - | (GTTTT)10 | 0.2198 | MW364121 |
Supplementary Table I. Locus OPPEN09 - allele nomenclature, repeat structure and frequency.

## Slide 3
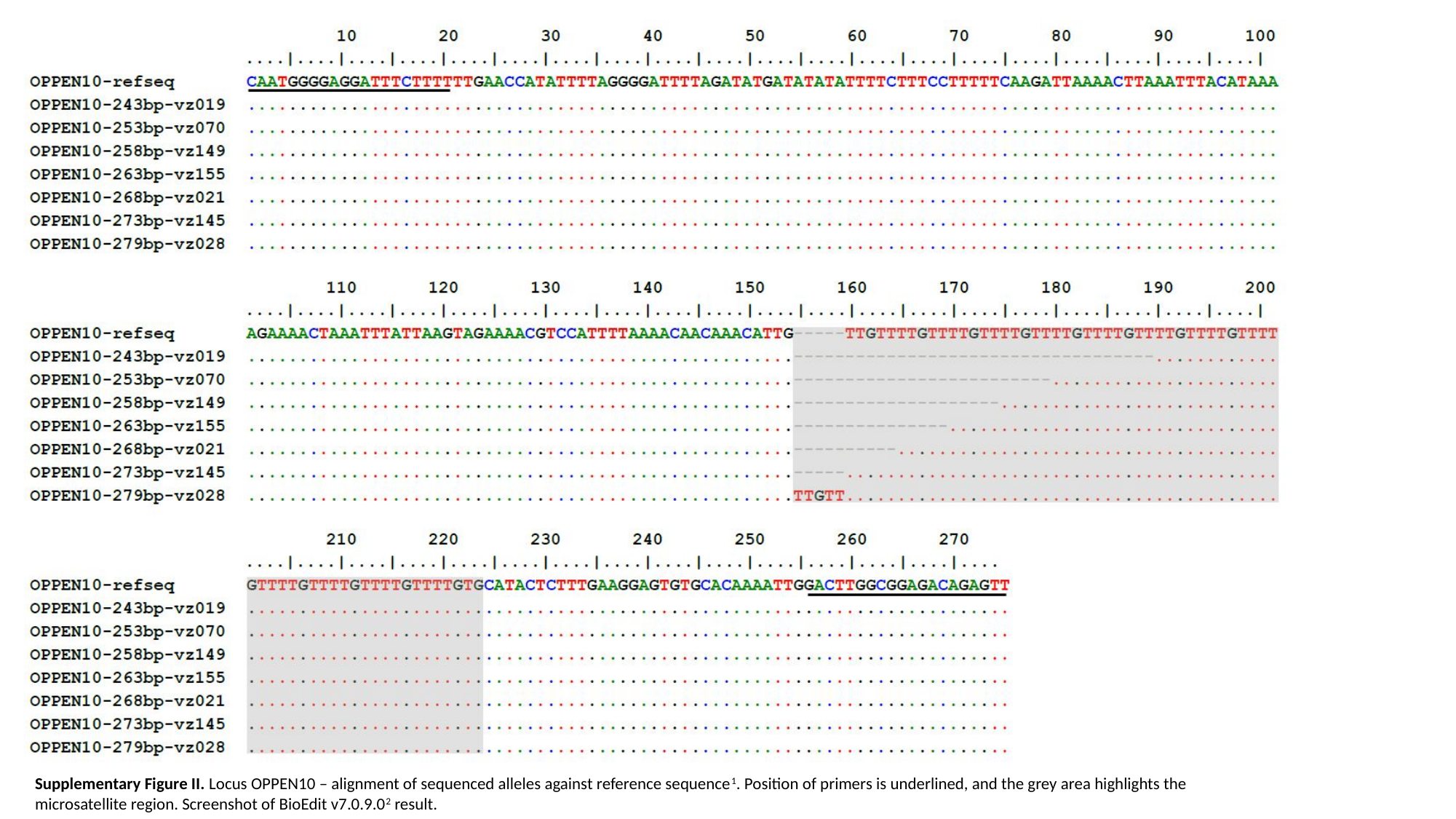

Supplementary Figure II. Locus OPPEN10 – alignment of sequenced alleles against reference sequence1. Position of primers is underlined, and the grey area highlights the microsatellite region. Screenshot of BioEdit v7.0.9.02 result.

## Slide 4
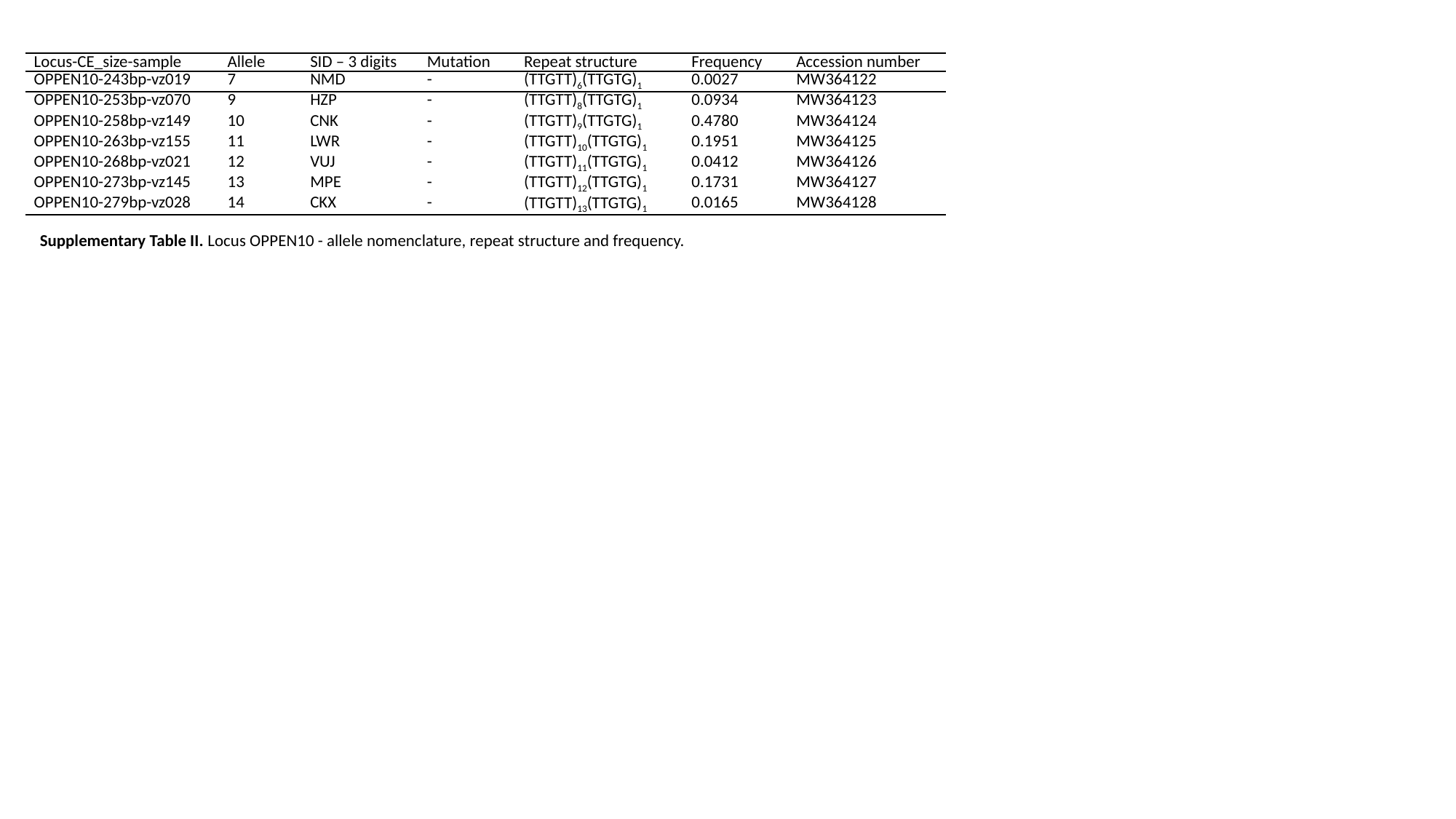

| Locus-CE\_size-sample | Allele | SID – 3 digits | Mutation | Repeat structure | Frequency | Accession number |
| --- | --- | --- | --- | --- | --- | --- |
| OPPEN10-243bp-vz019 | 7 | NMD | - | (TTGTT)6(TTGTG)1 | 0.0027 | MW364122 |
| OPPEN10-253bp-vz070 | 9 | HZP | - | (TTGTT)8(TTGTG)1 | 0.0934 | MW364123 |
| OPPEN10-258bp-vz149 | 10 | CNK | - | (TTGTT)9(TTGTG)1 | 0.4780 | MW364124 |
| OPPEN10-263bp-vz155 | 11 | LWR | - | (TTGTT)10(TTGTG)1 | 0.1951 | MW364125 |
| OPPEN10-268bp-vz021 | 12 | VUJ | - | (TTGTT)11(TTGTG)1 | 0.0412 | MW364126 |
| OPPEN10-273bp-vz145 | 13 | MPE | - | (TTGTT)12(TTGTG)1 | 0.1731 | MW364127 |
| OPPEN10-279bp-vz028 | 14 | CKX | - | (TTGTT)13(TTGTG)1 | 0.0165 | MW364128 |
Supplementary Table II. Locus OPPEN10 - allele nomenclature, repeat structure and frequency.

## Slide 5
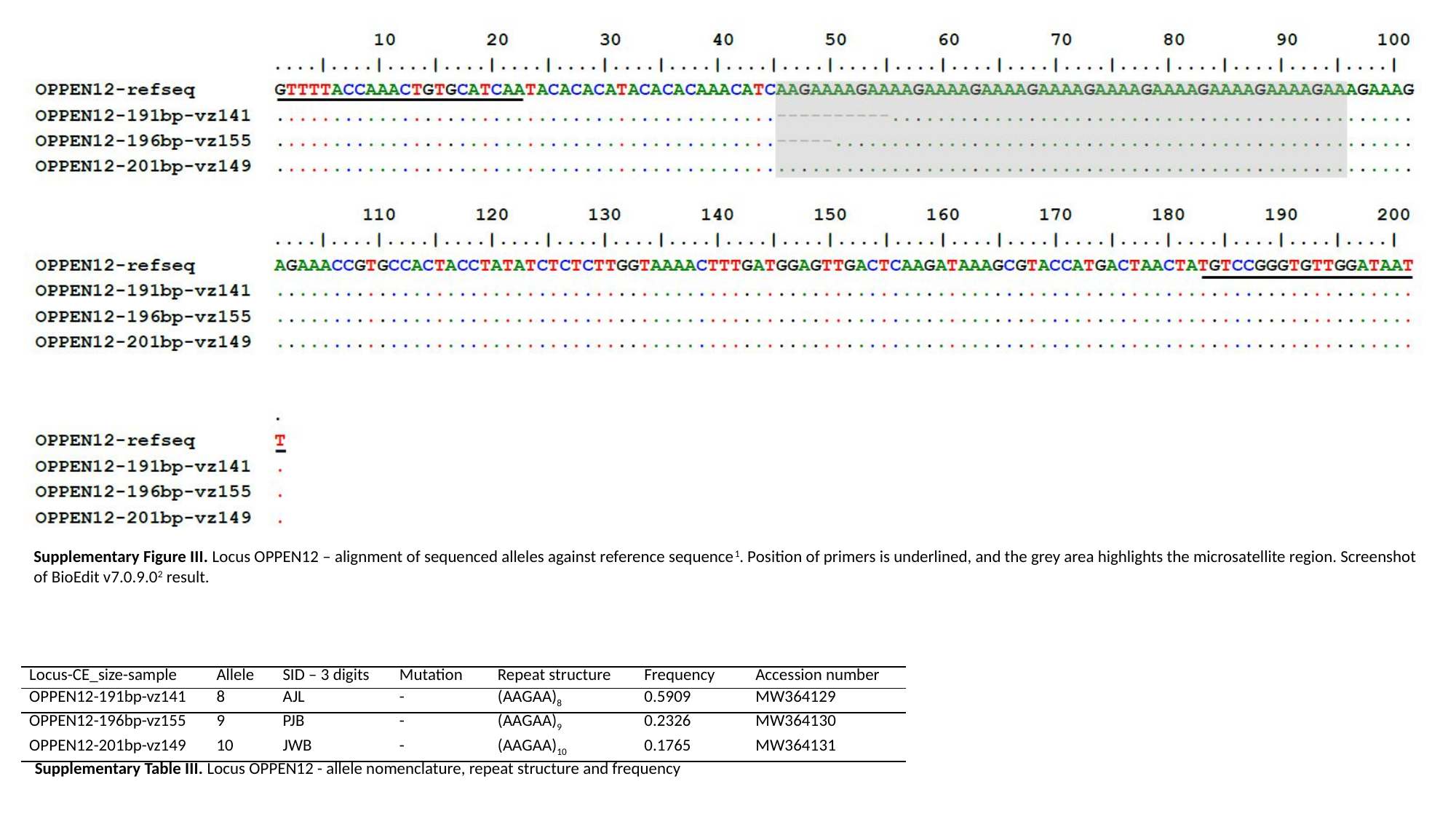

Supplementary Figure III. Locus OPPEN12 – alignment of sequenced alleles against reference sequence1. Position of primers is underlined, and the grey area highlights the microsatellite region. Screenshot of BioEdit v7.0.9.02 result.
| Locus-CE\_size-sample | Allele | SID – 3 digits | Mutation | Repeat structure | Frequency | Accession number |
| --- | --- | --- | --- | --- | --- | --- |
| OPPEN12-191bp-vz141 | 8 | AJL | - | (AAGAA)8 | 0.5909 | MW364129 |
| OPPEN12-196bp-vz155 | 9 | PJB | - | (AAGAA)9 | 0.2326 | MW364130 |
| OPPEN12-201bp-vz149 | 10 | JWB | - | (AAGAA)10 | 0.1765 | MW364131 |
Supplementary Table III. Locus OPPEN12 - allele nomenclature, repeat structure and frequency

## Slide 6
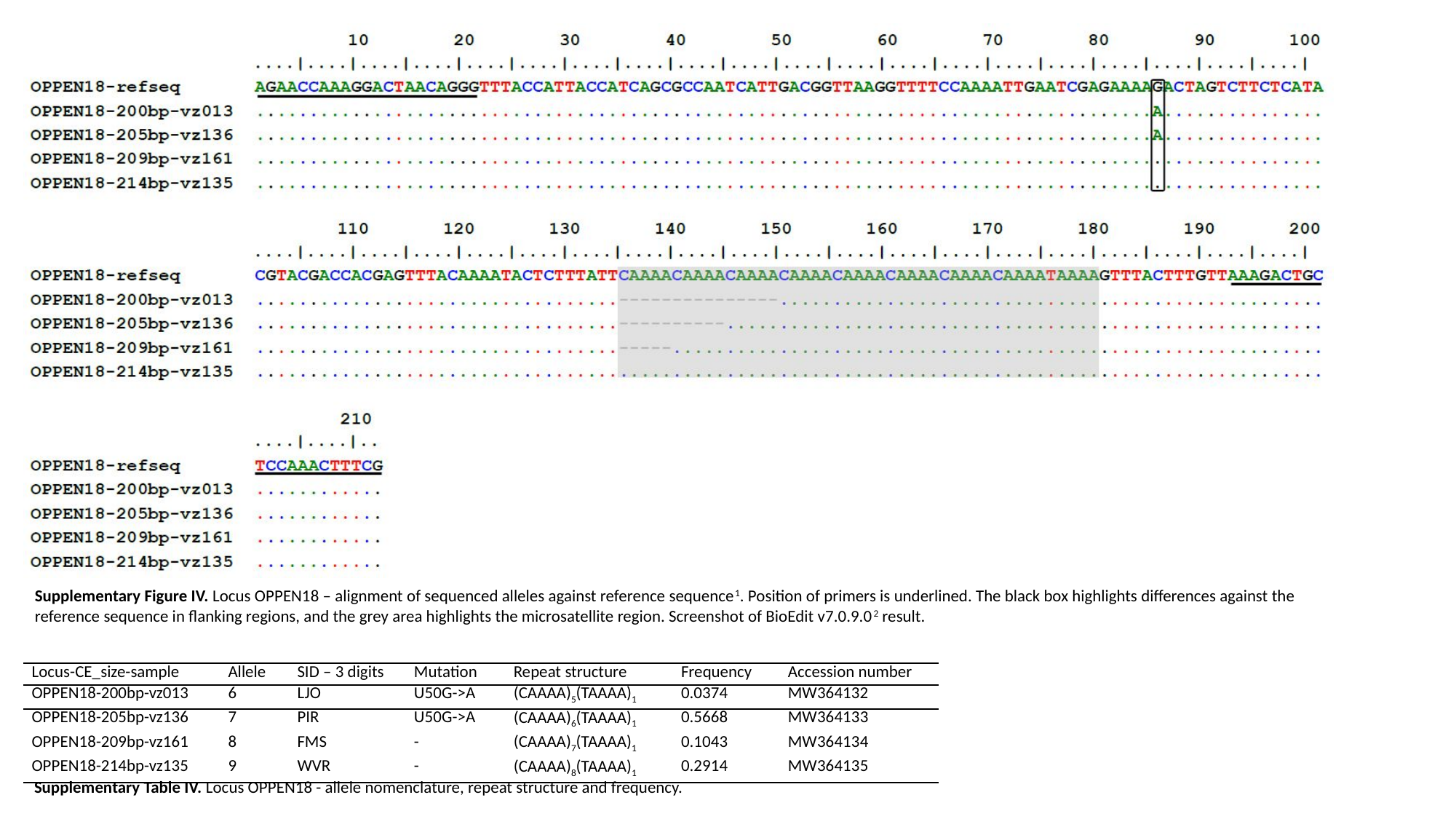

Supplementary Figure IV. Locus OPPEN18 – alignment of sequenced alleles against reference sequence1. Position of primers is underlined. The black box highlights differences against the reference sequence in flanking regions, and the grey area highlights the microsatellite region. Screenshot of BioEdit v7.0.9.02 result.
| Locus-CE\_size-sample | Allele | SID – 3 digits | Mutation | Repeat structure | Frequency | Accession number |
| --- | --- | --- | --- | --- | --- | --- |
| OPPEN18-200bp-vz013 | 6 | LJO | U50G->A | (CAAAA)5(TAAAA)1 | 0.0374 | MW364132 |
| OPPEN18-205bp-vz136 | 7 | PIR | U50G->A | (CAAAA)6(TAAAA)1 | 0.5668 | MW364133 |
| OPPEN18-209bp-vz161 | 8 | FMS | - | (CAAAA)7(TAAAA)1 | 0.1043 | MW364134 |
| OPPEN18-214bp-vz135 | 9 | WVR | - | (CAAAA)8(TAAAA)1 | 0.2914 | MW364135 |
Supplementary Table IV. Locus OPPEN18 - allele nomenclature, repeat structure and frequency.

## Slide 7
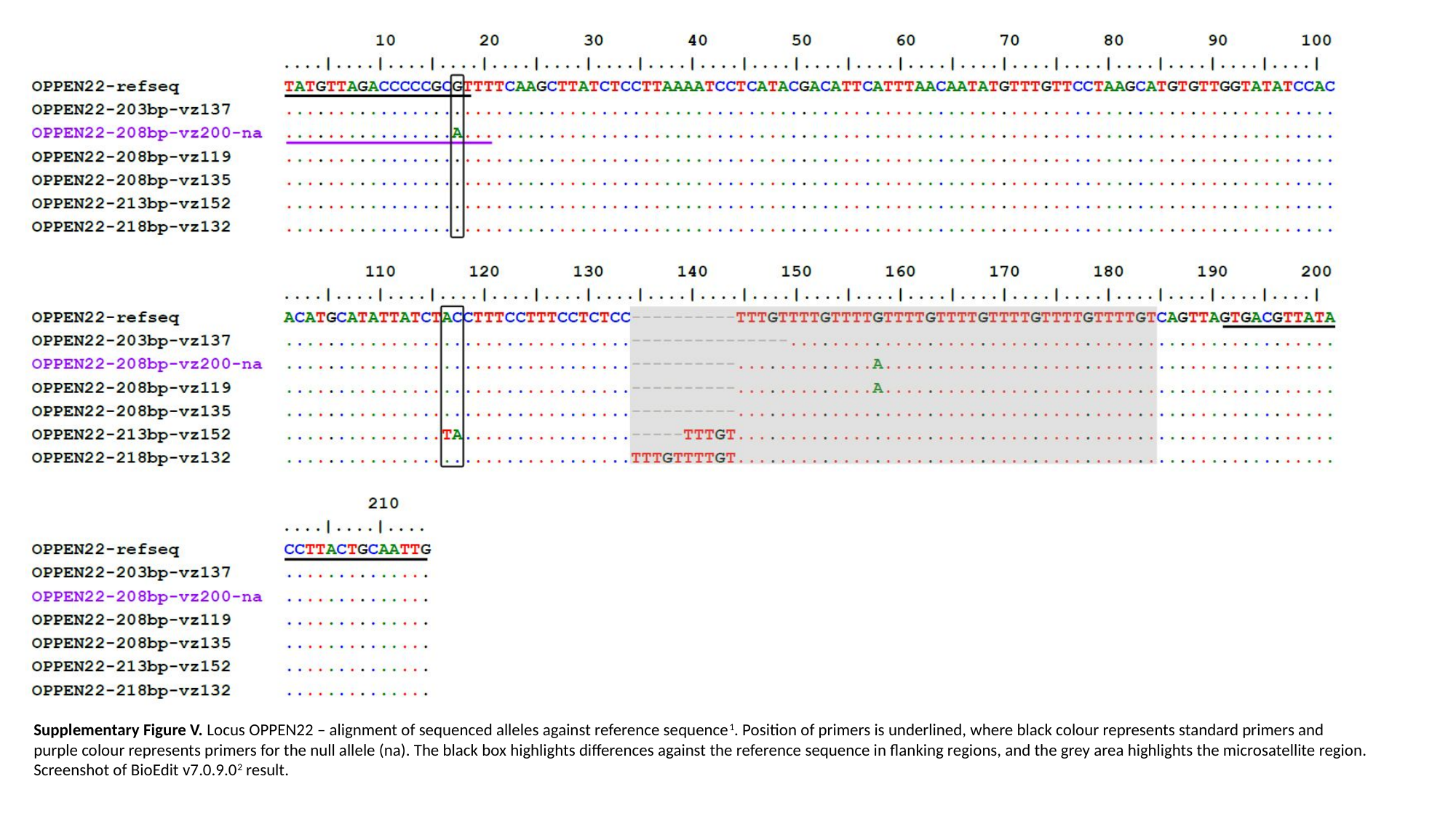

Supplementary Figure V. Locus OPPEN22 – alignment of sequenced alleles against reference sequence1. Position of primers is underlined, where black colour represents standard primers and purple colour represents primers for the null allele (na). The black box highlights differences against the reference sequence in flanking regions, and the grey area highlights the microsatellite region. Screenshot of BioEdit v7.0.9.02 result.

## Slide 8
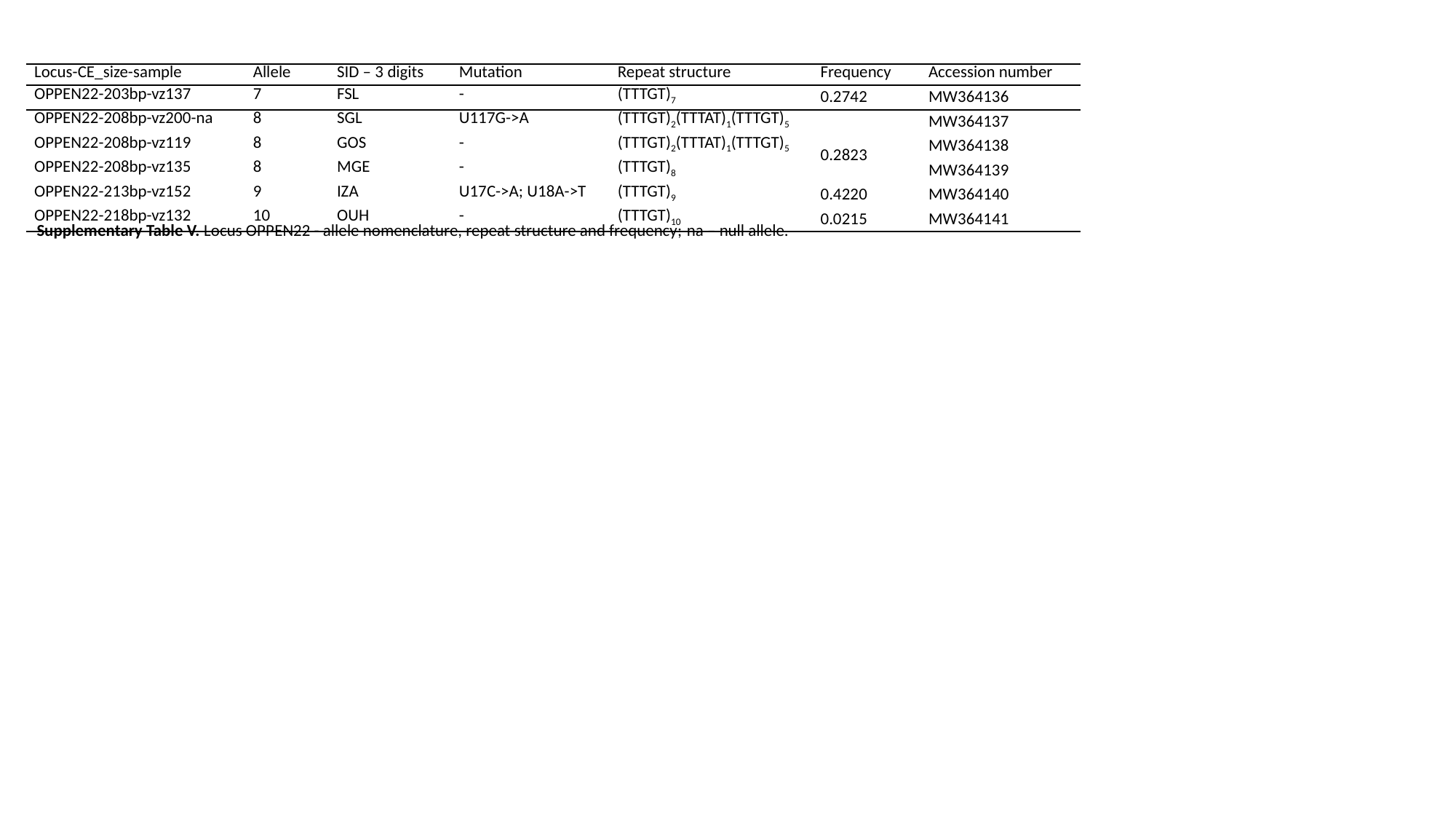

| Locus-CE\_size-sample | Allele | SID – 3 digits | Mutation | Repeat structure | Frequency | Accession number |
| --- | --- | --- | --- | --- | --- | --- |
| OPPEN22-203bp-vz137 | 7 | FSL | - | (TTTGT)7 | 0.2742 | MW364136 |
| OPPEN22-208bp-vz200-na | 8 | SGL | U117G->A | (TTTGT)2(TTTAT)1(TTTGT)5 | 0.2823 | MW364137 |
| OPPEN22-208bp-vz119 | 8 | GOS | - | (TTTGT)2(TTTAT)1(TTTGT)5 | | MW364138 |
| OPPEN22-208bp-vz135 | 8 | MGE | - | (TTTGT)8 | | MW364139 |
| OPPEN22-213bp-vz152 | 9 | IZA | U17C->A; U18A->T | (TTTGT)9 | 0.4220 | MW364140 |
| OPPEN22-218bp-vz132 | 10 | OUH | - | (TTTGT)10 | 0.0215 | MW364141 |
Supplementary Table V. Locus OPPEN22 - allele nomenclature, repeat structure and frequency; na – null allele.

## Slide 9
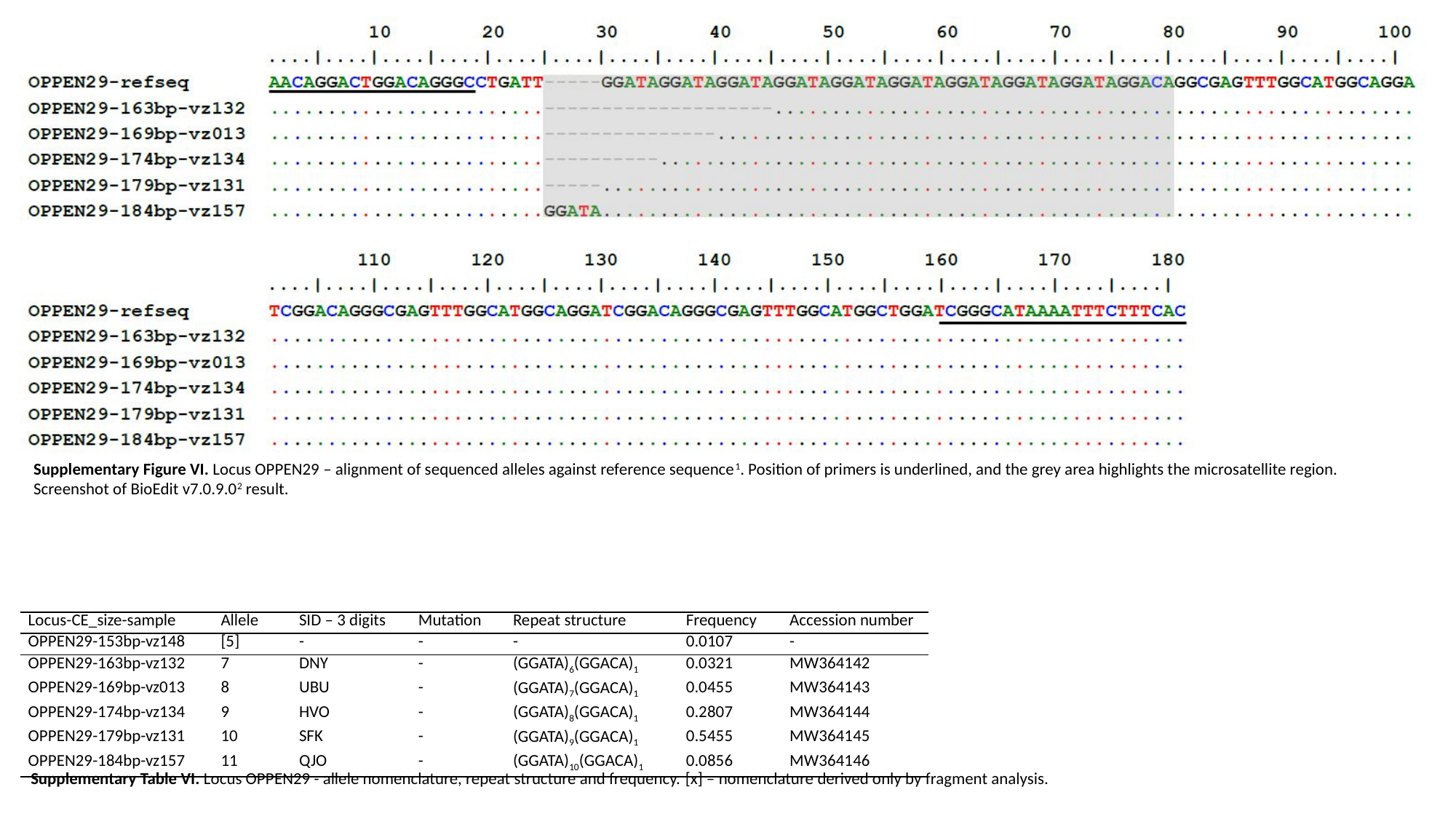

Supplementary Figure VI. Locus OPPEN29 – alignment of sequenced alleles against reference sequence1. Position of primers is underlined, and the grey area highlights the microsatellite region. Screenshot of BioEdit v7.0.9.02 result.
| Locus-CE\_size-sample | Allele | SID – 3 digits | Mutation | Repeat structure | Frequency | Accession number |
| --- | --- | --- | --- | --- | --- | --- |
| OPPEN29-153bp-vz148 | [5] | - | - | - | 0.0107 | - |
| OPPEN29-163bp-vz132 | 7 | DNY | - | (GGATA)6(GGACA)1 | 0.0321 | MW364142 |
| OPPEN29-169bp-vz013 | 8 | UBU | - | (GGATA)7(GGACA)1 | 0.0455 | MW364143 |
| OPPEN29-174bp-vz134 | 9 | HVO | - | (GGATA)8(GGACA)1 | 0.2807 | MW364144 |
| OPPEN29-179bp-vz131 | 10 | SFK | - | (GGATA)9(GGACA)1 | 0.5455 | MW364145 |
| OPPEN29-184bp-vz157 | 11 | QJO | - | (GGATA)10(GGACA)1 | 0.0856 | MW364146 |
Supplementary Table VI. Locus OPPEN29 - allele nomenclature, repeat structure and frequency. [x] – nomenclature derived only by fragment analysis.

## Slide 10
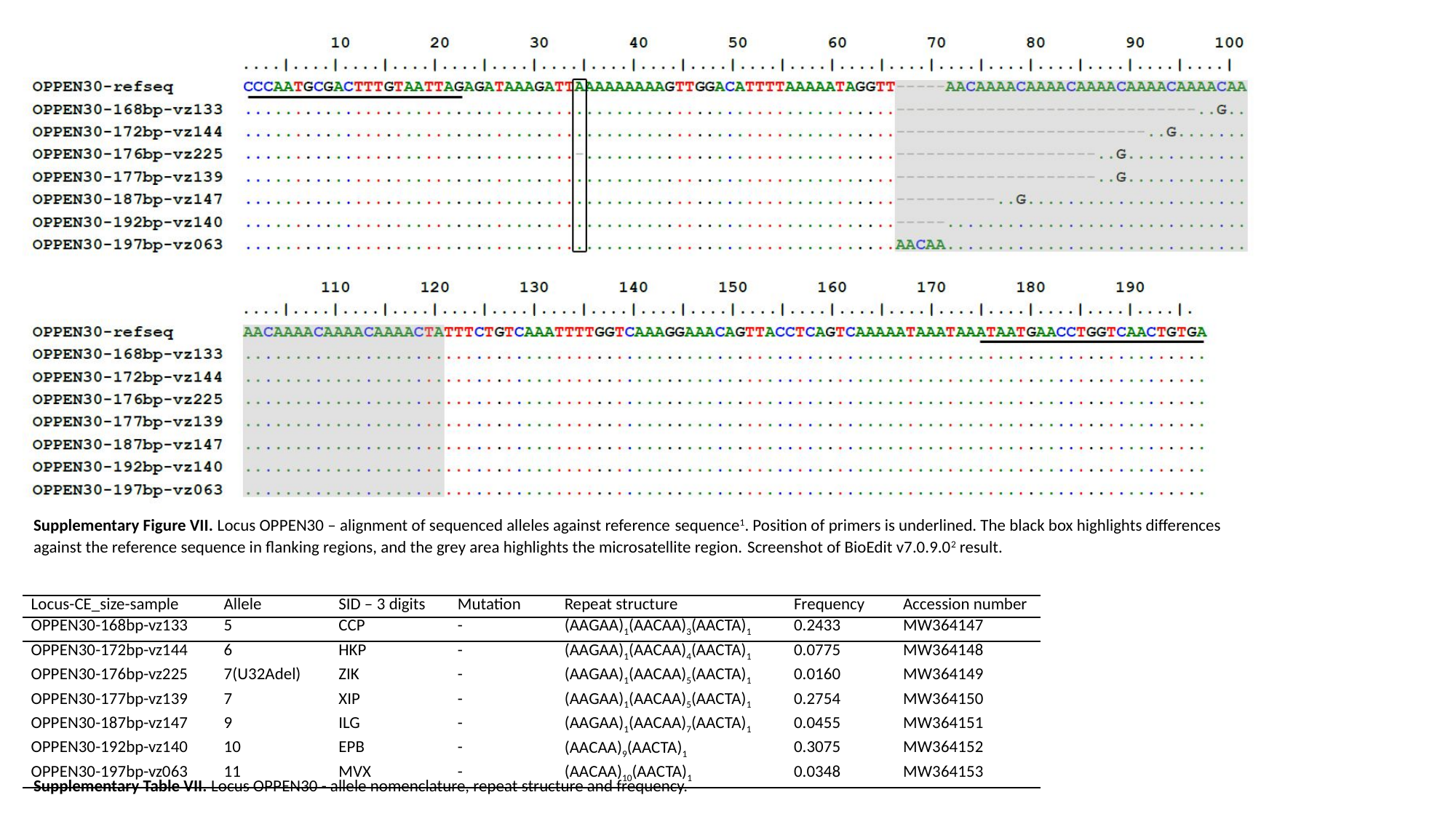

Supplementary Figure VII. Locus OPPEN30 – alignment of sequenced alleles against reference sequence1. Position of primers is underlined. The black box highlights differences against the reference sequence in flanking regions, and the grey area highlights the microsatellite region. Screenshot of BioEdit v7.0.9.02 result.
| Locus-CE\_size-sample | Allele | SID – 3 digits | Mutation | Repeat structure | Frequency | Accession number |
| --- | --- | --- | --- | --- | --- | --- |
| OPPEN30-168bp-vz133 | 5 | CCP | - | (AAGAA)1(AACAA)3(AACTA)1 | 0.2433 | MW364147 |
| OPPEN30-172bp-vz144 | 6 | HKP | - | (AAGAA)1(AACAA)4(AACTA)1 | 0.0775 | MW364148 |
| OPPEN30-176bp-vz225 | 7(U32Adel) | ZIK | - | (AAGAA)1(AACAA)5(AACTA)1 | 0.0160 | MW364149 |
| OPPEN30-177bp-vz139 | 7 | XIP | - | (AAGAA)1(AACAA)5(AACTA)1 | 0.2754 | MW364150 |
| OPPEN30-187bp-vz147 | 9 | ILG | - | (AAGAA)1(AACAA)7(AACTA)1 | 0.0455 | MW364151 |
| OPPEN30-192bp-vz140 | 10 | EPB | - | (AACAA)9(AACTA)1 | 0.3075 | MW364152 |
| OPPEN30-197bp-vz063 | 11 | MVX | - | (AACAA)10(AACTA)1 | 0.0348 | MW364153 |
Supplementary Table VII. Locus OPPEN30 - allele nomenclature, repeat structure and frequency.

## Slide 11
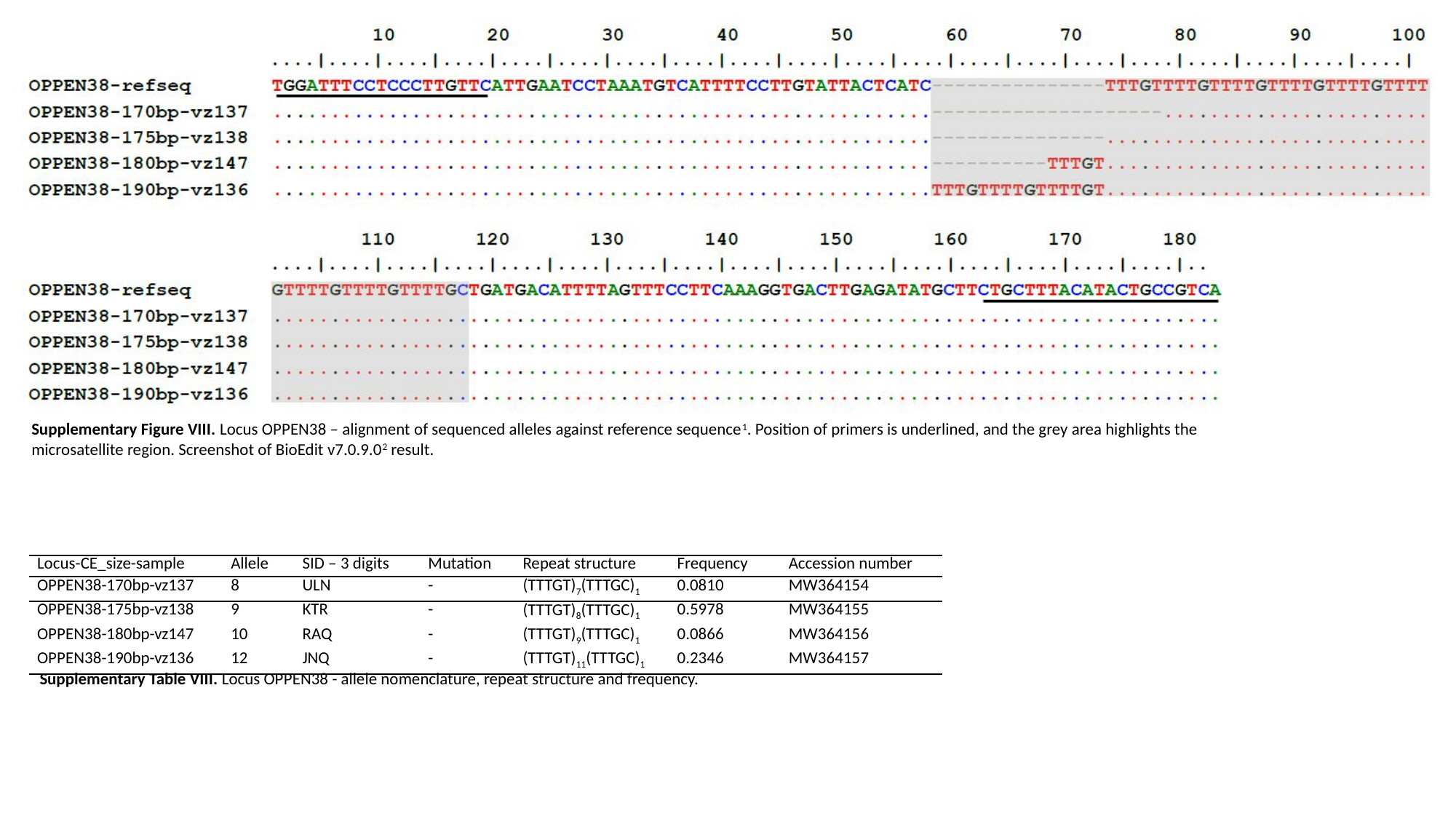

Supplementary Figure VIII. Locus OPPEN38 – alignment of sequenced alleles against reference sequence1. Position of primers is underlined, and the grey area highlights the microsatellite region. Screenshot of BioEdit v7.0.9.02 result.
| Locus-CE\_size-sample | Allele | SID – 3 digits | Mutation | Repeat structure | Frequency | Accession number |
| --- | --- | --- | --- | --- | --- | --- |
| OPPEN38-170bp-vz137 | 8 | ULN | - | (TTTGT)7(TTTGC)1 | 0.0810 | MW364154 |
| OPPEN38-175bp-vz138 | 9 | KTR | - | (TTTGT)8(TTTGC)1 | 0.5978 | MW364155 |
| OPPEN38-180bp-vz147 | 10 | RAQ | - | (TTTGT)9(TTTGC)1 | 0.0866 | MW364156 |
| OPPEN38-190bp-vz136 | 12 | JNQ | - | (TTTGT)11(TTTGC)1 | 0.2346 | MW364157 |
Supplementary Table VIII. Locus OPPEN38 - allele nomenclature, repeat structure and frequency.

## Slide 12
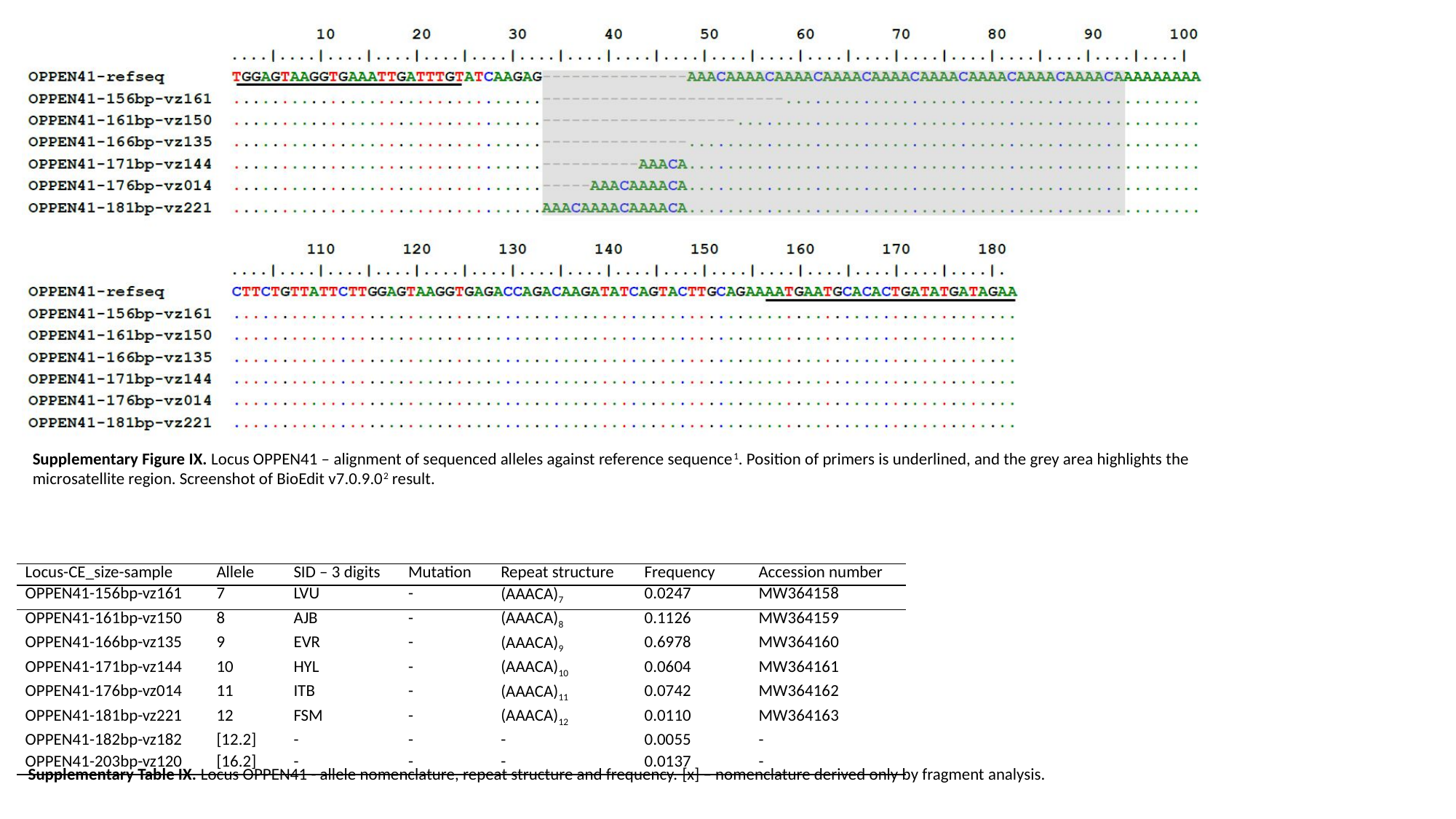

Supplementary Figure IX. Locus OPPEN41 – alignment of sequenced alleles against reference sequence1. Position of primers is underlined, and the grey area highlights the microsatellite region. Screenshot of BioEdit v7.0.9.02 result.
| Locus-CE\_size-sample | Allele | SID – 3 digits | Mutation | Repeat structure | Frequency | Accession number |
| --- | --- | --- | --- | --- | --- | --- |
| OPPEN41-156bp-vz161 | 7 | LVU | - | (AAACA)7 | 0.0247 | MW364158 |
| OPPEN41-161bp-vz150 | 8 | AJB | - | (AAACA)8 | 0.1126 | MW364159 |
| OPPEN41-166bp-vz135 | 9 | EVR | - | (AAACA)9 | 0.6978 | MW364160 |
| OPPEN41-171bp-vz144 | 10 | HYL | - | (AAACA)10 | 0.0604 | MW364161 |
| OPPEN41-176bp-vz014 | 11 | ITB | - | (AAACA)11 | 0.0742 | MW364162 |
| OPPEN41-181bp-vz221 | 12 | FSM | - | (AAACA)12 | 0.0110 | MW364163 |
| OPPEN41-182bp-vz182 | [12.2] | - | - | - | 0.0055 | - |
| OPPEN41-203bp-vz120 | [16.2] | - | - | - | 0.0137 | - |
Supplementary Table IX. Locus OPPEN41 - allele nomenclature, repeat structure and frequency. [x] – nomenclature derived only by fragment analysis.

## Slide 13
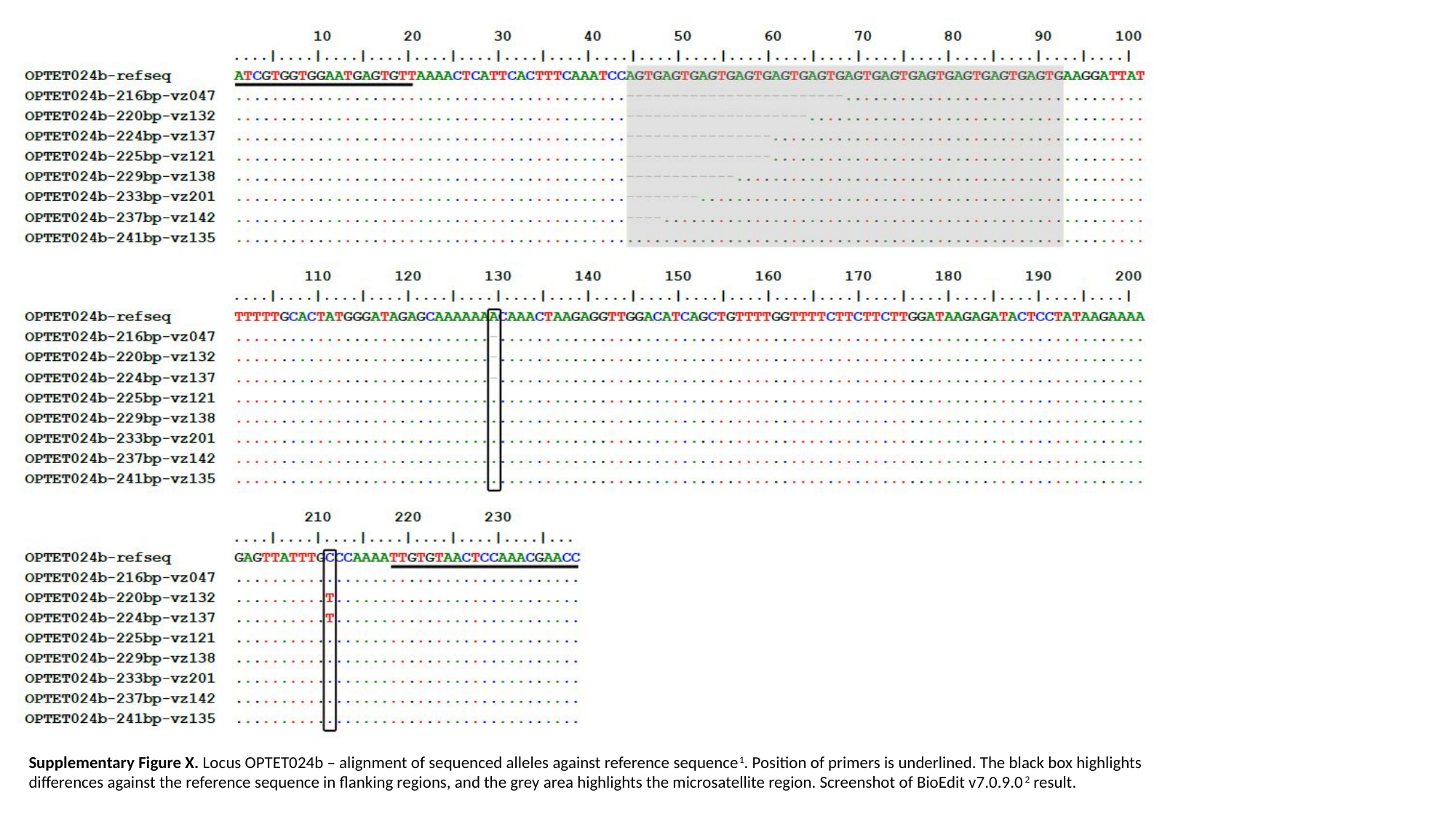

Supplementary Figure X. Locus OPTET024b – alignment of sequenced alleles against reference sequence1. Position of primers is underlined. The black box highlights differences against the reference sequence in flanking regions, and the grey area highlights the microsatellite region. Screenshot of BioEdit v7.0.9.02 result.

## Slide 14
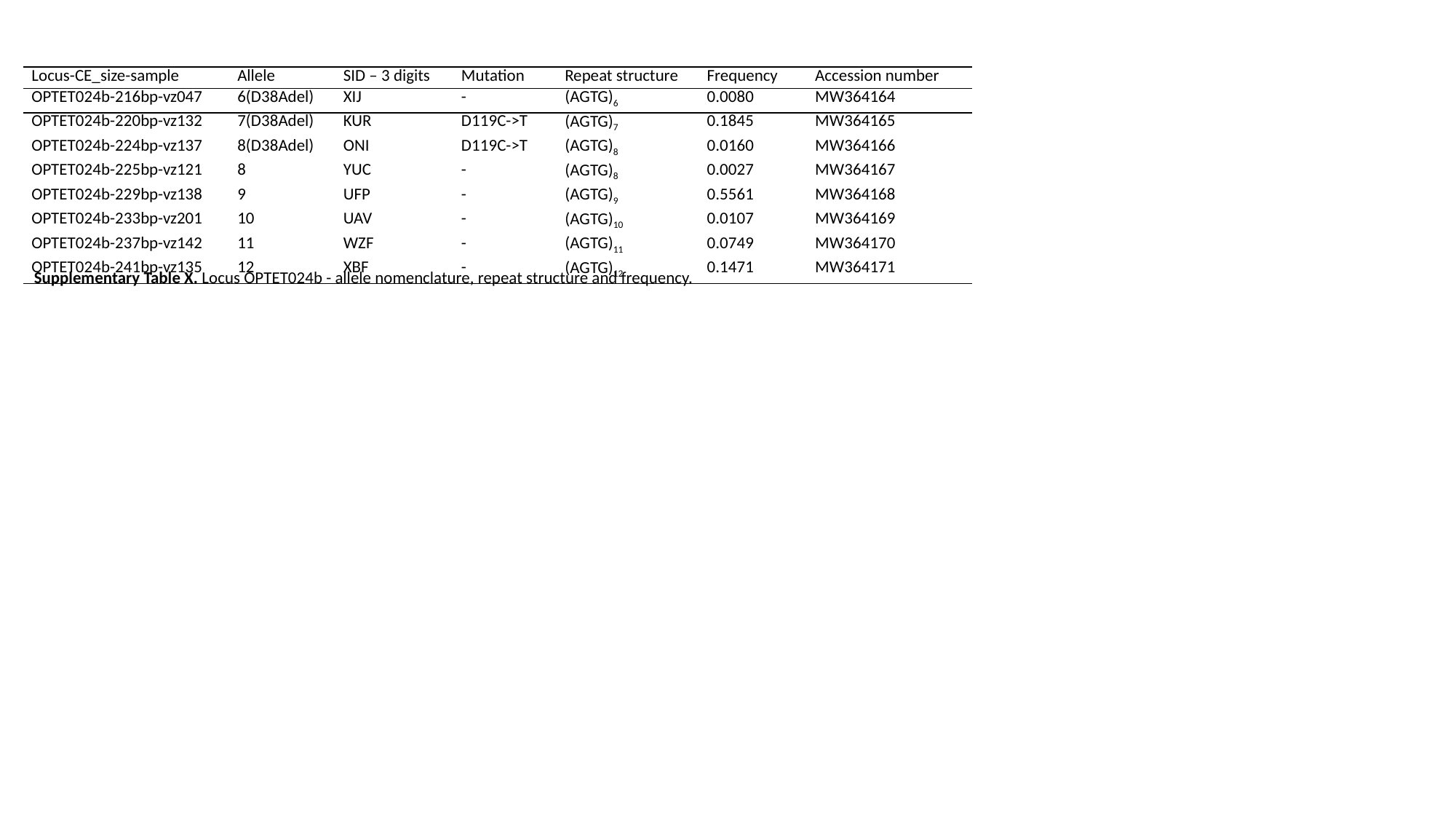

| Locus-CE\_size-sample | Allele | SID – 3 digits | Mutation | Repeat structure | Frequency | Accession number |
| --- | --- | --- | --- | --- | --- | --- |
| OPTET024b-216bp-vz047 | 6(D38Adel) | XIJ | - | (AGTG)6 | 0.0080 | MW364164 |
| OPTET024b-220bp-vz132 | 7(D38Adel) | KUR | D119C->T | (AGTG)7 | 0.1845 | MW364165 |
| OPTET024b-224bp-vz137 | 8(D38Adel) | ONI | D119C->T | (AGTG)8 | 0.0160 | MW364166 |
| OPTET024b-225bp-vz121 | 8 | YUC | - | (AGTG)8 | 0.0027 | MW364167 |
| OPTET024b-229bp-vz138 | 9 | UFP | - | (AGTG)9 | 0.5561 | MW364168 |
| OPTET024b-233bp-vz201 | 10 | UAV | - | (AGTG)10 | 0.0107 | MW364169 |
| OPTET024b-237bp-vz142 | 11 | WZF | - | (AGTG)11 | 0.0749 | MW364170 |
| OPTET024b-241bp-vz135 | 12 | XBF | - | (AGTG)12 | 0.1471 | MW364171 |
Supplementary Table X. Locus OPTET024b - allele nomenclature, repeat structure and frequency.

## Slide 15
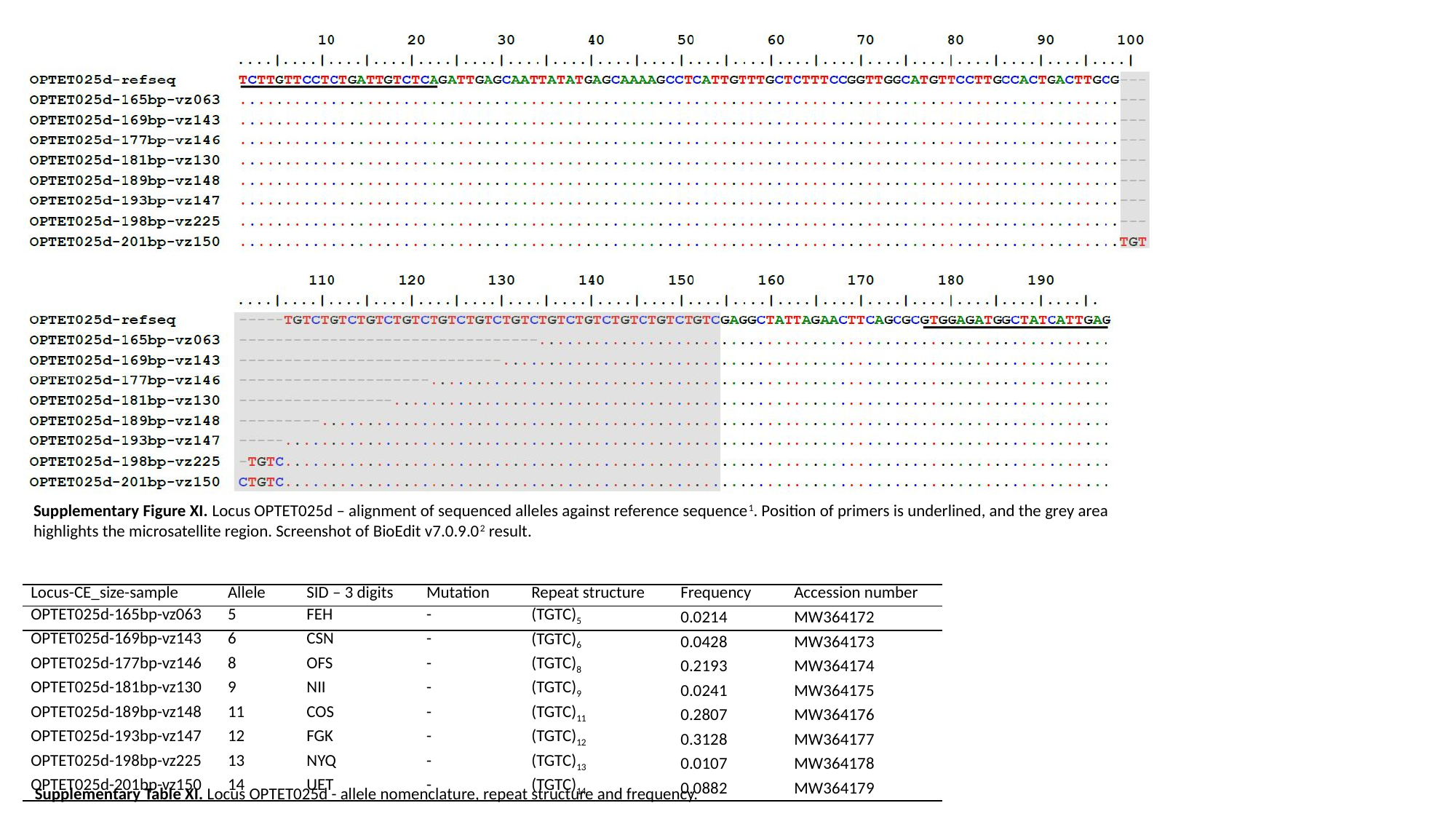

Supplementary Figure XI. Locus OPTET025d – alignment of sequenced alleles against reference sequence1. Position of primers is underlined, and the grey area highlights the microsatellite region. Screenshot of BioEdit v7.0.9.02 result.
| Locus-CE\_size-sample | Allele | SID – 3 digits | Mutation | Repeat structure | Frequency | Accession number |
| --- | --- | --- | --- | --- | --- | --- |
| OPTET025d-165bp-vz063 | 5 | FEH | - | (TGTC)5 | 0.0214 | MW364172 |
| OPTET025d-169bp-vz143 | 6 | CSN | - | (TGTC)6 | 0.0428 | MW364173 |
| OPTET025d-177bp-vz146 | 8 | OFS | - | (TGTC)8 | 0.2193 | MW364174 |
| OPTET025d-181bp-vz130 | 9 | NII | - | (TGTC)9 | 0.0241 | MW364175 |
| OPTET025d-189bp-vz148 | 11 | COS | - | (TGTC)11 | 0.2807 | MW364176 |
| OPTET025d-193bp-vz147 | 12 | FGK | - | (TGTC)12 | 0.3128 | MW364177 |
| OPTET025d-198bp-vz225 | 13 | NYQ | - | (TGTC)13 | 0.0107 | MW364178 |
| OPTET025d-201bp-vz150 | 14 | UET | - | (TGTC)14 | 0.0882 | MW364179 |
Supplementary Table XI. Locus OPTET025d - allele nomenclature, repeat structure and frequency.

## Slide 16
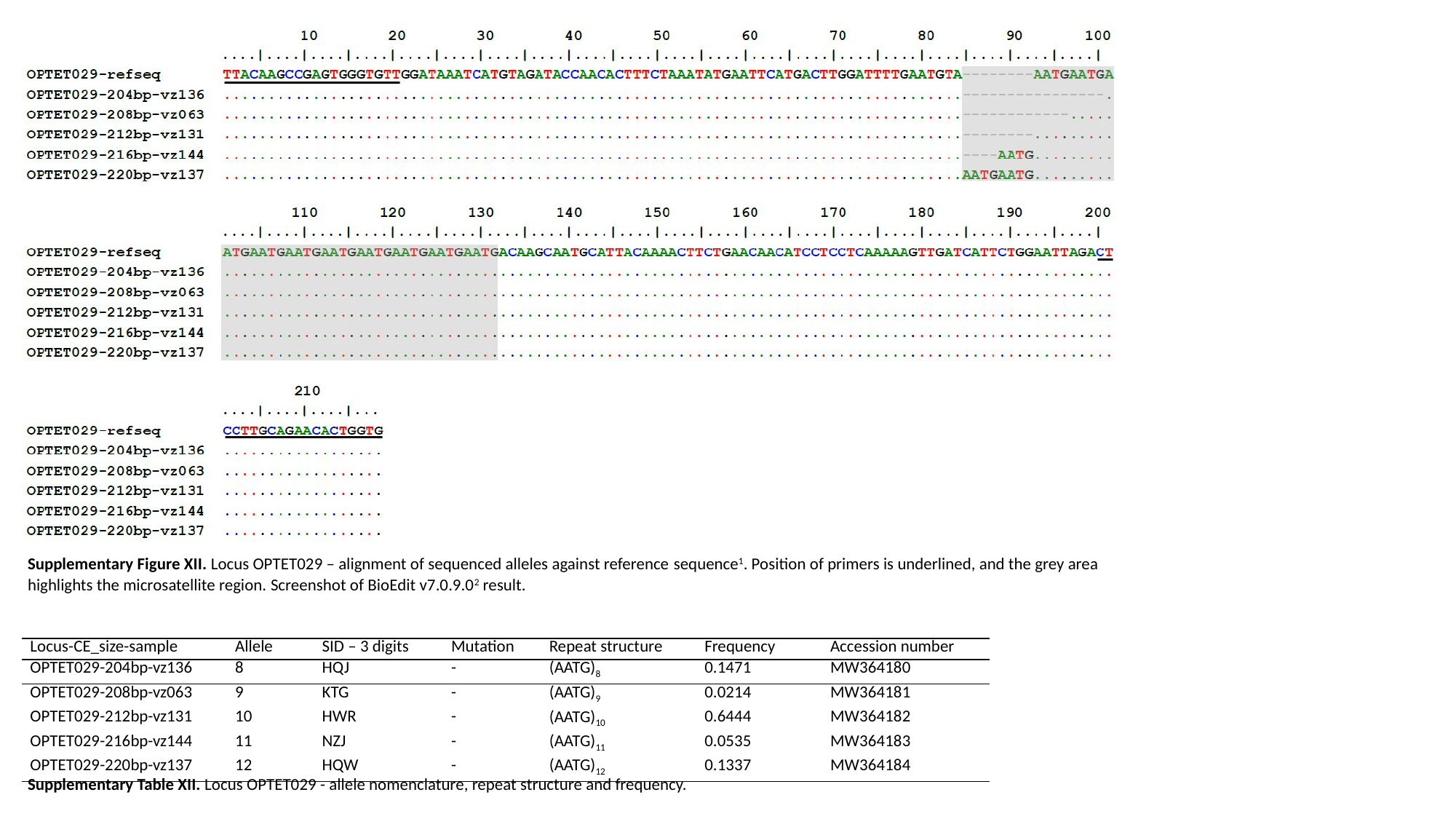

Supplementary Figure XII. Locus OPTET029 – alignment of sequenced alleles against reference sequence1. Position of primers is underlined, and the grey area highlights the microsatellite region. Screenshot of BioEdit v7.0.9.02 result.
| Locus-CE\_size-sample | Allele | SID – 3 digits | Mutation | Repeat structure | Frequency | Accession number |
| --- | --- | --- | --- | --- | --- | --- |
| OPTET029-204bp-vz136 | 8 | HQJ | - | (AATG)8 | 0.1471 | MW364180 |
| OPTET029-208bp-vz063 | 9 | KTG | - | (AATG)9 | 0.0214 | MW364181 |
| OPTET029-212bp-vz131 | 10 | HWR | - | (AATG)10 | 0.6444 | MW364182 |
| OPTET029-216bp-vz144 | 11 | NZJ | - | (AATG)11 | 0.0535 | MW364183 |
| OPTET029-220bp-vz137 | 12 | HQW | - | (AATG)12 | 0.1337 | MW364184 |
Supplementary Table XII. Locus OPTET029 - allele nomenclature, repeat structure and frequency.

## Slide 17
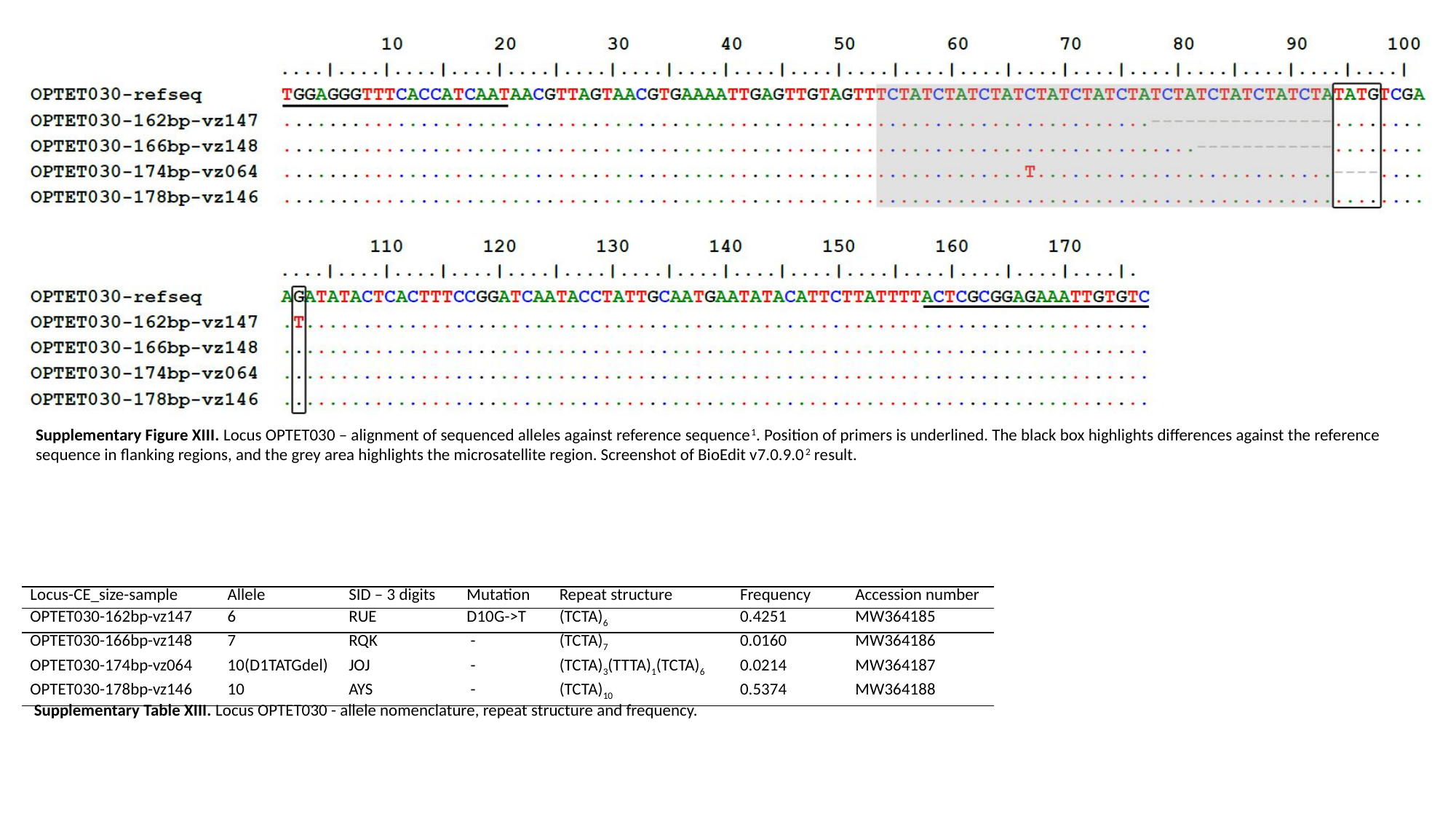

Supplementary Figure XIII. Locus OPTET030 – alignment of sequenced alleles against reference sequence1. Position of primers is underlined. The black box highlights differences against the reference sequence in flanking regions, and the grey area highlights the microsatellite region. Screenshot of BioEdit v7.0.9.02 result.
| Locus-CE\_size-sample | Allele | SID – 3 digits | Mutation | Repeat structure | Frequency | Accession number |
| --- | --- | --- | --- | --- | --- | --- |
| OPTET030-162bp-vz147 | 6 | RUE | D10G->T | (TCTA)6 | 0.4251 | MW364185 |
| OPTET030-166bp-vz148 | 7 | RQK | - | (TCTA)7 | 0.0160 | MW364186 |
| OPTET030-174bp-vz064 | 10(D1TATGdel) | JOJ | - | (TCTA)3(TTTA)1(TCTA)6 | 0.0214 | MW364187 |
| OPTET030-178bp-vz146 | 10 | AYS | - | (TCTA)10 | 0.5374 | MW364188 |
Supplementary Table XIII. Locus OPTET030 - allele nomenclature, repeat structure and frequency.

## Slide 18
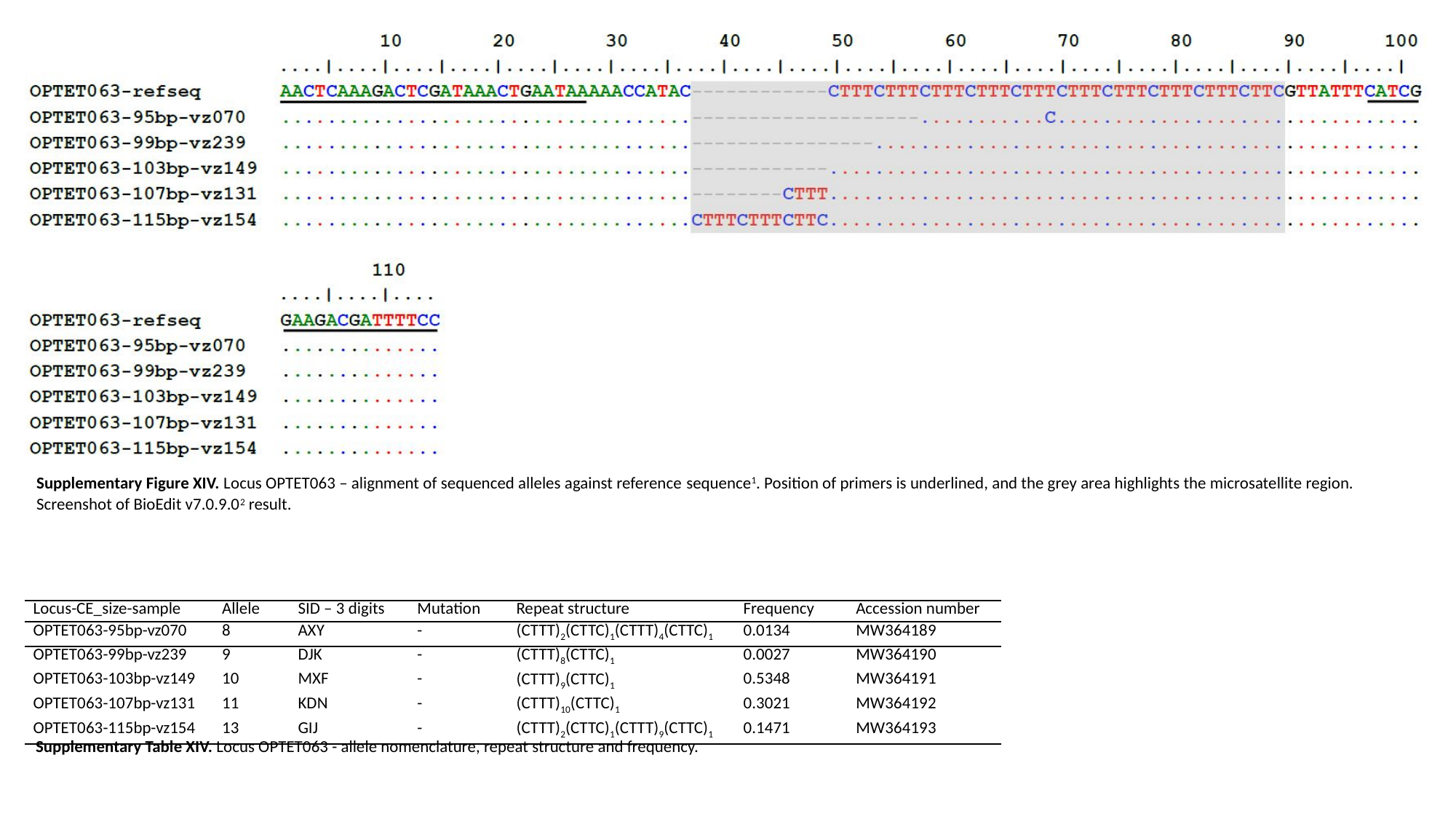

Supplementary Figure XIV. Locus OPTET063 – alignment of sequenced alleles against reference sequence1. Position of primers is underlined, and the grey area highlights the microsatellite region. Screenshot of BioEdit v7.0.9.02 result.
| Locus-CE\_size-sample | Allele | SID – 3 digits | Mutation | Repeat structure | Frequency | Accession number |
| --- | --- | --- | --- | --- | --- | --- |
| OPTET063-95bp-vz070 | 8 | AXY | - | (CTTT)2(CTTC)1(CTTT)4(CTTC)1 | 0.0134 | MW364189 |
| OPTET063-99bp-vz239 | 9 | DJK | - | (CTTT)8(CTTC)1 | 0.0027 | MW364190 |
| OPTET063-103bp-vz149 | 10 | MXF | - | (CTTT)9(CTTC)1 | 0.5348 | MW364191 |
| OPTET063-107bp-vz131 | 11 | KDN | - | (CTTT)10(CTTC)1 | 0.3021 | MW364192 |
| OPTET063-115bp-vz154 | 13 | GIJ | - | (CTTT)2(CTTC)1(CTTT)9(CTTC)1 | 0.1471 | MW364193 |
Supplementary Table XIV. Locus OPTET063 - allele nomenclature, repeat structure and frequency.

## Slide 19
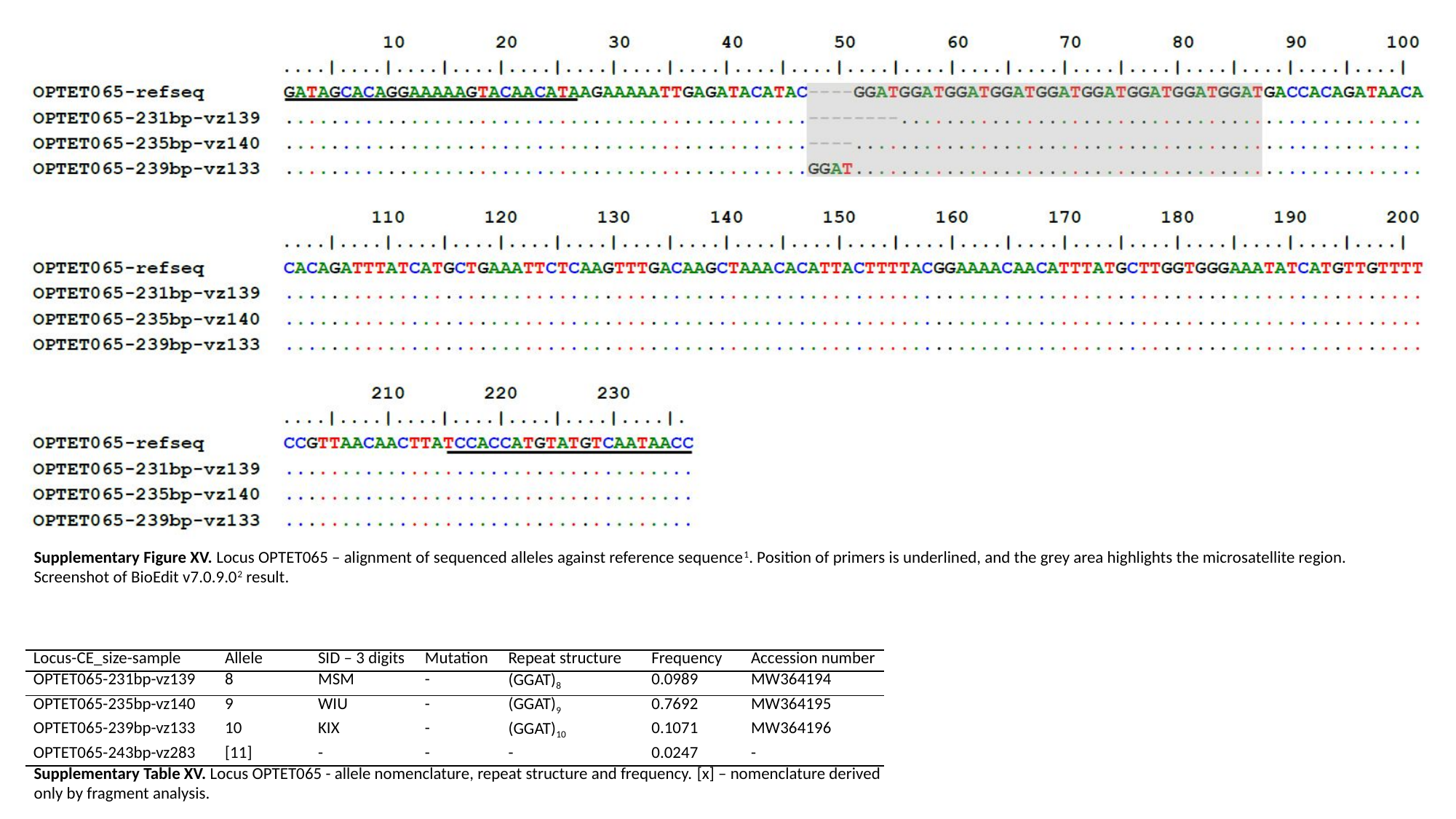

Supplementary Figure XV. Locus OPTET065 – alignment of sequenced alleles against reference sequence1. Position of primers is underlined, and the grey area highlights the microsatellite region. Screenshot of BioEdit v7.0.9.02 result.
| Locus-CE\_size-sample | Allele | SID – 3 digits | Mutation | Repeat structure | Frequency | Accession number |
| --- | --- | --- | --- | --- | --- | --- |
| OPTET065-231bp-vz139 | 8 | MSM | - | (GGAT)8 | 0.0989 | MW364194 |
| OPTET065-235bp-vz140 | 9 | WIU | - | (GGAT)9 | 0.7692 | MW364195 |
| OPTET065-239bp-vz133 | 10 | KIX | - | (GGAT)10 | 0.1071 | MW364196 |
| OPTET065-243bp-vz283 | [11] | - | - | - | 0.0247 | - |
Supplementary Table XV. Locus OPTET065 - allele nomenclature, repeat structure and frequency. [x] – nomenclature derived only by fragment analysis.

## Slide 20
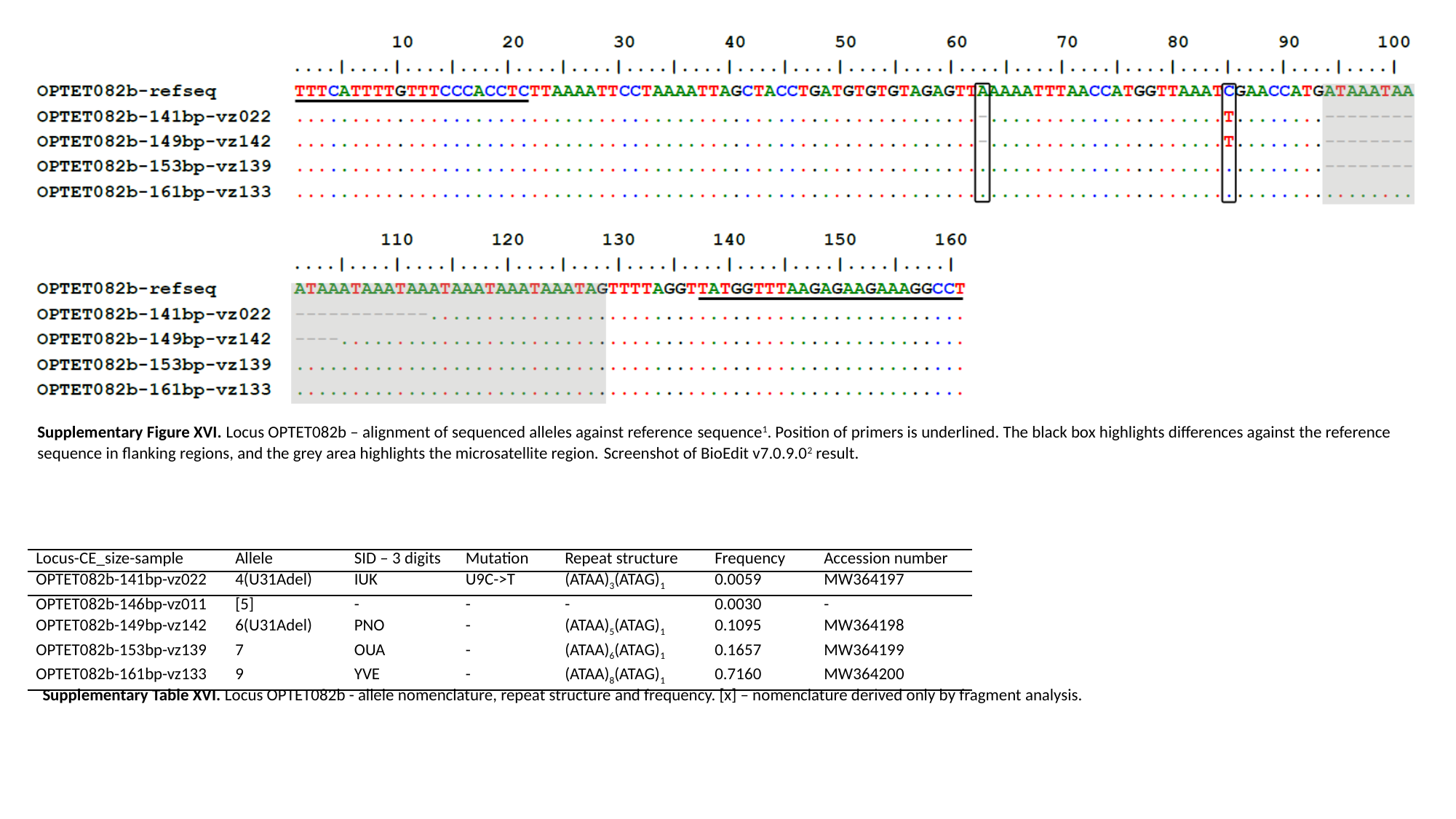

Supplementary Figure XVI. Locus OPTET082b – alignment of sequenced alleles against reference sequence1. Position of primers is underlined. The black box highlights differences against the reference sequence in flanking regions, and the grey area highlights the microsatellite region. Screenshot of BioEdit v7.0.9.02 result.
| Locus-CE\_size-sample | Allele | SID – 3 digits | Mutation | Repeat structure | Frequency | Accession number |
| --- | --- | --- | --- | --- | --- | --- |
| OPTET082b-141bp-vz022 | 4(U31Adel) | IUK | U9C->T | (ATAA)3(ATAG)1 | 0.0059 | MW364197 |
| OPTET082b-146bp-vz011 | [5] | - | - | - | 0.0030 | - |
| OPTET082b-149bp-vz142 | 6(U31Adel) | PNO | - | (ATAA)5(ATAG)1 | 0.1095 | MW364198 |
| OPTET082b-153bp-vz139 | 7 | OUA | - | (ATAA)6(ATAG)1 | 0.1657 | MW364199 |
| OPTET082b-161bp-vz133 | 9 | YVE | - | (ATAA)8(ATAG)1 | 0.7160 | MW364200 |
Supplementary Table XVI. Locus OPTET082b - allele nomenclature, repeat structure and frequency. [x] – nomenclature derived only by fragment analysis.

## Slide 21
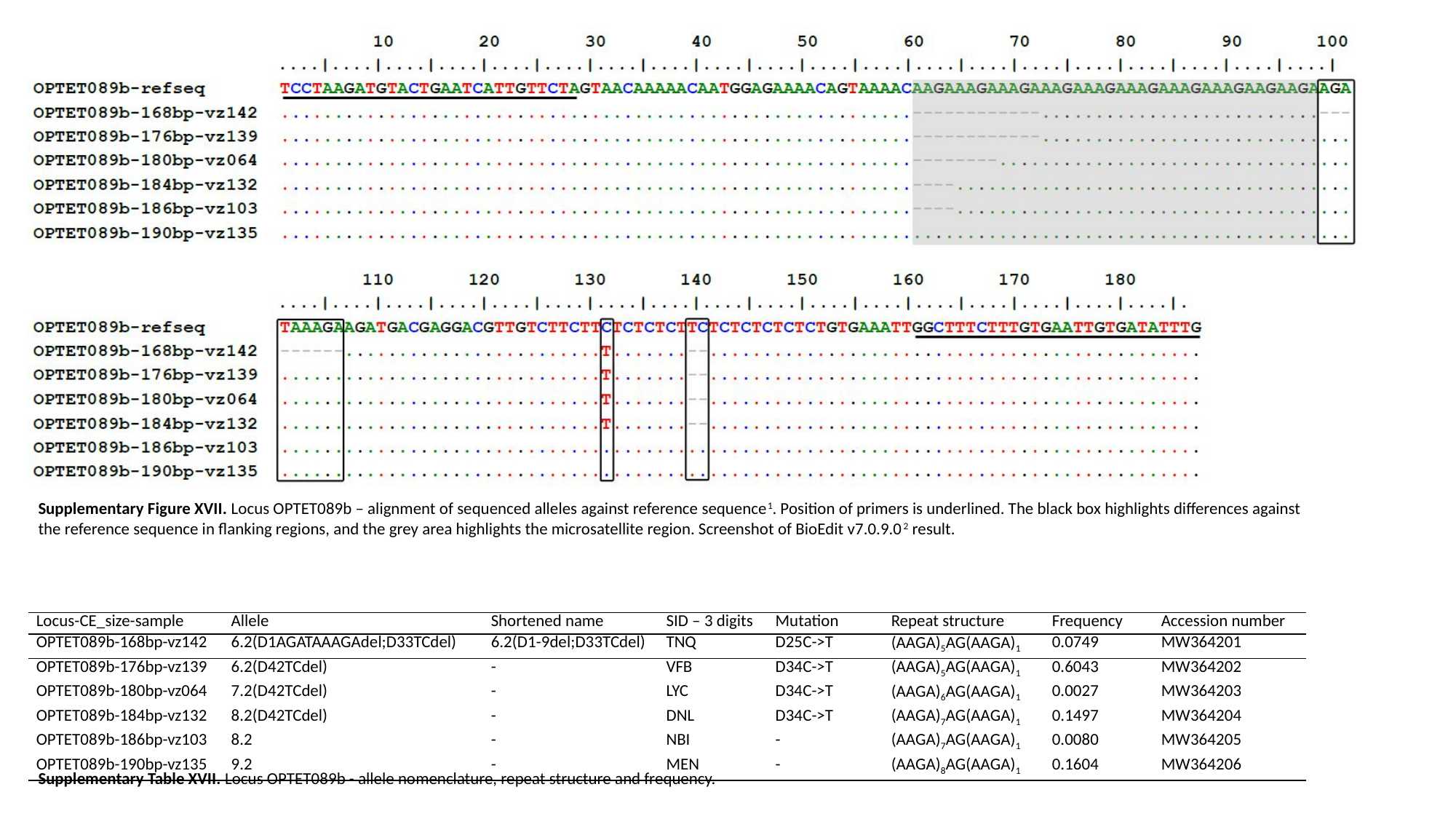

Supplementary Figure XVII. Locus OPTET089b – alignment of sequenced alleles against reference sequence1. Position of primers is underlined. The black box highlights differences against the reference sequence in flanking regions, and the grey area highlights the microsatellite region. Screenshot of BioEdit v7.0.9.02 result.
| Locus-CE\_size-sample | Allele | Shortened name | SID – 3 digits | Mutation | Repeat structure | Frequency | Accession number |
| --- | --- | --- | --- | --- | --- | --- | --- |
| OPTET089b-168bp-vz142 | 6.2(D1AGATAAAGAdel;D33TCdel) | 6.2(D1-9del;D33TCdel) | TNQ | D25C->T | (AAGA)5AG(AAGA)1 | 0.0749 | MW364201 |
| OPTET089b-176bp-vz139 | 6.2(D42TCdel) | - | VFB | D34C->T | (AAGA)5AG(AAGA)1 | 0.6043 | MW364202 |
| OPTET089b-180bp-vz064 | 7.2(D42TCdel) | - | LYC | D34C->T | (AAGA)6AG(AAGA)1 | 0.0027 | MW364203 |
| OPTET089b-184bp-vz132 | 8.2(D42TCdel) | - | DNL | D34C->T | (AAGA)7AG(AAGA)1 | 0.1497 | MW364204 |
| OPTET089b-186bp-vz103 | 8.2 | - | NBI | - | (AAGA)7AG(AAGA)1 | 0.0080 | MW364205 |
| OPTET089b-190bp-vz135 | 9.2 | - | MEN | - | (AAGA)8AG(AAGA)1 | 0.1604 | MW364206 |
Supplementary Table XVII. Locus OPTET089b - allele nomenclature, repeat structure and frequency.

## Slide 22
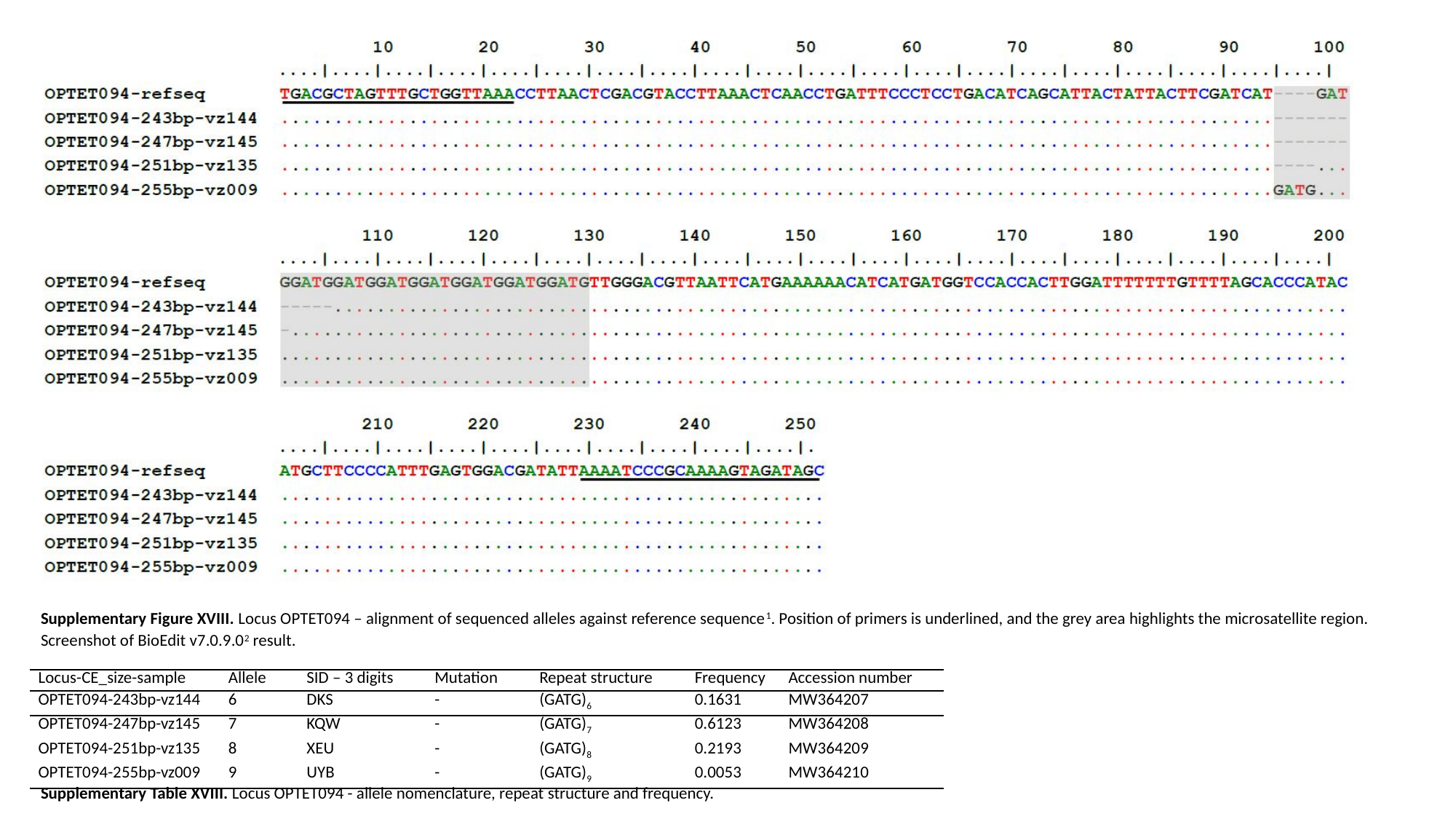

Supplementary Figure XVIII. Locus OPTET094 – alignment of sequenced alleles against reference sequence1. Position of primers is underlined, and the grey area highlights the microsatellite region. Screenshot of BioEdit v7.0.9.02 result.
| Locus-CE\_size-sample | Allele | SID – 3 digits | Mutation | Repeat structure | Frequency | Accession number |
| --- | --- | --- | --- | --- | --- | --- |
| OPTET094-243bp-vz144 | 6 | DKS | - | (GATG)6 | 0.1631 | MW364207 |
| OPTET094-247bp-vz145 | 7 | KQW | - | (GATG)7 | 0.6123 | MW364208 |
| OPTET094-251bp-vz135 | 8 | XEU | - | (GATG)8 | 0.2193 | MW364209 |
| OPTET094-255bp-vz009 | 9 | UYB | - | (GATG)9 | 0.0053 | MW364210 |
Supplementary Table XVIII. Locus OPTET094 - allele nomenclature, repeat structure and frequency.

## Slide 23
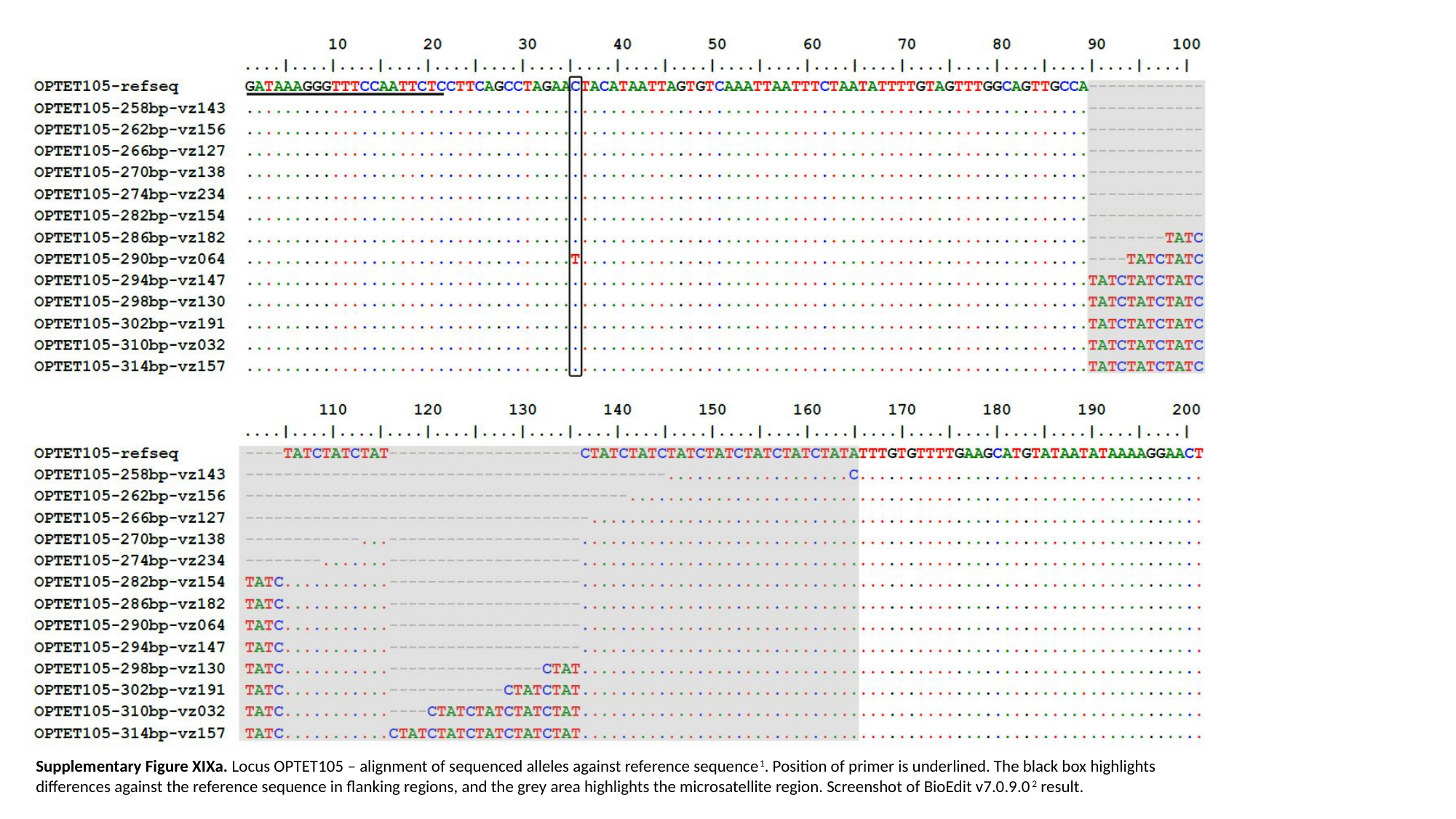

Supplementary Figure XIXa. Locus OPTET105 – alignment of sequenced alleles against reference sequence1. Position of primer is underlined. The black box highlights differences against the reference sequence in flanking regions, and the grey area highlights the microsatellite region. Screenshot of BioEdit v7.0.9.02 result.

## Slide 24
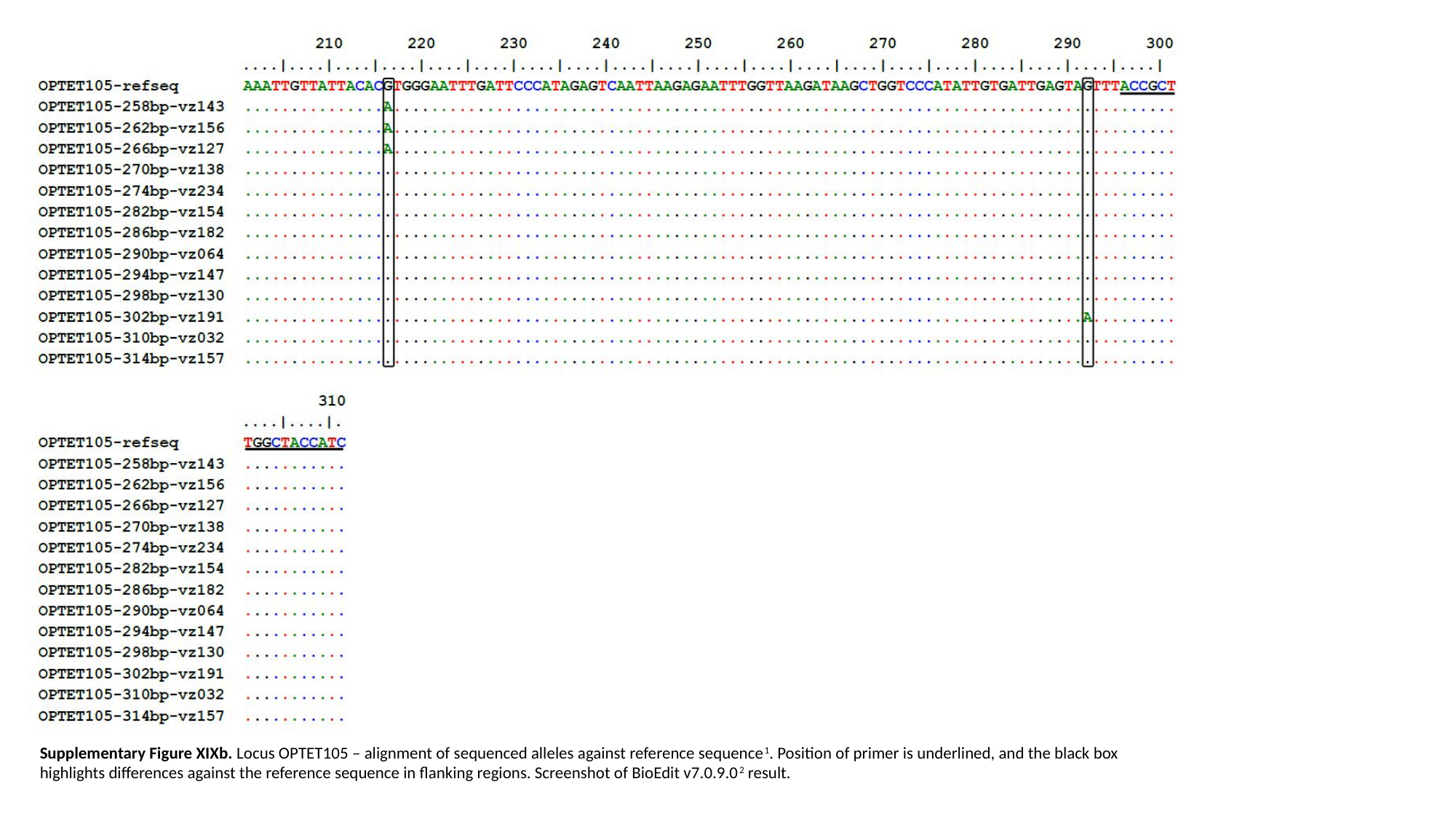

Supplementary Figure XIXb. Locus OPTET105 – alignment of sequenced alleles against reference sequence1. Position of primer is underlined, and the black box highlights differences against the reference sequence in flanking regions. Screenshot of BioEdit v7.0.9.02 result.

## Slide 25
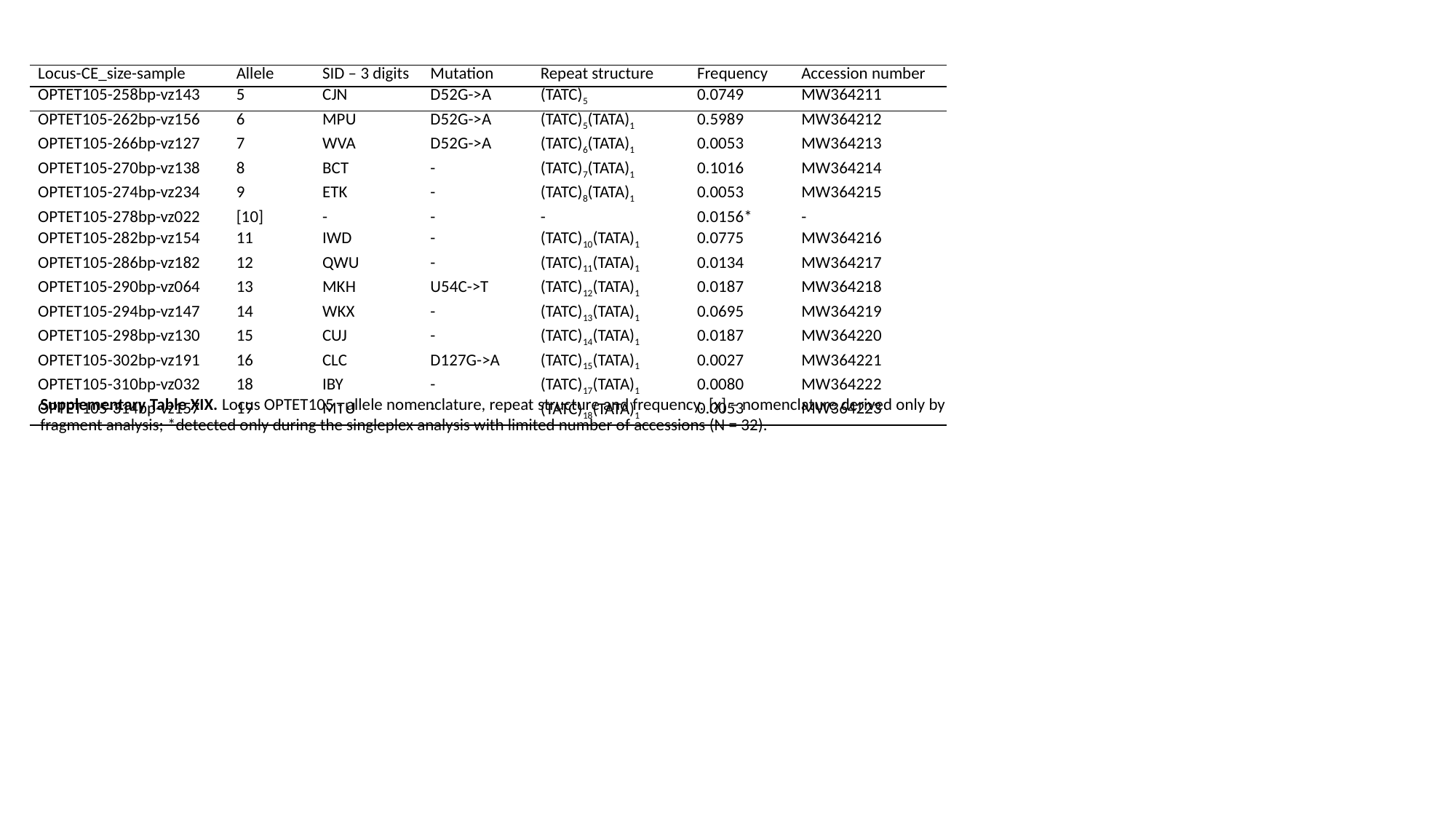

| Locus-CE\_size-sample | Allele | SID – 3 digits | Mutation | Repeat structure | Frequency | Accession number |
| --- | --- | --- | --- | --- | --- | --- |
| OPTET105-258bp-vz143 | 5 | CJN | D52G->A | (TATC)5 | 0.0749 | MW364211 |
| OPTET105-262bp-vz156 | 6 | MPU | D52G->A | (TATC)5(TATA)1 | 0.5989 | MW364212 |
| OPTET105-266bp-vz127 | 7 | WVA | D52G->A | (TATC)6(TATA)1 | 0.0053 | MW364213 |
| OPTET105-270bp-vz138 | 8 | BCT | - | (TATC)7(TATA)1 | 0.1016 | MW364214 |
| OPTET105-274bp-vz234 | 9 | ETK | - | (TATC)8(TATA)1 | 0.0053 | MW364215 |
| OPTET105-278bp-vz022 | [10] | - | - | - | 0.0156\* | - |
| OPTET105-282bp-vz154 | 11 | IWD | - | (TATC)10(TATA)1 | 0.0775 | MW364216 |
| OPTET105-286bp-vz182 | 12 | QWU | - | (TATC)11(TATA)1 | 0.0134 | MW364217 |
| OPTET105-290bp-vz064 | 13 | MKH | U54C->T | (TATC)12(TATA)1 | 0.0187 | MW364218 |
| OPTET105-294bp-vz147 | 14 | WKX | - | (TATC)13(TATA)1 | 0.0695 | MW364219 |
| OPTET105-298bp-vz130 | 15 | CUJ | - | (TATC)14(TATA)1 | 0.0187 | MW364220 |
| OPTET105-302bp-vz191 | 16 | CLC | D127G->A | (TATC)15(TATA)1 | 0.0027 | MW364221 |
| OPTET105-310bp-vz032 | 18 | IBY | - | (TATC)17(TATA)1 | 0.0080 | MW364222 |
| OPTET105-314bp-vz157 | 19 | MTU | - | (TATC)18(TATA)1 | 0.0053 | MW364223 |
Supplementary Table XIX. Locus OPTET105 - allele nomenclature, repeat structure and frequency. [x] – nomenclature derived only by fragment analysis; *detected only during the singleplex analysis with limited number of accessions (N = 32).

## Slide 26
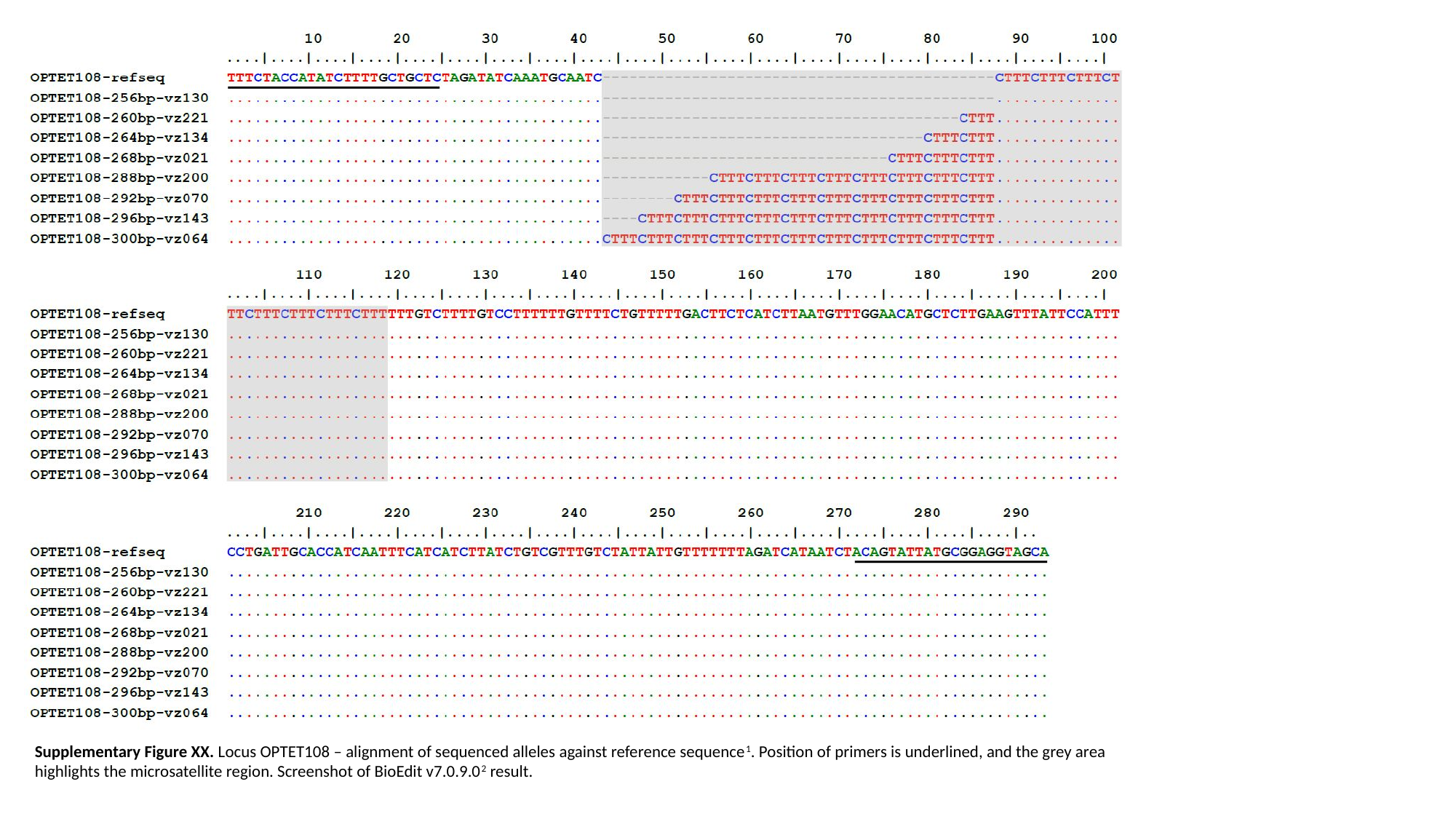

Supplementary Figure XX. Locus OPTET108 – alignment of sequenced alleles against reference sequence1. Position of primers is underlined, and the grey area highlights the microsatellite region. Screenshot of BioEdit v7.0.9.02 result.

## Slide 27
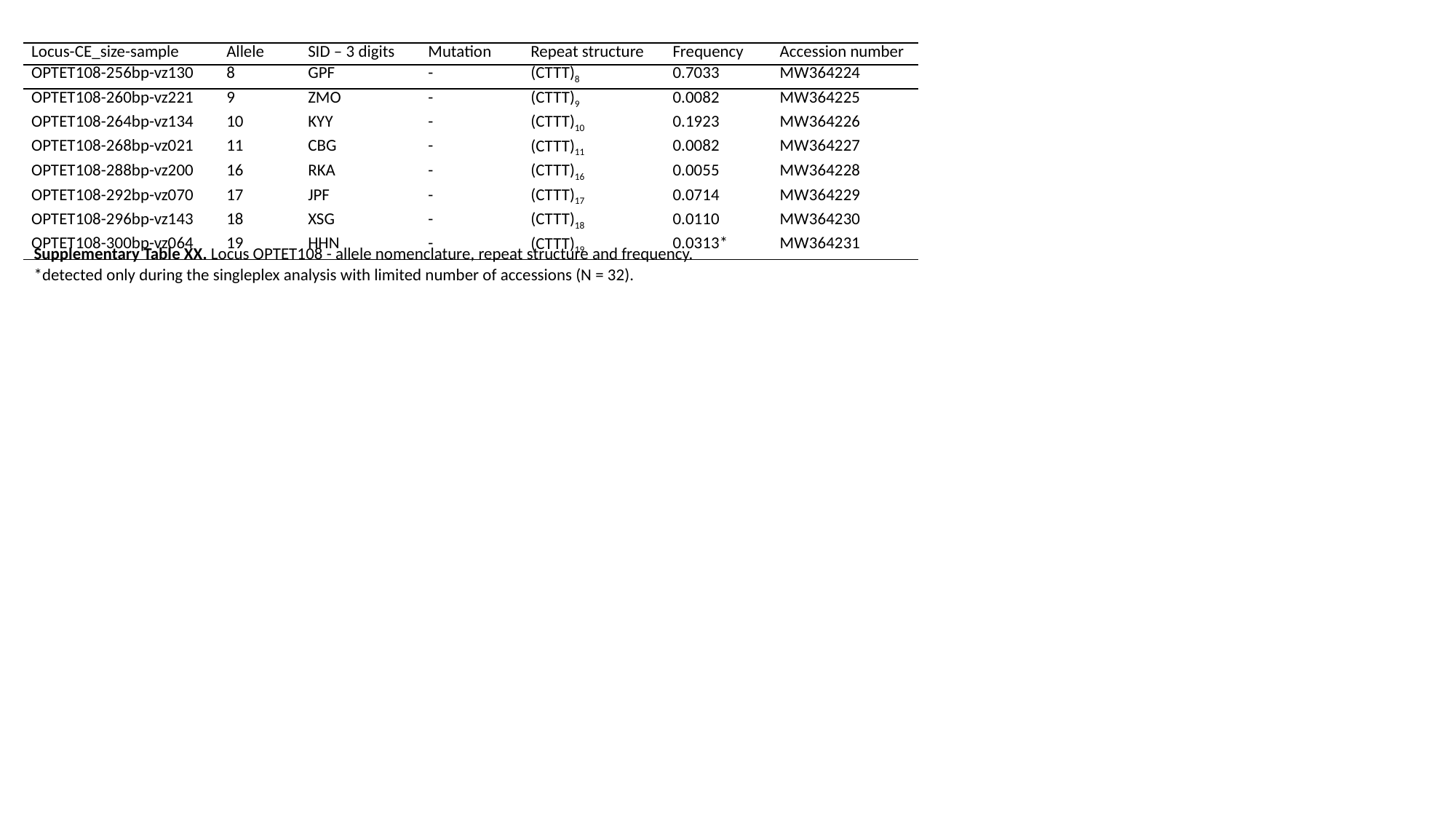

| Locus-CE\_size-sample | Allele | SID – 3 digits | Mutation | Repeat structure | Frequency | Accession number |
| --- | --- | --- | --- | --- | --- | --- |
| OPTET108-256bp-vz130 | 8 | GPF | - | (CTTT)8 | 0.7033 | MW364224 |
| OPTET108-260bp-vz221 | 9 | ZMO | - | (CTTT)9 | 0.0082 | MW364225 |
| OPTET108-264bp-vz134 | 10 | KYY | - | (CTTT)10 | 0.1923 | MW364226 |
| OPTET108-268bp-vz021 | 11 | CBG | - | (CTTT)11 | 0.0082 | MW364227 |
| OPTET108-288bp-vz200 | 16 | RKA | - | (CTTT)16 | 0.0055 | MW364228 |
| OPTET108-292bp-vz070 | 17 | JPF | - | (CTTT)17 | 0.0714 | MW364229 |
| OPTET108-296bp-vz143 | 18 | XSG | - | (CTTT)18 | 0.0110 | MW364230 |
| OPTET108-300bp-vz064 | 19 | HHN | - | (CTTT)19 | 0.0313\* | MW364231 |
Supplementary Table XX. Locus OPTET108 - allele nomenclature, repeat structure and frequency. *detected only during the singleplex analysis with limited number of accessions (N = 32).

## Slide 28
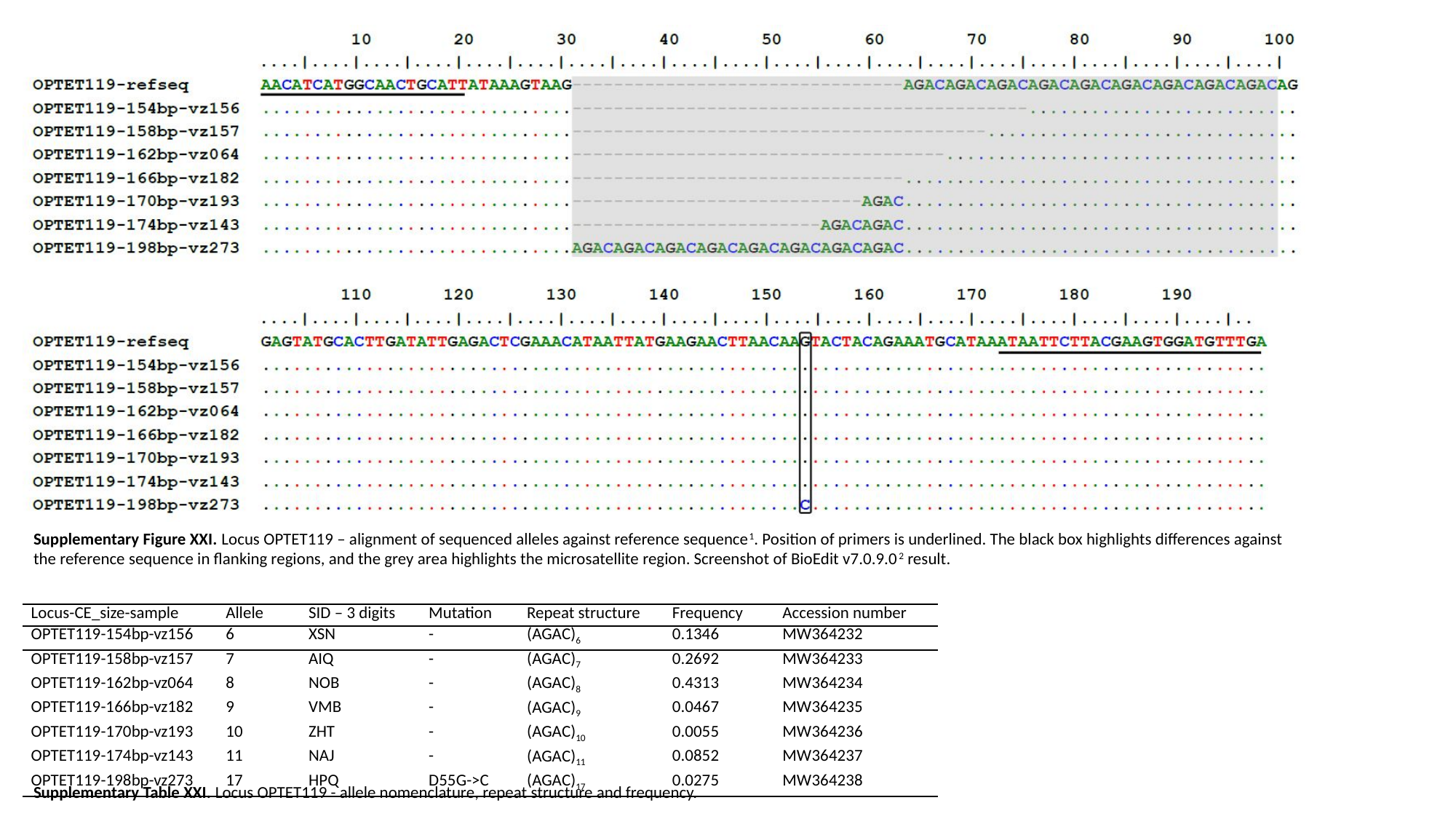

Supplementary Figure XXI. Locus OPTET119 – alignment of sequenced alleles against reference sequence1. Position of primers is underlined. The black box highlights differences against the reference sequence in flanking regions, and the grey area highlights the microsatellite region. Screenshot of BioEdit v7.0.9.02 result.
| Locus-CE\_size-sample | Allele | SID – 3 digits | Mutation | Repeat structure | Frequency | Accession number |
| --- | --- | --- | --- | --- | --- | --- |
| OPTET119-154bp-vz156 | 6 | XSN | - | (AGAC)6 | 0.1346 | MW364232 |
| OPTET119-158bp-vz157 | 7 | AIQ | - | (AGAC)7 | 0.2692 | MW364233 |
| OPTET119-162bp-vz064 | 8 | NOB | - | (AGAC)8 | 0.4313 | MW364234 |
| OPTET119-166bp-vz182 | 9 | VMB | - | (AGAC)9 | 0.0467 | MW364235 |
| OPTET119-170bp-vz193 | 10 | ZHT | - | (AGAC)10 | 0.0055 | MW364236 |
| OPTET119-174bp-vz143 | 11 | NAJ | - | (AGAC)11 | 0.0852 | MW364237 |
| OPTET119-198bp-vz273 | 17 | HPQ | D55G->C | (AGAC)17 | 0.0275 | MW364238 |
Supplementary Table XXI. Locus OPTET119 - allele nomenclature, repeat structure and frequency.

## Slide 29
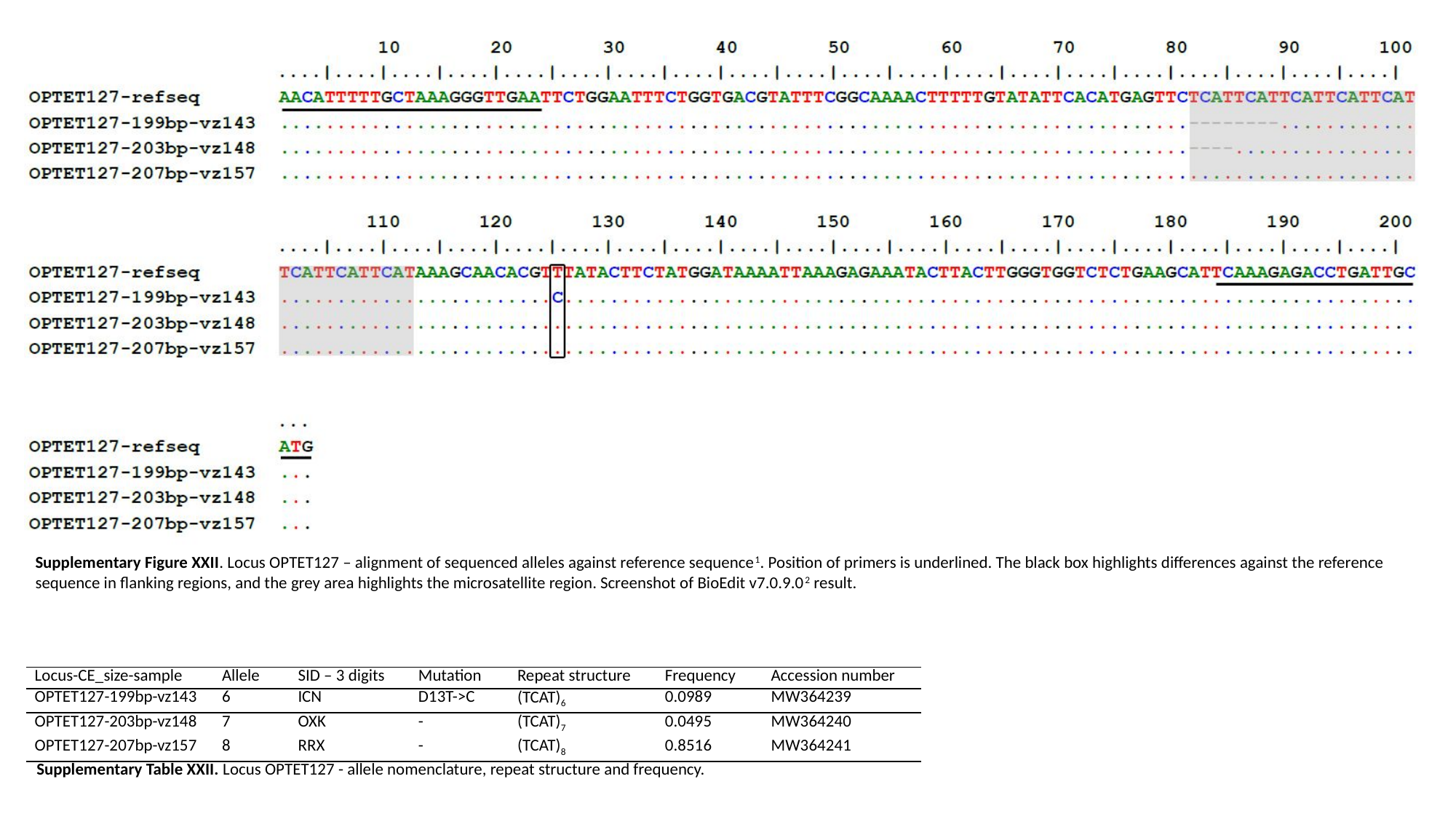

Supplementary Figure XXII. Locus OPTET127 – alignment of sequenced alleles against reference sequence1. Position of primers is underlined. The black box highlights differences against the reference sequence in flanking regions, and the grey area highlights the microsatellite region. Screenshot of BioEdit v7.0.9.02 result.
| Locus-CE\_size-sample | Allele | SID – 3 digits | Mutation | Repeat structure | Frequency | Accession number |
| --- | --- | --- | --- | --- | --- | --- |
| OPTET127-199bp-vz143 | 6 | ICN | D13T->C | (TCAT)6 | 0.0989 | MW364239 |
| OPTET127-203bp-vz148 | 7 | OXK | - | (TCAT)7 | 0.0495 | MW364240 |
| OPTET127-207bp-vz157 | 8 | RRX | - | (TCAT)8 | 0.8516 | MW364241 |
Supplementary Table XXII. Locus OPTET127 - allele nomenclature, repeat structure and frequency.

## Slide 30
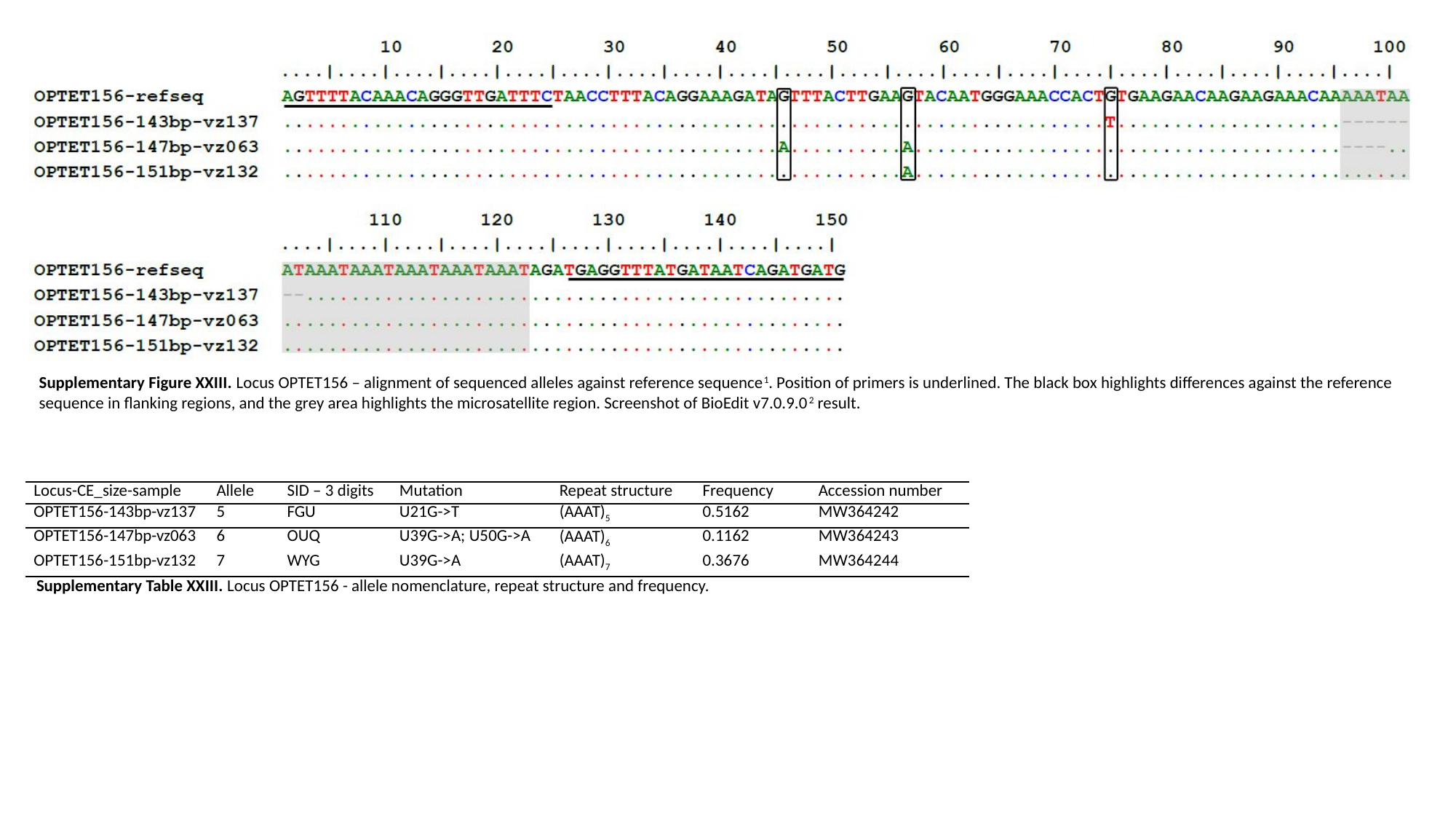

Supplementary Figure XXIII. Locus OPTET156 – alignment of sequenced alleles against reference sequence1. Position of primers is underlined. The black box highlights differences against the reference sequence in flanking regions, and the grey area highlights the microsatellite region. Screenshot of BioEdit v7.0.9.02 result.
| Locus-CE\_size-sample | Allele | SID – 3 digits | Mutation | Repeat structure | Frequency | Accession number |
| --- | --- | --- | --- | --- | --- | --- |
| OPTET156-143bp-vz137 | 5 | FGU | U21G->T | (AAAT)5 | 0.5162 | MW364242 |
| OPTET156-147bp-vz063 | 6 | OUQ | U39G->A; U50G->A | (AAAT)6 | 0.1162 | MW364243 |
| OPTET156-151bp-vz132 | 7 | WYG | U39G->A | (AAAT)7 | 0.3676 | MW364244 |
Supplementary Table XXIII. Locus OPTET156 - allele nomenclature, repeat structure and frequency.

## Slide 31
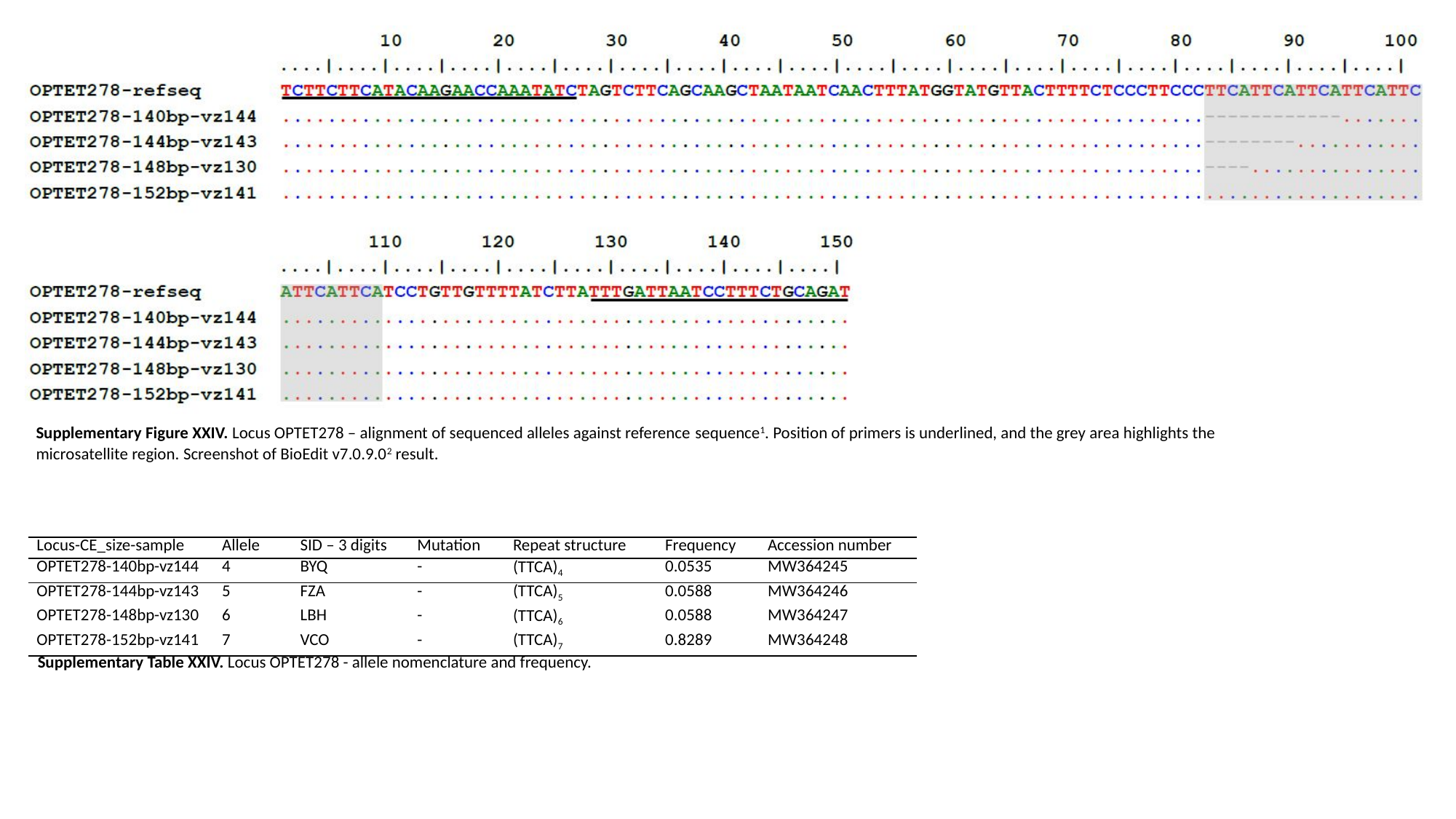

Supplementary Figure XXIV. Locus OPTET278 – alignment of sequenced alleles against reference sequence1. Position of primers is underlined, and the grey area highlights the microsatellite region. Screenshot of BioEdit v7.0.9.02 result.
| Locus-CE\_size-sample | Allele | SID – 3 digits | Mutation | Repeat structure | Frequency | Accession number |
| --- | --- | --- | --- | --- | --- | --- |
| OPTET278-140bp-vz144 | 4 | BYQ | - | (TTCA)4 | 0.0535 | MW364245 |
| OPTET278-144bp-vz143 | 5 | FZA | - | (TTCA)5 | 0.0588 | MW364246 |
| OPTET278-148bp-vz130 | 6 | LBH | - | (TTCA)6 | 0.0588 | MW364247 |
| OPTET278-152bp-vz141 | 7 | VCO | - | (TTCA)7 | 0.8289 | MW364248 |
Supplementary Table XXIV. Locus OPTET278 - allele nomenclature and frequency.

## Slide 32
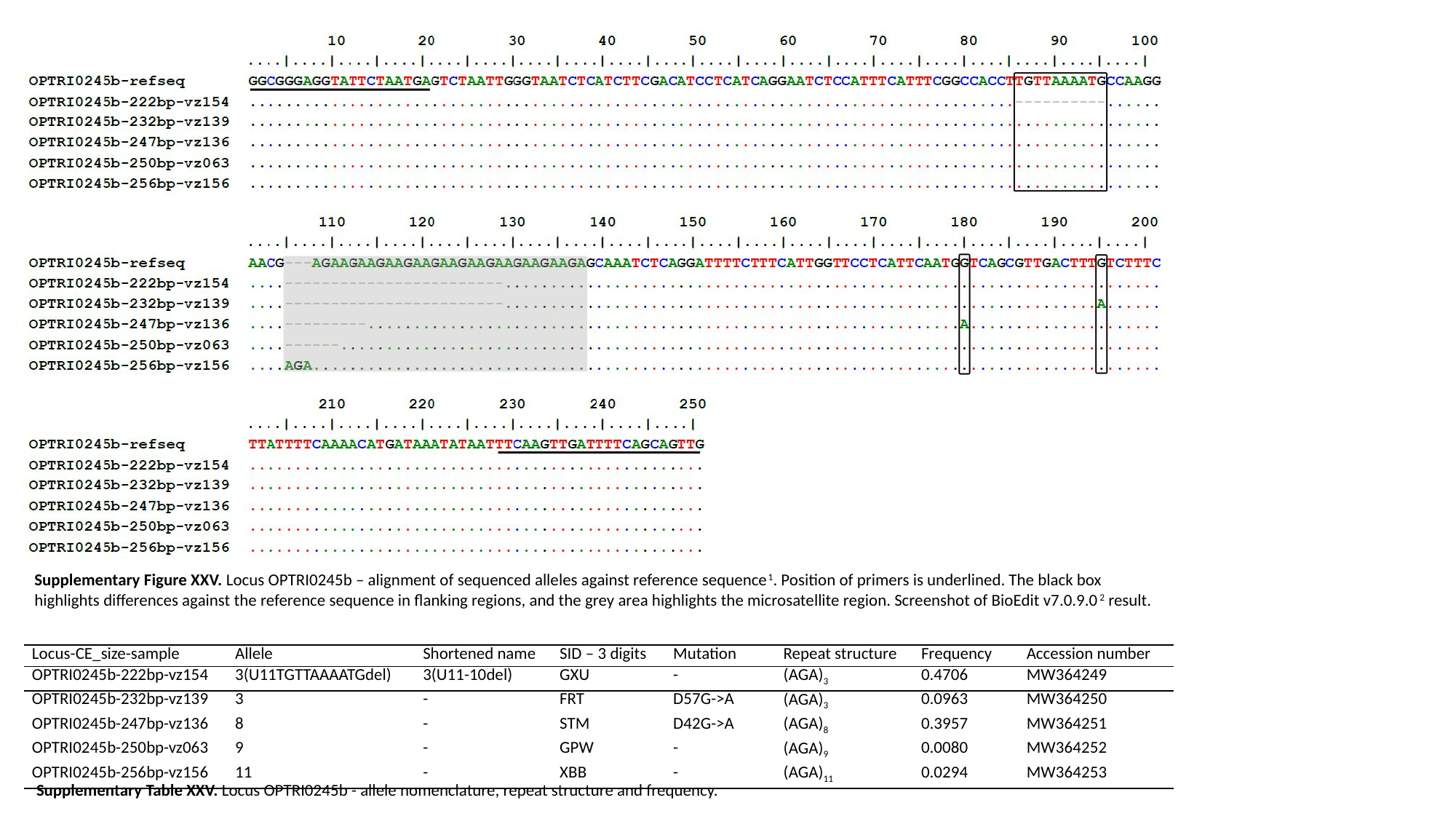

Supplementary Figure XXV. Locus OPTRI0245b – alignment of sequenced alleles against reference sequence1. Position of primers is underlined. The black box highlights differences against the reference sequence in flanking regions, and the grey area highlights the microsatellite region. Screenshot of BioEdit v7.0.9.02 result.
| Locus-CE\_size-sample | Allele | Shortened name | SID – 3 digits | Mutation | Repeat structure | Frequency | Accession number |
| --- | --- | --- | --- | --- | --- | --- | --- |
| OPTRI0245b-222bp-vz154 | 3(U11TGTTAAAATGdel) | 3(U11-10del) | GXU | - | (AGA)3 | 0.4706 | MW364249 |
| OPTRI0245b-232bp-vz139 | 3 | - | FRT | D57G->A | (AGA)3 | 0.0963 | MW364250 |
| OPTRI0245b-247bp-vz136 | 8 | - | STM | D42G->A | (AGA)8 | 0.3957 | MW364251 |
| OPTRI0245b-250bp-vz063 | 9 | - | GPW | - | (AGA)9 | 0.0080 | MW364252 |
| OPTRI0245b-256bp-vz156 | 11 | - | XBB | - | (AGA)11 | 0.0294 | MW364253 |
Supplementary Table XXV. Locus OPTRI0245b - allele nomenclature, repeat structure and frequency.

## Slide 33
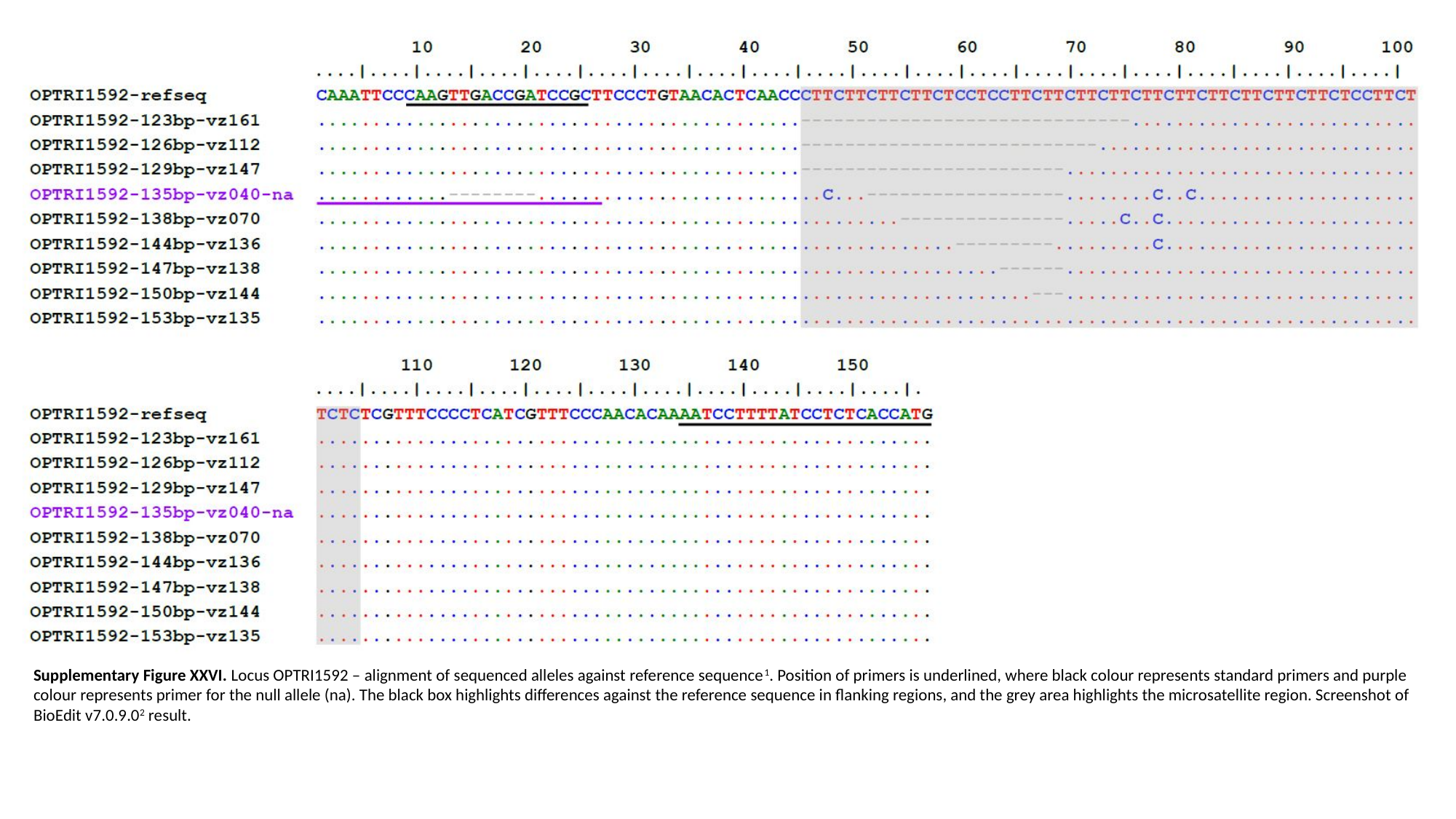

Supplementary Figure XXVI. Locus OPTRI1592 – alignment of sequenced alleles against reference sequence1. Position of primers is underlined, where black colour represents standard primers and purple colour represents primer for the null allele (na). The black box highlights differences against the reference sequence in flanking regions, and the grey area highlights the microsatellite region. Screenshot of BioEdit v7.0.9.02 result.

## Slide 34
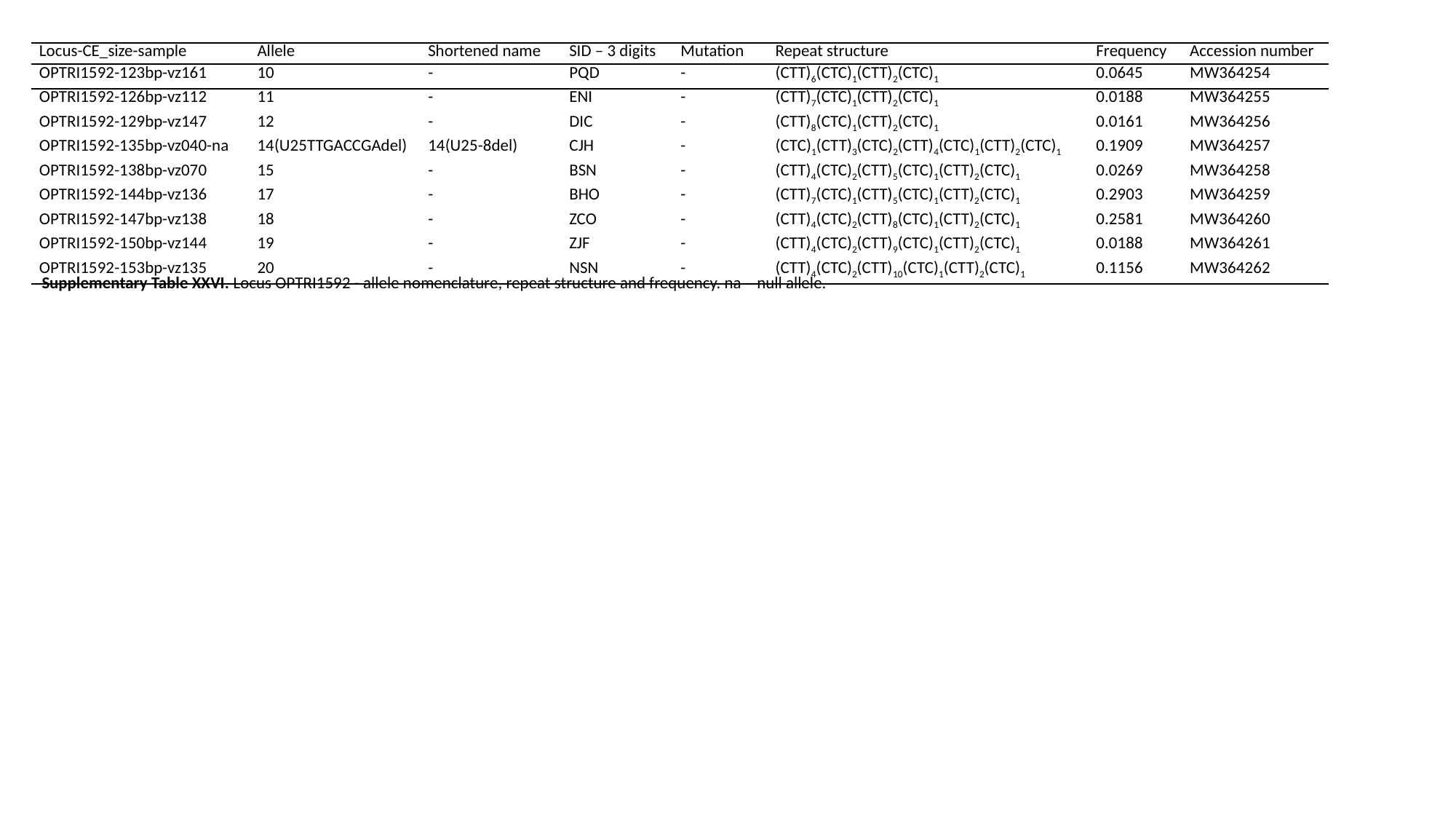

| Locus-CE\_size-sample | Allele | Shortened name | SID – 3 digits | Mutation | Repeat structure | Frequency | Accession number |
| --- | --- | --- | --- | --- | --- | --- | --- |
| OPTRI1592-123bp-vz161 | 10 | - | PQD | - | (CTT)6(CTC)1(CTT)2(CTC)1 | 0.0645 | MW364254 |
| OPTRI1592-126bp-vz112 | 11 | - | ENI | - | (CTT)7(CTC)1(CTT)2(CTC)1 | 0.0188 | MW364255 |
| OPTRI1592-129bp-vz147 | 12 | - | DIC | - | (CTT)8(CTC)1(CTT)2(CTC)1 | 0.0161 | MW364256 |
| OPTRI1592-135bp-vz040-na | 14(U25TTGACCGAdel) | 14(U25-8del) | CJH | - | (CTC)1(CTT)3(CTC)2(CTT)4(CTC)1(CTT)2(CTC)1 | 0.1909 | MW364257 |
| OPTRI1592-138bp-vz070 | 15 | - | BSN | - | (CTT)4(CTC)2(CTT)5(CTC)1(CTT)2(CTC)1 | 0.0269 | MW364258 |
| OPTRI1592-144bp-vz136 | 17 | - | BHO | - | (CTT)7(CTC)1(CTT)5(CTC)1(CTT)2(CTC)1 | 0.2903 | MW364259 |
| OPTRI1592-147bp-vz138 | 18 | - | ZCO | - | (CTT)4(CTC)2(CTT)8(CTC)1(CTT)2(CTC)1 | 0.2581 | MW364260 |
| OPTRI1592-150bp-vz144 | 19 | - | ZJF | - | (CTT)4(CTC)2(CTT)9(CTC)1(CTT)2(CTC)1 | 0.0188 | MW364261 |
| OPTRI1592-153bp-vz135 | 20 | - | NSN | - | (CTT)4(CTC)2(CTT)10(CTC)1(CTT)2(CTC)1 | 0.1156 | MW364262 |
Supplementary Table XXVI. Locus OPTRI1592 - allele nomenclature, repeat structure and frequency. na – null allele.

## Slide 35
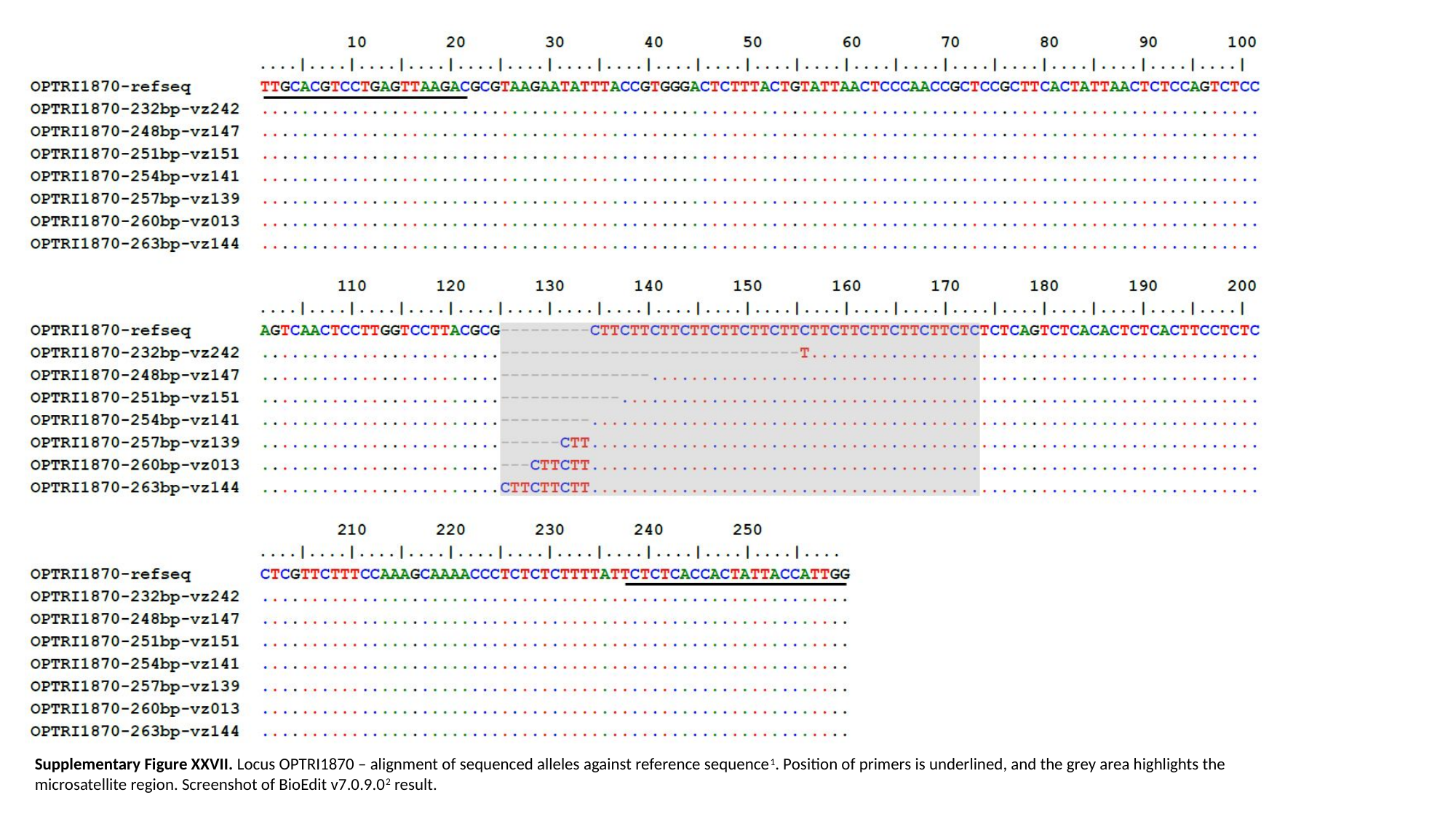

Supplementary Figure XXVII. Locus OPTRI1870 – alignment of sequenced alleles against reference sequence1. Position of primers is underlined, and the grey area highlights the microsatellite region. Screenshot of BioEdit v7.0.9.02 result.

## Slide 36
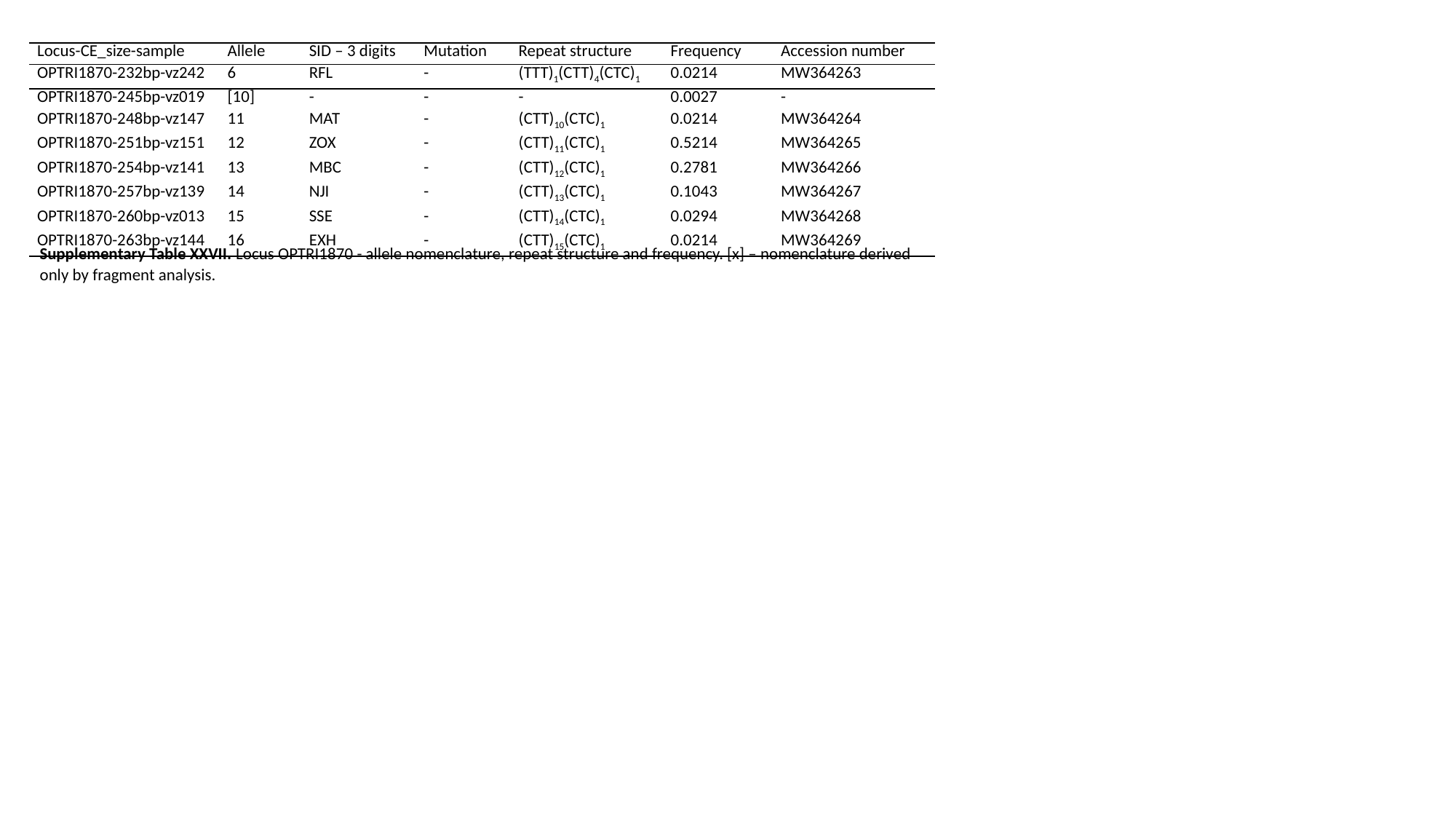

| Locus-CE\_size-sample | Allele | SID – 3 digits | Mutation | Repeat structure | Frequency | Accession number |
| --- | --- | --- | --- | --- | --- | --- |
| OPTRI1870-232bp-vz242 | 6 | RFL | - | (TTT)1(CTT)4(CTC)1 | 0.0214 | MW364263 |
| OPTRI1870-245bp-vz019 | [10] | - | - | - | 0.0027 | - |
| OPTRI1870-248bp-vz147 | 11 | MAT | - | (CTT)10(CTC)1 | 0.0214 | MW364264 |
| OPTRI1870-251bp-vz151 | 12 | ZOX | - | (CTT)11(CTC)1 | 0.5214 | MW364265 |
| OPTRI1870-254bp-vz141 | 13 | MBC | - | (CTT)12(CTC)1 | 0.2781 | MW364266 |
| OPTRI1870-257bp-vz139 | 14 | NJI | - | (CTT)13(CTC)1 | 0.1043 | MW364267 |
| OPTRI1870-260bp-vz013 | 15 | SSE | - | (CTT)14(CTC)1 | 0.0294 | MW364268 |
| OPTRI1870-263bp-vz144 | 16 | EXH | - | (CTT)15(CTC)1 | 0.0214 | MW364269 |
Supplementary Table XXVII. Locus OPTRI1870 - allele nomenclature, repeat structure and frequency. [x] – nomenclature derived only by fragment analysis.

## Slide 37
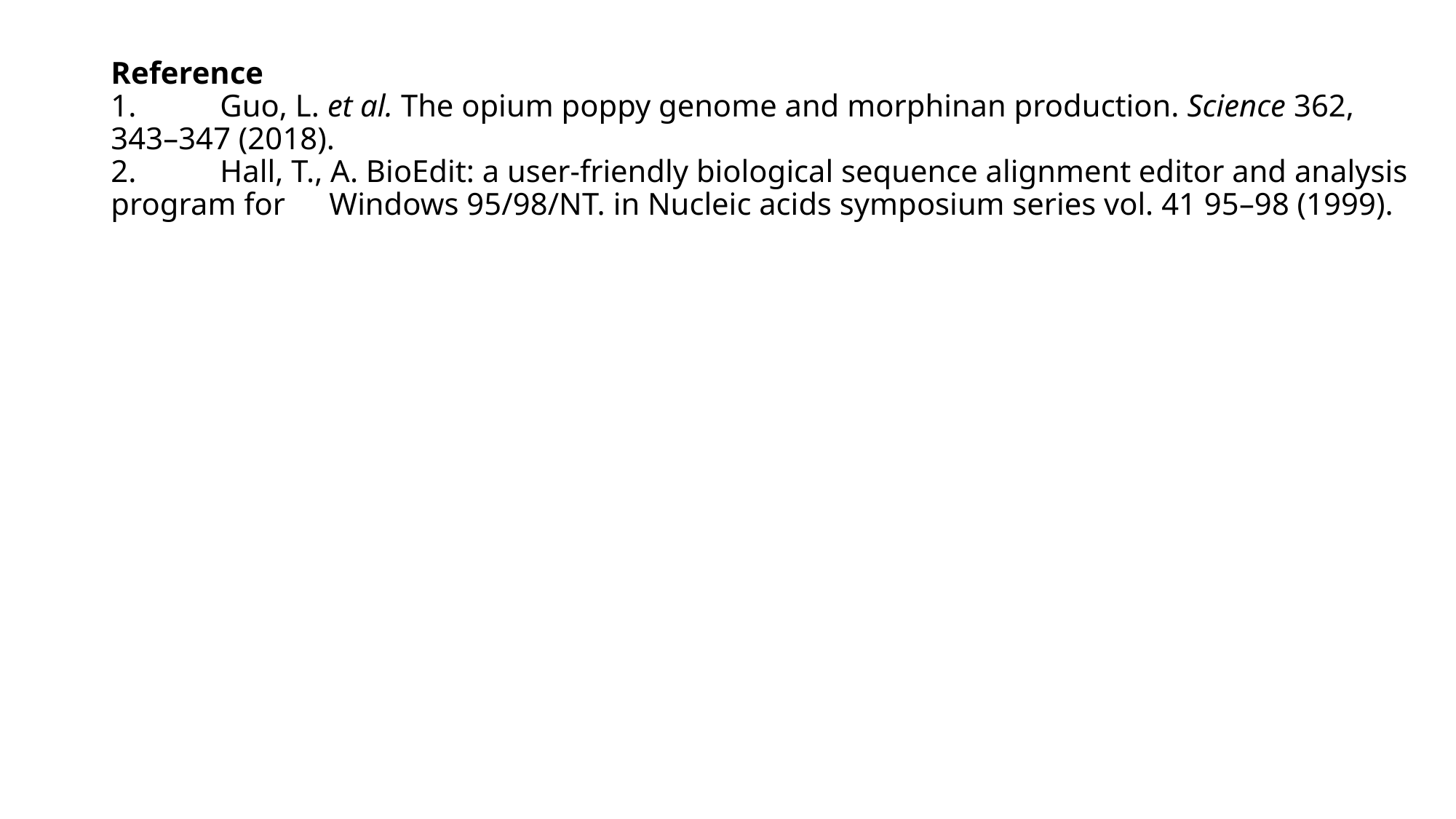

# Reference 1.	Guo, L. et al. The opium poppy genome and morphinan production. Science 362, 343–347 (2018). 2.	Hall, T., A. BioEdit: a user-friendly biological sequence alignment editor and analysis program for 	Windows 95/98/NT. in Nucleic acids symposium series vol. 41 95–98 (1999).
