# Supplementary material for: OpiumPlex is a novel microsatellite system for profiling opium poppy (Papaver somniferum L.)
Source: Sci Rep. 2021 Jun 17;11:12799. doi: 10.1038/s41598-021-91962-1 (PMC8211840; doi:10.1038/s41598-021-91962-1)
Supplement: Supplementary file 3 — Supplementary Information 3. [file 41598_2021_91962_MOESM3_ESM.docx]

OpiumPlex is a novel microsatellite system for profiling opium poppy (*Papaver somniferum* L.)

Jakub Vašek^1^, Daniela Čílová^1^, Martina Melounová^1^, Pavel Svoboda^2^, Kamila Zdeňková^3^, Eliška Čermáková^3^, Jaroslava Ovesná^2^

^1^Department of Genetics and Breeding, FAFNR, Czech University of Life Sciences, Prague, Czech Republic

^2^Division of Crop Genetics and Breeding, Crop Research Institute, Prague, Czech Republic

^3^Department of Biochemistry and Microbiology, University of Chemistry and Technology (UCT Prague), Czech Republic

| MTP | Locus | Dye | c [μM] | Range [bp] | F primer | Sequence (5' – 3') | Rpig primer | Sequence (5' – 3') |
| --- | --- | --- | --- | --- | --- | --- | --- | --- |
| 1 | OPPEN12 | VIC | 0.2 | 191-201 | OPPEN12 | GTTTTACCAAACTGTGCATCAA | OPPEN12 | gtttcttAATTATCCAACACCCGGACA |
| 1 | OPPEN18 | PET | 0.3 | 200-214 | OPPEN18 | AGAACCAAAGGACTAACAGGG | OPPEN18 | gtttcttCGAAAGTTTGGAGCAGTCTTT |
| 1 | OPPEN22 | NED | 0.2 | 203-218 | OPPEN22 | TATGTTAGACCCCCGCGT | OPPEN22 | gtttcttCAATTGCAGTAAGGTATAACGTCAC |
| 1 |  | NED | 0.2 |  | OPPEN22-na | TATGTTAGACCCCCGCATTT |  |  |
| 1 | OPTET029 | 6FAM | 0.2 | 204-220 | OPTET029 | TTACAAGCCGAGTGGGTGTT | OPTET029 | gtttcttCACCAGTGTTCTGCAAGGAG |
| 1 | OPTET030 | 6FAM | 0.2 | 162-178 | OPTET030 | TGGAGGGTTTCACCATCAAT | OPTET030 | gtttcttGACACAATTTCTCCGCGAGT |
| 1 | OPTET063 | NED | 0.2 | 95-115 | OPTET063 | AACTCAAAGACTCGATAAACTGAATAA | OPTET063 | gtttcttGGAAAATCGTCTTCCGATG |
| 1 | OPTET156 | VIC | 0.1 | 143-151 | OPTET156 | AGTTTTACAAACAGGGTTGATTTC | OPTET156 | gtttcttCATCATCTGATTATCATAAACCTCA |
| 1 | OPTET278 | NED | 0.2 | 140-152 | OPTET278 | TCTTCTTCATACAAGAACCAAATATC | OPTET278 | gtttcttATCTGCAGAAAGGATTAATCAAA |
| 1 | OPTRI1592 | PET | 0.3 | 123-153 | OPTRI1592 | CAAGTTGACCGATCCGC | OPTRI1592 | gtttcttCATGGTGAGAGGATAAAAGGATT |
| 1 |  | PET | 0.3 |  | OPTRI1592-na | CAAATTCCCAAGTCCGCT |  |  |
| 2 | OPPEN29 | 6FAM | 0.2 | 153-184 | OPPEN29 | AACAGGACTGGACAGGGC | OPPEN29 | gtttcttGTGAAAGAAATTTTATGCCCGA |
| 2 | OPPEN30 | VIC | 0.1 | 168-197 | OPPEN30 | CCCAATGCGACTTTGTAATTAG | OPPEN30 | gtttcttTCACAGTTGACCAGGTTCATTAT |
| 2 | OPTET024b | 6FAM | 0.2 | 216-241 | OPTET024b | ATCGTGGTGGAATGAGTGTT | OPTET024b | gtttcttGGTTCGTTTGGAGTTACACAA |
| 2 | OPTET025d | NED | 0.2 | 165-201 | OPTET025c | TCTTGTTCCTCTGATTGTCTCA | OPTET025c | gtttcttCTCAATGATAGCCATCTCCAC |
| 2 | OPTET089b | PET | 0.3 | 168-190 | OPTET089 | TCCTAAGATGTACTGAATCATTGTTCTA | OPTET089b | gtttcttCAAATATCACAATTCACAAAGAAAGCC |
| 2 | OPTET094 | NED | 0.2 | 243-255 | OPTET094 | TGACGCTAGTTTGCTGGTTAAA | OPTET094 | gtttcttGCTATCTACTTTTGCGGGATTTT |
| 2 | OPTET105 | 6FAM | 0.2 | 258-314 | OPTET105 | GATAAAGGGTTTCCAATTCTC | OPTET105 | gtttcttGATGGTAGCCAAGCGGT |
| 2 | OPTRI0245b | PET | 0.3 | 222-256 | OPTRI0245 | GGCGGGAGGTATTCTAATGA | OPTRI0245b | gtttcttCAACTGCTGAAAATCAACTTGAA |
| 2 | OPTRI1870 | VIC | 0.1 | 232-263 | OPTRI1870 | TTGCACGTCCTGAGTTAAGAC | OPTRI1870 | gtttcttCCAATGGTAATAGTGGTGAGAGA |
| 3 | OPPEN09 | VIC | 0.2 | 196-223 | OPPEN09 | CTTCCAGATTTTCCTCAGCA | OPPEN09 | gtttcttAATGATATTAGCAGACAACATCAAACT |
| 3 | OPPEN10 | 6FAM | 0.3 | 243-279 | OPPEN10 | CAATGGGGAGGATTTCTTTT | OPPEN10 | gtttcttAACTCTGTCTCCGCCAAGTC |
| 3 | OPPEN38 | PET | 0.3 | 170-194 | OPPEN38 | TGGATTTCCTCCCTTGTTC | OPPEN38 | gtttcttTGACGGCAGTATGTAAAGCAG |
| 3 | OPPEN41 | NED | 0.2 | 156-203 | OPPEN41 | TGGAGTAAGGTGAAATTGATTTGT | OPPEN41 | gtttcttTTCTATCATATCAGTGTGCATTCATT |
| 3 | OPTET065 | NED | 0.2 | 231-239 | OPTET065 | GATAGCACAGGAAAAAGTACAACATA | OPTET065 | gtttcttGGTTATTGACATACATGGTGGA |
| 3 | OPTET082b | VIC | 0.1 | 147-167 | OPTET082 | TTTCATTTTGTTTCCCACCTC | OPTET082b | gtttcttAGGCCTTTCTTCTCTTAAACCATA |
| 3 | OPTET108 | PET | 0.3 | 247-300 | OPTET108 | TTTCTACCATATCTTTTGCTGCTC | OPTET108 | gtttcttTGCTACCTCCGCATAATACTGT |
| 3 | OPTET119 | 6FAM | 0.2 | 138-198 | OPTET119 | AACATCATGGCAACTGCATT | OPTET119 | gtttcttTCAAACATCCACTTCGTAAGAATTAT |
| 3 | OPTET127 | PET | 0.2 | 199-207 | OPTET127 | AACATTTTTGCTAAAGGGTTGAA | OPTET127 | gtttcttCATGCAATCAGGTCTCTTTGA |

**Supplementary Table S4.** Three developed assays with information about the fluorescent dye used, primer concentration, size range and primer sequences.
