# Supplementary material for: OpiumPlex is a novel microsatellite system for profiling opium poppy (Papaver somniferum L.)
Source: Sci Rep. 2021 Jun 17;11:12799. doi: 10.1038/s41598-021-91962-1 (PMC8211840; doi:10.1038/s41598-021-91962-1)
Supplement: Supplementary file 5 — Supplementary Information 5. [file 41598_2021_91962_MOESM5_ESM.docx]

OpiumPlex is a novel microsatellite system for profiling opium poppy (*Papaver somniferum* L.)

Jakub Vašek^1^, Daniela Čílová^1^, Martina Melounová^1^, Pavel Svoboda^2^, Kamila Zdeňková^3^, Eliška Čermáková^3^, Jaroslava Ovesná^2^

^1^Department of Genetics and Breeding, FAFNR, Czech University of Life Sciences, Prague, Czech Republic

^2^Division of Crop Genetics and Breeding, Crop Research Institute, Prague, Czech Republic

^3^Department of Biochemistry and Microbiology, University of Chemistry and Technology (UCT Prague), Czech Republic

| Locus | Primer | Sequence (5´ - 3´) |
| --- | --- | --- |
| OPPEN22 | OPPEN22-na-1-F | TCTGCATTTACGGTTTCCTCG |
|  | OPPEN22-na-1-R | AGCTTTGGTGGTATGCAAATTT |
|  | OPPEN22-na-2-F* | ACAGGTTGTGCGTTAAGATCA |
|  | OPPEN22-na-2-R* | GTGGTTCTGCTAATCTTATCCG |
| OPPEN38 | OPPEN38-na-1-F | GATTGATGTTATGAAAACGAGGC |
|  | OPPEN38-na-1-R | TCTGCCAGACCAAGATAACG |
|  | OPPEN38-na-2-F | TCATTAATGTCAAGATTCCACAGA |
|  | OPPEN38-na-2-R | ACTTCCAAAATTTAACCCGGT |
|  | OPPEN38-na-3-F | AAAAGAAGGCTTACGGCAG |
|  | OPPEN38-na-3-R | TCACTGAAAAATGAAATGAGCA |
| OPTET119 | OPTET119-na-1-F* | TTGCAATCCAAAGTCTAGAATCTA |
|  | OPTET119-na-1-R* | CCAGTTTCCCGTTCTTGAA |
|  | OPTET119-na-2-F | AGAAGAGATATCAGTCCGTGGA |
|  | OPTET119-na-2-R | AACAACCATTATTTATCTCTCAGTTTTC |
|  | OPTET119-na-3-F | AGAAATGGTTGACAAGTGGAAC |
|  | OPTET119-na-3-R | CCAGTTTCCCGTTCTTGAA |
| OPTRI432b | OPTRI0432b-na-1-F* | TTCGACCGAGCAGTGC |
|  | OPTRI0432b-na-1-R* | CGGCTTCTTGTCCAGGA |
|  | OPTRI0432b-na-2-F | ACATGCTTGAGATATCAACGC |
|  | OPTRI0432b-na-2-R | CATTGCAATGCTTTCTCCTG |
| OPTRI1592 | OPTRI1592-na-1-F* | ACGGCATAATCCTTCCTCTCA |
|  | OPTRI1592-na-1-R* | TTAGGTCTCCATCATCGTCTTC |

**Supplementary Table S6.** List of primers designed for null allele detection. *Primers used for sequencing.

| MTP1 | | MTP2 | | MTP3 | |
| --- | --- | --- | --- | --- | --- |
| Marker | RFU* | Marker | RFU* | Marker | RFU* |
| OPPEN12 | 6694 | OPPEN29 | 3187 | OPPEN09 | 7111 |
| OPPEN18 | 6534 | OPPEN30 | 4154 | OPPEN10 | 6814 |
| OPPEN22 | 8765 | OPTET024b | 3102 | OPPEN38 | 8229 |
| OPTET029 | 7805 | OPTET025d | 7028 | OPPEN41 | 11293 |
| OPTET030 | 7004 | OPTET089b | 3230 | OPTET065 | 7720 |
| OPTET063 | 10362 | OPTET094 | 6008 | OPTET082b | 5551 |
| OPTET156 | 4121 | OPTET105 | 2908 | OPTET108 | 7332 |
| OPTET278 | 9018 | OPTRI0245b | 2750 | OPTET119 | 5401 |
| OPTRI1592 | 8332 | OPTRI1870 | 4849 | OPTET127 | 6906 |

**Supplementary Table S7.** Mean rfu value per marker (ABI 3500 machine). *rfu - relative fluorescence unit.

| Assay | IELB^a^ | |  | Hb^b^ | | | | |  | Hb´^c^ | | | |  | N_het_^d^ |
| --- | --- | --- | --- | --- | --- | --- | --- | --- | --- | --- | --- | --- | --- | --- | --- |
|  | $\bar{x}$ | SD |  | $\bar{x}$ | $\tilde{x}$ | SD | Min. | Max. |  | $\bar{x}$ | $\tilde{x}$ | SD | Min. |  |  |
| MTP1 |  |  |  |  |  |  |  |  |  |  |  |  |  |  |  |
| OPPEN12 | 0.91 | 0.19 |  | 0.99 | 0.98 | 0.21 | 0.34 | 1.63 |  | 0.84 | 0.87 | 0.11 | 0.50 |  | 16 |
| OPPEN18 | 0.92 | 0.18 |  | 0.95 | 0.92 | 0.13 | 0.55 | 1.36 |  | 0.88 | 0.91 | 0.06 | 0.69 |  | 17 |
| OPPEN22 | 1.18 | 0.19 |  | 0.98 | 0.97 | 0.08 | 0.71 | 1.25 |  | 0.93 | 0.92 | 0.04 | 0.79 |  | 8 |
| OPTET029 | 1.11 | 0.20 |  | 0.99 | 0.99 | 0.15 | 0.54 | 1.44 |  | 0.89 | 0.90 | 0.09 | 0.62 |  | 6 |
| OPTET030 | 0.95 | 0.16 |  | 0.92 | 0.93 | 0.11 | 0.58 | 1.25 |  | 0.90 | 0.93 | 0.09 | 0.61 |  | 6 |
| OPTET063 | 1.49 | 0.23 |  | 1.01 | 0.97 | 0.15 | 0.56 | 1.47 |  | 0.89 | 0.91 | 0.07 | 0.65 |  | 15 |
| OPTET156 | 0.55 | 0.12 |  | 1.07 | 1.03 | 0.21 | 0.42 | 1.72 |  | 0.84 | 0.85 | 0.08 | 0.59 |  | 12 |
| OPTET278 | 1.23 | 0.21 |  | 1.00 | 1.03 | 0.13 | 0.59 | 1.40 |  | 0.91 | 0.94 | 0.08 | 0.64 |  | 6 |
| OPTRI1592 | 1.18 | 0.21 |  | 0.96 | 0.90 | 0.19 | 0.38 | 1.54 |  | 0.84 | 0.87 | 0.08 | 0.58 |  | 11 |
| mean | 1.06 | 0.19 |  | 0.98 | 0.97 | 0.15 | 0.52 | 1.45 |  | 0.88 | 0.90 | 0.08 | 0.63 |  | 11 |
| MTP2 |  |  |  |  |  |  |  |  |  |  |  |  |  |  |  |
| OPPEN29 | 0.79 | 0.25 |  | 0.94 | 0.96 | 0.12 | 0.56 | 1.31 |  | 0.89 | 0.91 | 0.07 | 0.65 |  | 13 |
| OPPEN30 | 0.93 | 0.31 |  | 0.89 | 0.89 | 0.13 | 0.50 | 1.29 |  | 0.87 | 0.87 | 0.10 | 0.56 |  | 18 |
| OPTET024b | 0.74 | 0.15 |  | 0.85 | 0.86 | 0.08 | 0.61 | 1.10 |  | 0.85 | 0.86 | 0.08 | 0.61 |  | 8 |
| OPTET025d | 2.23 | 0.54 |  | 0.86 | 0.87 | 0.17 | 0.34 | 1.38 |  | 0.83 | 0.87 | 0.14 | 0.39 |  | 22 |
| OPTET089b | 0.91 | 0.22 |  | 0.87 | 0.82 | 0.12 | 0.51 | 1.24 |  | 0.86 | 0.82 | 0.10 | 0.55 |  | 8 |
| OPTET094 | 1.66 | 0.36 |  | 0.88 | 0.86 | 0.14 | 0.44 | 1.31 |  | 0.84 | 0.85 | 0.09 | 0.56 |  | 14 |
| OPTET105 | 0.49 | 0.25 |  | 0.83 | 0.77 | 0.26 | 0.03 | 1.62 |  | 0.74 | 0.77 | 0.15 | 0.27 |  | 6 |
| OPTRI0245b | 0.67 | 0.15 |  | 0.89 | 0.94 | 0.11 | 0.55 | 1.23 |  | 0.88 | 0.94 | 0.10 | 0.56 |  | 17 |
| OPTRI1870 | 1.25 | 0.26 |  | 0.93 | 0.91 | 0.15 | 0.47 | 1.39 |  | 0.87 | 0.87 | 0.09 | 0.60 |  | 15 |
| OPPEN29 | 0.79 | 0.25 |  | 0.94 | 0.96 | 0.12 | 0.56 | 1.31 |  | 0.89 | 0.91 | 0.07 | 0.53 |  | 13 |
| OPPEN30 | 0.93 | 0.31 |  | 0.89 | 0.89 | 0.13 | 0.50 | 1.29 |  | 0.87 | 0.87 | 0.10 | 0.65 |  | 18 |
| mean | 1.08 | 0.28 |  | 0.88 | 0.88 | 0.14 | 0.45 | 1.32 |  | 0.85 | 0.86 | 0.10 | 0.56 |  | 13 |
| MTP3 |  |  |  |  |  |  |  |  |  |  |  |  |  |  |  |
| OPPEN09 | 0.99 | 0.14 |  | 1.01 | 1.04 | 0.12 | 0.65 | 1.38 |  | 0.90 | 0.91 | 0.06 | 0.72 |  | 10 |
| OPPEN10 | 1.02 | 0.14 |  | 0.94 | 0.96 | 0.10 | 0.62 | 1.26 |  | 0.91 | 0.93 | 0.08 | 0.67 |  | 15 |
| OPPEN38 | 1.22 | 0.18 |  | 0.89 | 0.93 | 0.10 | 0.58 | 1.21 |  | 0.89 | 0.93 | 0.1 | 0.59 |  | 9 |
| OPPEN41 | 1.64 | 0.26 |  | 0.98 | 0.98 | 0.13 | 0.59 | 1.38 |  | 0.89 | 0.92 | 0.06 | 0.69 |  | 12 |
| OPTET065 | 1.08 | 0.20 |  | 0.88 | 0.89 | 0.14 | 0.45 | 1.31 |  | 0.88 | 0.89 | 0.14 | 0.45 |  | 5 |
| OPTET082b | 0.77 | 0.17 |  | 0.94 | 0.90 | 0.16 | 0.43 | 1.44 |  | 0.85 | 0.85 | 0.07 | 0.62 |  | 6 |
| OPTET108 | 1.00 | 0.14 |  | 0.99 | 0.91 | 0.15 | 0.54 | 1.44 |  | 0.89 | 0.89 | 0.03 | 0.79 |  | 3 |
| OPTET119 | 0.76 | 0.13 |  | 0.88 | 0.90 | 0.18 | 0.33 | 1.44 |  | 0.85 | 0.88 | 0.15 | 0.39 |  | 12 |
| OPTET127 | 0.96 | 0.12 |  | 1.07 | 1.06 | 0.11 | 0.74 | 1.40 |  | 0.84 | 0.87 | 0.11 | 0.50 |  | 3 |
| mean | 1.05 | 0.17 |  | 0.95 | 0.95 | 0.13 | 0.55 | 1.36 |  | 0.88 | 0.91 | 0.06 | 0.69 |  | 8 |

**Supplementary Table S8.** Interloci and heterozygote balance characterized by mean ($\bar{x}$), median ($\tilde{x}$) and standard deviation (SD) per locus, lower 99th percentile (Min.) calculated as mean – 3SD and upper 99th percentile (Max.) calculated as mean + 3SD. ^a^interloci balance, ^b^heterozygote balance (Hb method), ^c^heterozygote balance (Hb´ method), ^d^number of heterozygotes per locus.

| Marker | $\bar{x}$ | SD |
| --- | --- | --- |
| OPPEN29 | 0.95 | 0.21 |
| OPPEN30 | 1.74 | 0.33 |
| OPTET024b | 0.72 | 0.10 |
| OPTET025d | 1.48 | 0.25 |
| OPTET089b | 0.49 | 0.09 |
| OPTET094 | 1.40 | 0.30 |
| OPTET105 | 1.02 | 0.18 |
| OPTRI0245b | 0.63 | 0.11 |
| OPTRI1870 | 1.37 | 0.23 |
| mean | 1.09 | 0.2 |

**Supplementary Table S9.** IELB results of modified MTP2 formula for ABI 3500. IELB is characterized by mean ($\bar{x}$) and standard deviation (SD) per locus.

| Locus | Mean | | | | | Median | | | | | Standard deviation | | | | | Number of heterozygotes | | | | |
| --- | --- | --- | --- | --- | --- | --- | --- | --- | --- | --- | --- | --- | --- | --- | --- | --- | --- | --- | --- | --- |
|  | b | c | d | e | f | b | c | d | e | f | b | c | d | e | f | b | c | d | e | f |
| OPPEN09 | 1.04 | 0.92 | 0.96 | 1.28 | 0.58 | 1.03 | 0.91 | 0.90 | 1.23 | 0.61 | 0.14 | 0.16 | 0.31 | 0.75 | 0.33 | 8 | 8 | 9 | 7 | 4 |
| OPPEN10 | 0.99 | 1.02 | 1.11 | 1.05 | 0.88 | 1.02 | 0.99 | 1.12 | 1.10 | 0.62 | 0.09 | 0.2 | 0.27 | 0.10 | 0.67 | 8 | 8 | 7 | 3 | 4 |
| OPPEN12 | 1.01 | 0.93 | 1.00 | 1.47 | 1.14 | 0.97 | 0.95 | 1.12 | 1.46 | 1.11 | 0.09 | 0.18 | 0.25 | 0.61 | 0.39 | 9 | 9 | 9 | 7 | 4 |
| OPPEN18 | 1.30 | 1.26 | 2.06 | 3.49 | 6.49 | 1.26 | 1.27 | 2.19 | 3.32 | 6.63 | 0.26 | 0.25 | 0.99 | 1.67 | 3.70 | 8 | 8 | 9 | 5 | 6 |
| OPPEN22 | 0.96 | 0.94 | 1.01 | 0.98 | 1.30 | 0.87 | 1.02 | 0.94 | 0.98 | 1.15 | 0.21 | 0.19 | 0.21 | - | 0.90 | 5 | 5 | 5 | 1 | 3 |
| OPPEN29 | 0.93 | 0.92 | 1.10 | 1.59 | 0.61 | 0.93 | 0.90 | 1.11 | 1.53 | 0.56 | 0.07 | 0.12 | 0.15 | 0.41 | 0.37 | 7 | 8 | 8 | 6 | 4 |
| OPPEN30 | 0.94 | 1.04 | 0.94 | 1.34 | 2.34 | 0.94 | 0.93 | 0.82 | 1.25 | 2.45 | 0.08 | 0.23 | 0.29 | 0.50 | 0.95 | 9 | 9 | 9 | 7 | 4 |
| OPPEN38 | 0.92 | 1.06 | 1.16 | 0.79 | 1.40 | 0.93 | 1.01 | 1.04 | 0.62 | 1.28 | 0.14 | 0.24 | 0.55 | 0.57 | 0.91 | 6 | 8 | 8 | 4 | 4 |
| OPPEN41 | 0.95 | 0.95 | 0.93 | 1.11 | 1.07 | 0.91 | 0.91 | 0.91 | 1.29 | 1.04 | 0.11 | 0.14 | 0.27 | 0.45 | 0.38 | 9 | 10 | 10 | 6 | 7 |
| OPTET024b | 1.03 | 0.90 | 1.05 | 0.55 | 1.24 | 1.03 | 0.86 | 0.88 | 0.36 | 1.24 | 0.07 | 0.12 | 0.48 | 0.36 | - | 4 | 4 | 4 | 3 | 1 |
| OPTET025d | 0.91 | 0.99 | 1.02 | 1.47 | 1.39 | 0.92 | 0.99 | 0.93 | 1.40 | 0.82 | 0.07 | 0.10 | 0.33 | 0.75 | 1.24 | 8 | 8 | 8 | 7 | 5 |
| OPTET029 | 0.97 | 1.03 | 1.08 | 1.06 | 1.32 | 0.94 | 1.01 | 0.94 | 0.81 | 1.03 | 0.08 | 0.16 | 0.38 | 0.54 | 1.12 | 7 | 7 | 7 | 4 | 4 |
| OPTET030 | 0.98 | 0.88 | 0.98 | 1.57 | 1.00 | 0.94 | 0.90 | 0.96 | 1.03 | 1.03 | 0.17 | 0.09 | 0.23 | 1.01 | 0.13 | 6 | 6 | 6 | 5 | 4 |
| OPTET063 | 1.00 | 0.99 | 0.94 | 0.84 | 0.45 | 1.00 | 0.97 | 0.96 | 0.94 | 0.49 | 0.11 | 0.24 | 0.18 | 0.46 | 0.10 | 11 | 11 | 11 | 9 | 4 |
| OPTET065 | 0.95 | 0.99 | 1.03 | 0.78 | 0.63 | 0.95 | 1.00 | 1.07 | 0.73 | 0.45 | 0.10 | 0.14 | 0.22 | 0.32 | 0.51 | 5 | 5 | 5 | 3 | 3 |
| OPTET082b | 1.00 | 1.06 | 0.92 | - | 1.22 | 1.00 | 1.06 | 0.84 | - | 1.22 | 0.02 | 0.05 | 0.28 | - | - | 2 | 3 | 3 | - | 1 |
| OPTET089b | 0.90 | 0.93 | 0.81 | 1.48 | 0.95 | 0.88 | 0.95 | 0.74 | 1.48 | 0.95 | 0.10 | 0.07 | 0.17 | 0.30 | 0.22 | 5 | 5 | 5 | 3 | 2 |
| OPTET094 | 0.98 | 0.98 | 1.04 | 1.04 | 1.12 | 0.96 | 0.91 | 1.10 | 0.96 | 1.17 | 0.07 | 0.15 | 0.34 | 0.41 | 0.22 | 7 | 7 | 7 | 7 | 4 |
| OPTET105 | 0.93 | 0.94 | 1.02 | 1.14 | 1.73 | 0.96 | 0.91 | 1.04 | 1.12 | 1.28 | 0.16 | 0.09 | 0.18 | 0.08 | 1.10 | 5 | 6 | 6 | 3 | 3 |
| OPTET108 | 0.90 | 0.95 | 2.03 | - | 1.00 | 0.90 | 0.95 | 2.03 | - | 1.00 | - | 0.23 | 0.27 | - | 0.59 | 1 | 2 | 2 | - | 2 |
| OPTET119 | 0.94 | 0.90 | 1.15 | 0.83 | 0.65 | 0.97 | 0.82 | 1.02 | 0.83 | 0.61 | 0.07 | 0.21 | 0.20 | 0.30 | 0.41 | 5 | 5 | 5 | 2 | 3 |
| OPTET127 | 0.92 | 0.90 | 0.97 | - | - | 0.92 | 0.90 | 0.97 | - | - | - | - | - | - | - | 1 | 1 | 1 | - | - |
| OPTET156 | 1.09 | 1.09 | 1.06 | 1.46 | 1.07 | 1.08 | 1.12 | 1.00 | 1.46 | 1.07 | 0.08 | 0.14 | 0.27 | 0.38 | - | 5 | 5 | 5 | 2 | 1 |
| OPTET278 | 0.95 | 1.00 | 1.00 | 0.84 | 0.82 | 0.94 | 1.01 | 1.02 | 0.65 | 1.02 | 0.15 | 0.11 | 0.12 | 0.42 | 0.35 | 4 | 4 | 4 | 3 | 3 |
| OPTRI0245b | 0.91 | 0.98 | 1.04 | 1.41 | 0.83 | 0.92 | 0.93 | 0.93 | 1.57 | 0.72 | 0.09 | 0.13 | 0.50 | 0.43 | 0.44 | 11 | 11 | 10 | 6 | 6 |
| OPTRI1592 | 0.99 | 1.14 | 1.19 | 1.51 | 1.12 | 0.99 | 1.15 | 1.04 | 1.35 | 0.67 | 0.09 | 0.26 | 0.33 | 0.73 | 1.07 | 7 | 7 | 7 | 6 | 3 |
| OPTRI1870 | 0.87 | 0.88 | 0.95 | 0.88 | 1.51 | 0.87 | 0.91 | 0.87 | 0.76 | 1.65 | 0.04 | 0.17 | 0.27 | 0.40 | 0.48 | 8 | 8 | 8 | 6 | 4 |
| mean | 0.97 | 0.98 | 1.09 | 1.25 | 1.30 | 0.96 | 0.97 | 1.06 | 1.18 | 1.23 | 0.11 | 0.16 | 0.31 | 0.52 | 0.72 | 6.33 | 6.59 | 6.59 | 4.79 | 3.58 |

**Supplementary Table S10.** Heterozygote balance (Hb) for samples with 1.25 (b), 0.625 (c), 0.313 (d), 0.156 (e) and 0.078 (f) ng DNA per reaction.

| Locus | Mean | | | | | Median | | | | | Standard deviation | | | | | Number of heterozygotes | | | | |
| --- | --- | --- | --- | --- | --- | --- | --- | --- | --- | --- | --- | --- | --- | --- | --- | --- | --- | --- | --- | --- |
|  | b | c | d | e | f | b | c | d | e | f | b | c | d | e | f | b | c | d | e | f |
| OPPEN09 | 0.9 | 0.87 | 0.8 | 0.64 | 0.58 | 0.89 | 0.87 | 0.79 | 0.7 | 0.61 | 0.07 | 0.10 | 0.12 | 0.19 | 0.33 | 8 | 8 | 9 | 7 | 4 |
| OPPEN10 | 0.93 | 0.88 | 0.82 | 0.92 | 0.54 | 0.92 | 0.93 | 0.84 | 0.91 | 0.55 | 0.05 | 0.11 | 0.12 | 0.02 | 0.11 | 8 | 8 | 7 | 3 | 4 |
| OPPEN12 | 0.93 | 0.87 | 0.81 | 0.68 | 0.78 | 0.93 | 0.90 | 0.81 | 0.68 | 0.79 | 0.04 | 0.13 | 0.11 | 0.18 | 0.15 | 9 | 9 | 9 | 7 | 4 |
| OPPEN18 | 0.77 | 0.78 | 0.53 | 0.39 | 0.27 | 0.80 | 0.79 | 0.46 | 0.30 | 0.15 | 0.11 | 0.10 | 0.21 | 0.28 | 0.30 | 8 | 8 | 9 | 5 | 6 |
| OPPEN22 | 0.84 | 0.86 | 0.86 | 0.98 | 0.60 | 0.85 | 0.88 | 0.90 | 0.98 | 0.48 | 0.08 | 0.13 | 0.08 | - | 0.24 | 5 | 5 | 5 | 1 | 3 |
| OPPEN29 | 0.92 | 0.89 | 0.87 | 0.66 | 0.57 | 0.93 | 0.88 | 0.89 | 0.66 | 0.56 | 0.06 | 0.08 | 0.06 | 0.18 | 0.30 | 7 | 8 | 8 | 6 | 4 |
| OPPEN30 | 0.92 | 0.84 | 0.77 | 0.71 | 0.51 | 0.94 | 0.82 | 0.76 | 0.65 | 0.41 | 0.06 | 0.09 | 0.09 | 0.17 | 0.28 | 9 | 9 | 9 | 7 | 4 |
| OPPEN38 | 0.88 | 0.87 | 0.72 | 0.54 | 0.55 | 0.89 | 0.90 | 0.75 | 0.58 | 0.56 | 0.09 | 0.11 | 0.22 | 0.16 | 0.12 | 6 | 8 | 8 | 4 | 4 |
| OPPEN41 | 0.91 | 0.87 | 0.82 | 0.67 | 0.81 | 0.90 | 0.87 | 0.86 | 0.67 | 0.88 | 0.07 | 0.07 | 0.15 | 0.12 | 0.15 | 9 | 10 | 10 | 6 | 7 |
| OPTET024b | 0.95 | 0.86 | 0.76 | 0.55 | 0.80 | 0.95 | 0.86 | 0.73 | 0.36 | 0.80 | 0.03 | 0.07 | 0.18 | 0.36 | - | 4 | 4 | 4 | 3 | 1 |
| OPTET025d | 0.91 | 0.94 | 0.77 | 0.63 | 0.55 | 0.92 | 0.95 | 0.76 | 0.69 | 0.60 | 0.07 | 0.07 | 0.12 | 0.16 | 0.22 | 8 | 8 | 8 | 7 | 5 |
| OPTET029 | 0.92 | 0.88 | 0.83 | 0.73 | 0.57 | 0.91 | 0.87 | 0.86 | 0.78 | 0.57 | 0.02 | 0.06 | 0.14 | 0.13 | 0.27 | 7 | 7 | 7 | 4 | 4 |
| OPTET030 | 0.89 | 0.88 | 0.82 | 0.67 | 0.91 | 0.89 | 0.90 | 0.82 | 0.74 | 0.91 | 0.08 | 0.09 | 0.07 | 0.27 | 0.06 | 6 | 6 | 6 | 5 | 4 |
| OPTET063 | 0.93 | 0.82 | 0.88 | 0.68 | 0.45 | 0.95 | 0.83 | 0.91 | 0.72 | 0.49 | 0.06 | 0.09 | 0.14 | 0.29 | 0.10 | 11 | 11 | 11 | 9 | 4 |
| OPTET065 | 0.92 | 0.91 | 0.85 | 0.71 | 0.51 | 0.92 | 0.95 | 0.85 | 0.73 | 0.45 | 0.06 | 0.09 | 0.10 | 0.20 | 0.31 | 5 | 5 | 5 | 3 | 3 |
| OPTET082b | 0.99 | 0.94 | 0.78 | - | 0.82 | 0.99 | 0.94 | 0.81 | - | 0.82 | 0.00 | 0.04 | 0.08 | - | - | 2 | 3 | 3 | - | 1 |
| OPTET089b | 0.88 | 0.92 | 0.79 | 0.70 | 0.85 | 0.88 | 0.95 | 0.74 | 0.68 | 0.85 | 0.08 | 0.06 | 0.13 | 0.15 | 0.08 | 5 | 5 | 5 | 3 | 2 |
| OPTET094 | 0.95 | 0.89 | 0.76 | 0.78 | 0.83 | 0.95 | 0.88 | 0.76 | 0.89 | 0.81 | 0.04 | 0.06 | 0.14 | 0.22 | 0.08 | 7 | 7 | 7 | 7 | 4 |
| OPTET105 | 0.87 | 0.91 | 0.87 | 0.88 | 0.68 | 0.92 | 0.91 | 0.86 | 0.89 | 0.78 | 0.10 | 0.04 | 0.08 | 0.06 | 0.31 | 5 | 6 | 6 | 3 | 3 |
| OPTET108 | 0.90 | 0.84 | 0.50 | - | 0.64 | 0.90 | 0.84 | 0.50 | - | 0.64 | - | 0.08 | 0.07 | - | 0.09 | 1 | 2 | 2 | - | 2 |
| OPTET119 | 0.94 | 0.81 | 0.88 | 0.79 | 0.60 | 0.97 | 0.81 | 0.98 | 0.79 | 0.61 | 0.07 | 0.10 | 0.14 | 0.25 | 0.33 | 5 | 5 | 5 | 2 | 3 |
| OPTET127 | 0.92 | 0.90 | 0.97 | - | - | 0.92 | 0.90 | 0.97 | - | - | - | - | - | - | - | 1 | 1 | 1 | - | - |
| OPTET156 | 0.92 | 0.89 | 0.87 | 0.71 | 0.94 | 0.93 | 0.89 | 0.95 | 0.71 | 0.94 | 0.06 | 0.07 | 0.15 | 0.18 | - | 5 | 5 | 5 | 2 | 1 |
| OPTET278 | 0.89 | 0.92 | 0.91 | 0.65 | 0.79 | 0.89 | 0.92 | 0.92 | 0.65 | 0.97 | 0.09 | 0.04 | 0.04 | 0.10 | 0.32 | 4 | 4 | 4 | 3 | 3 |
| OPTRI0245b | 0.90 | 0.90 | 0.78 | 0.63 | 0.65 | 0.92 | 0.90 | 0.85 | 0.60 | 0.60 | 0.08 | 0.07 | 0.18 | 0.07 | 0.17 | 11 | 11 | 10 | 6 | 6 |
| OPTRI1592 | 0.94 | 0.81 | 0.81 | 0.67 | 0.48 | 0.98 | 0.80 | 0.83 | 0.71 | 0.43 | 0.05 | 0.09 | 0.16 | 0.21 | 0.17 | 7 | 7 | 7 | 6 | 3 |
| OPTRI1870 | 0.87 | 0.85 | 0.80 | 0.68 | 0.64 | 0.87 | 0.89 | 0.81 | 0.70 | 0.61 | 0.04 | 0.14 | 0.09 | 0.14 | 0.14 | 8 | 8 | 8 | 6 | 4 |
| mean | 0.91 | 0.87 | 0.80 | 0.69 | 0.65 | 0.92 | 0.88 | 0.81 | 0.70 | 0.65 | 0.06 | 0.08 | 0.12 | 0.18 | 0.20 | 6.33 | 6.59 | 6.59 | 4.79 | 3.58 |

**Supplementary Table S11**. Heterozygote balance (Hb´) for samples with 1.25 (b), 0.625 (c), 0.313 (d), 0.156 (e) and 0.078 (f) ng DNA per reaction.

| MTP | Marker | n-k/*n-2 | | | n-2k/*n-3 | | *n-4 | | n+k | |
| --- | --- | --- | --- | --- | --- | --- | --- | --- | --- | --- |
|  |  | $\bar{x}$ [%] | SD [%] | $\bar{x}$+3SD [%] | $\bar{x}$ [%] | SD [%] | $\bar{x}$ [%] | SD [%] | $\bar{x}$ [%] | SD [%] |
| 1 | OPPEN12 | 2.74 | 1.26 | 6.53 | - | - | - | - | 1.76 | 1.56 |
| 1 | OPPEN18 | 2.16 | 0.98 | 5.11 | 0.75 | 0.36 | - | - | 1.24 | 1.05 |
| 1 | OPPEN22* | 5.88 | 1.88 | 11.53 | 3.28 | 0.87 | 2.35 | 0.30 | - | - |
| 1 | OPTET029 | 3.01 | 0.76 | 5.29 | 0.80 | 0.12 | - | - | 0.53 | 0.29 |
| 1 | OPTET030 | 4.08 | 2.05 | 10.22 | 0.28 | - | - | - | 0.62 | 0.51 |
| 1 | OPTET063 | 4.74 | 2.29 | 11.60 | 0.71 | 0.75 | - | - | 0.51 | 0.31 |
| 1 | OPTET156 | 1.6 | 0.59 | 3.37 | 1.68 | 0.28 | - | - | - | - |
| 1 | OPTET278 | 1.36 | 0.30 | 2.28 | 0.57 | 0.26 | - | - | 1.23 | 0.97 |
| 1 | OPTRI1592 | 4.23 | 2.11 | 10.55 | 0.96 | 0.63 | - | - | 1.64 | 2.46 |
|  |  |  |  |  |  |  |  |  |  |  |
| 2 | OPPEN29 | 3.53 | 0.59 | 5.29 | 0.95 | 0.52 | - | - | - | - |
| 2 | OPPEN30 | 2.05 | 0.77 | 4.36 | 0.43 | 0.13 | - | - | - | - |
| 2 | OPTET024b | 3.90 | 2.25 | 10.64 | - | - | - | - | - | - |
| 2 | OPTET025d | 7.01 | 2.86 | 15.59 | 1.04 | 0.53 | - | - | 0.93 | 0.81 |
| 2 | OPTET089b | 2.35 | 1.70 | 7.45 | - | - | - | - | - | - |
| 2 | OPTET094 | 1.78 | 0.54 | 3.39 | 0.66 | 0.09 | - | - | 0.94 | 0.27 |
| 2 | OPTET105 | 4.42 | 3.50 | 14.94 | 0.90 | 0.40 | - | - | 0.75 | 0.21 |
| 2 | OPTRI0245b | 4.83 | 1.94 | 10.65 | - | - | - | - | 4.79 | 2.72 |
| 2 | OPTRI1870 | 8.65 | 1.51 | 13.17 | 1.14 | 0.38 | - | - | 0.76 | 0.24 |
|  |  |  |  |  |  |  |  |  |  |  |
| 3 | OPPEN09* | 9.07 | 0.52 | 10.62 | 5.68 | 0.38 | 3.51 | 0.34 | - | - |
| 3 | OPPEN10 | 1.92 | 0.68 | 3.94 | 0.99 | 0.22 | - | - | 2.05 | - |
| 3 | OPPEN38 | 3.56 | 0.64 | 5.49 | 0.41 | 0.13 | - | - | 1.43 | 1.22 |
| 3 | OPPEN41 | 2.64 | 0.51 | 4.17 | 0.76 | 0.32 | - | - | 1.58 | 1.15 |
| 3 | OPTET065 | 3.59 | 2.16 | 10.06 | 0.56 | 0.11 | - | - | 4.15 | 4.73 |
| 3 | OPTET082b | 2.59 | 1.19 | 6.16 | 1.33 | 0.64 | - | - | 1.21 | 0.67 |
| 3 | OPTET108 | 2.74 | 1.84 | 8.26 | 0.62 | 0.21 | - | - | 0.75 | 0.38 |
| 3 | OPTET119 | 4.94 | 2.98 | 13.87 | 2.18 | 2.79 | - | - | 0.94 | 0.36 |
| 3 | OPTET127 | 2.34 | 0.52 | 3.91 | 0.55 | 0.16 | - | - | - | - |

**Supplementary Table S12.** Stutter profile of forward, backward and double backward stutters described by mean ($\bar{x}$) and standard deviation (SD). n – true number of repeats, k – one repeat unit with length 3, 4 or 5 bp depending on length of core motif; *in case of OPPEN22 and OPPEN09 marker was stutter ratio calculated for n – 2/3/4 bp.

| MTP | Locus | Core motif | Chr.^a^ | Localization | N_ind_^b^ | N_al_^c^ | H_Obs_^d^ | Accession number |
| --- | --- | --- | --- | --- | --- | --- | --- | --- |
| 1 | OPPEN12 | AAGAA | chr03 | 141,216,836 | 187 | 3 | 0.107 | MW364129 - MW364131 |
| 1 | OPPEN18 | CAAAA | chr04 | 41,376,123 | 187 | 4 | 0.112 | MW364132 - MW364135 |
| 1 | OPPEN22 | TTTGT | chr05 | 26,433,573 | 186 | 4 | 0.048 | MW364136 - MW364141 |
| 1 | OPTET029 | AATG | chr07 | 176,211,490 | 187 | 5 | 0.043 | MW364180 - MW364184 |
| 1 | OPTET030 | TCTA | chr07 | 214,108,214 | 187 | 4 | 0.048 | MW364185 - MW364188 |
| 1 | OPTET063 | CTTT | chr02 | 41,934,802 | 187 | 5 | 0.102 | MW364189 - MW364193 |
| 1 | OPTET156 | AAAT | chr01 | 214,502,085 | 185 | 3 | 0.070 | MW364242 - MW364244 |
| 1 | OPTET278 | TTCA | chr09 | 70,970,016 | 187 | 4 | 0.032 | MW364245 - MW364248 |
| 1 | OPTRI1592 | CTT | chr08 | 164,946,366 | 186 | 9 | 0.070 | MW364254 - MW364262 |
| 2 | OPPEN29 | GGATA | chr07 | 250,763,653 | 187 | 6 | 0.091 | MW364142 - MW364146 |
| 2 | OPPEN30 | AACA | chr08 | 28,121,974 | 187 | 7 | 0.118 | MW364147 - MW364153 |
| 2 | OPTET024b | AGTG | chr05 | 209,254,034 | 187 | 8 | 0.059 | MW364164 - MW364171 |
| 2 | OPTET025d | TGTC | chr06 | 11,597,476 | 187 | 8 | 0.128 | MW364172 - MW364179 |
| 2 | OPTET089b | AAGA | chr05 | 161,605,005 | 187 | 6 | 0.048 | MW364201 - MW364206 |
| 2 | OPTET094 | GATG | chr06 | 101,211,151 | 187 | 4 | 0.091 | MW364207 - MW364210 |
| 2 | OPTET105 | TATC | chr07 | 31,962,127 | 187 | 14 | 0.059 | MW364211 - MW364223 |
| 2 | OPTRI0245b | AGA | chr01 | 243,530,869 | 187 | 5 | 0.128 | MW364249 - MW364253 |
| 2 | OPTRI1870 | CTT | chr10 | 91,518,018 | 187 | 8 | 0.091 | MW364263 - MW364269 |
| 3 | OPPEN09 | GTTTT | chr02 | 153,528,081 | 182 | 5 | 0.082 | MW364117 - MW364121 |
| 3 | OPPEN10 | TTGTT | chr03 | 31,542,048 | 182 | 7 | 0.104 | MW364122 - MW364128 |
| 3 | OPPEN38 | TTTGT | chr09 | 182,103,434 | 179 | 4 | 0.084 | MW364154 - MW364157 |
| 3 | OPPEN41 | AAACA | chr11 | 16,836,283 | 182 | 8 | 0.110 | MW364158 - MW364163 |
| 3 | OPTET065 | GGAT | chr02 | 93,603,468 | 182 | 4 | 0.033 | MW364194 - MW364196 |
| 3 | OPTET082b | ATAA | chr04 | 156,473,889 | 169 | 5 | 0.047 | MW364197 - MW364200 |
| 3 | OPTET108 | CTTT | chr07 | 80,706,751 | 182 | 8 | 0.027 | MW364224 - MW364231 |
| 3 | OPTET119 | AGAC | chr09 | 7,947,287 | 182 | 7 | 0.088 | MW364232 - MW364238 |
| 3 | OPTET127 | TCAT | chr10 | 6,887,675 | 182 | 3 | 0.022 | MW364239 - MW364241 |
| mean | - | - | - | - | 185 | 5.85 | 0.076 | - |
| median | - | - | - | - | 187 | 5 | 0.082 | - |

**Supplementary Table S13.** Summary overview with information about STR markers. ^a^chromosome, ^b^number of individuals, ^c^number of alleles, ^d^observed heterozygosity.


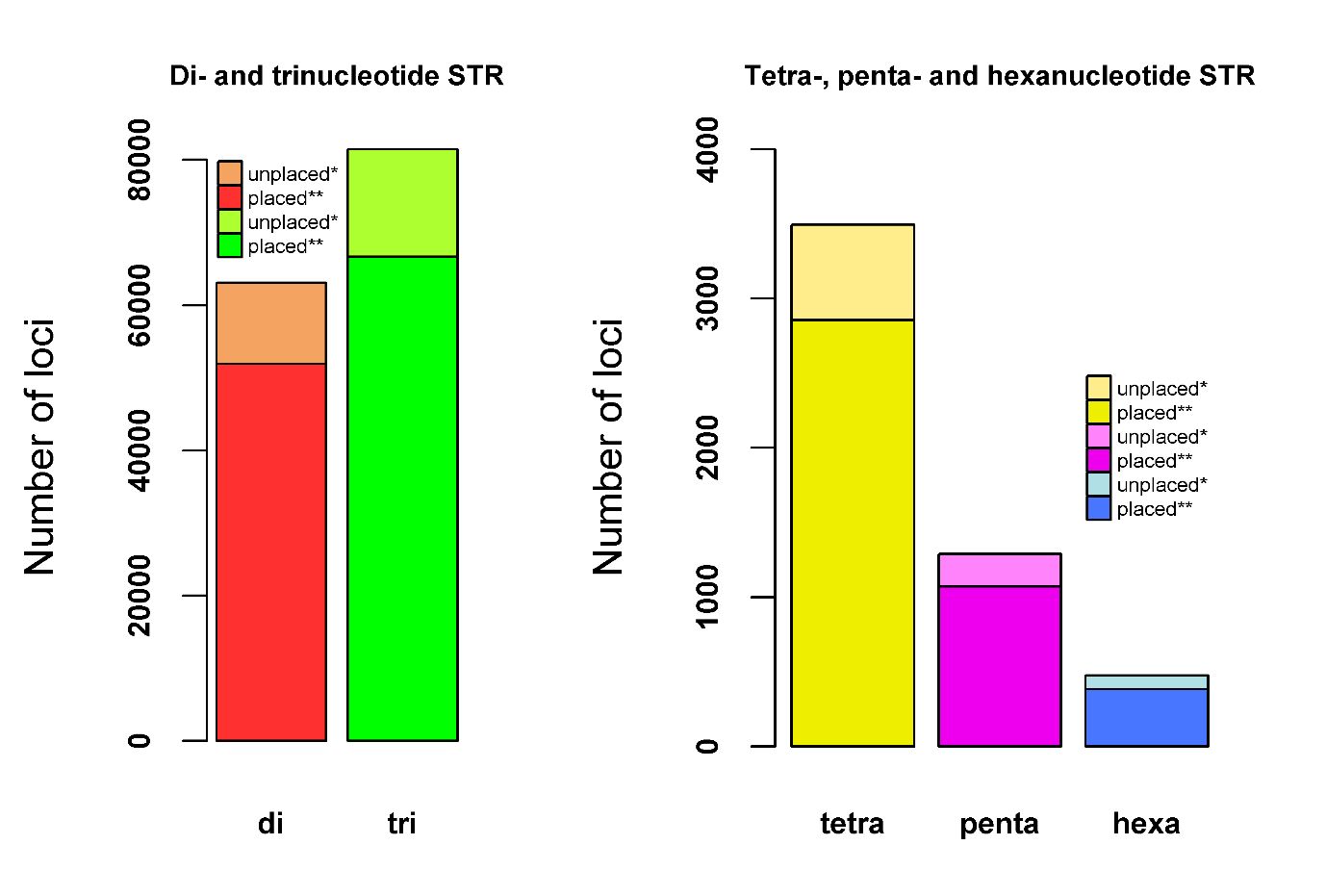


**Supplementary Figure S1.** Total number of STR loci in the poppy genome according to motif length. *STR with unknown position on chromosome, **STR with known position on chromosome. Please notice different scale on y axes. Created in R v4.0.3 (https://cran.r-project.org/)^1^.


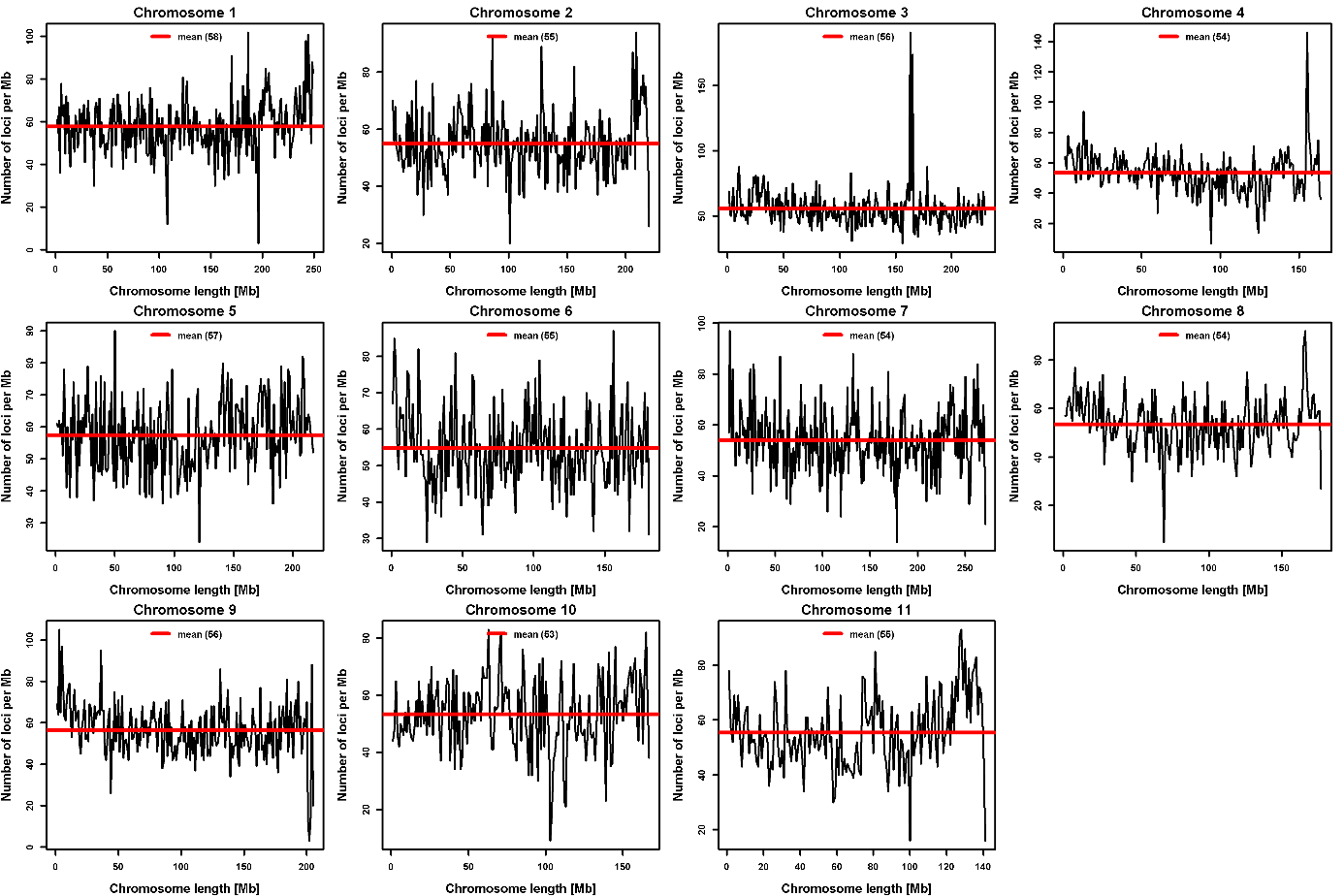


**Supplementary Figure S2.** STR density as the number of loci per megabase per chromosome. Created in R v4.0.3 (https://cran.r-project.org/)^1^.


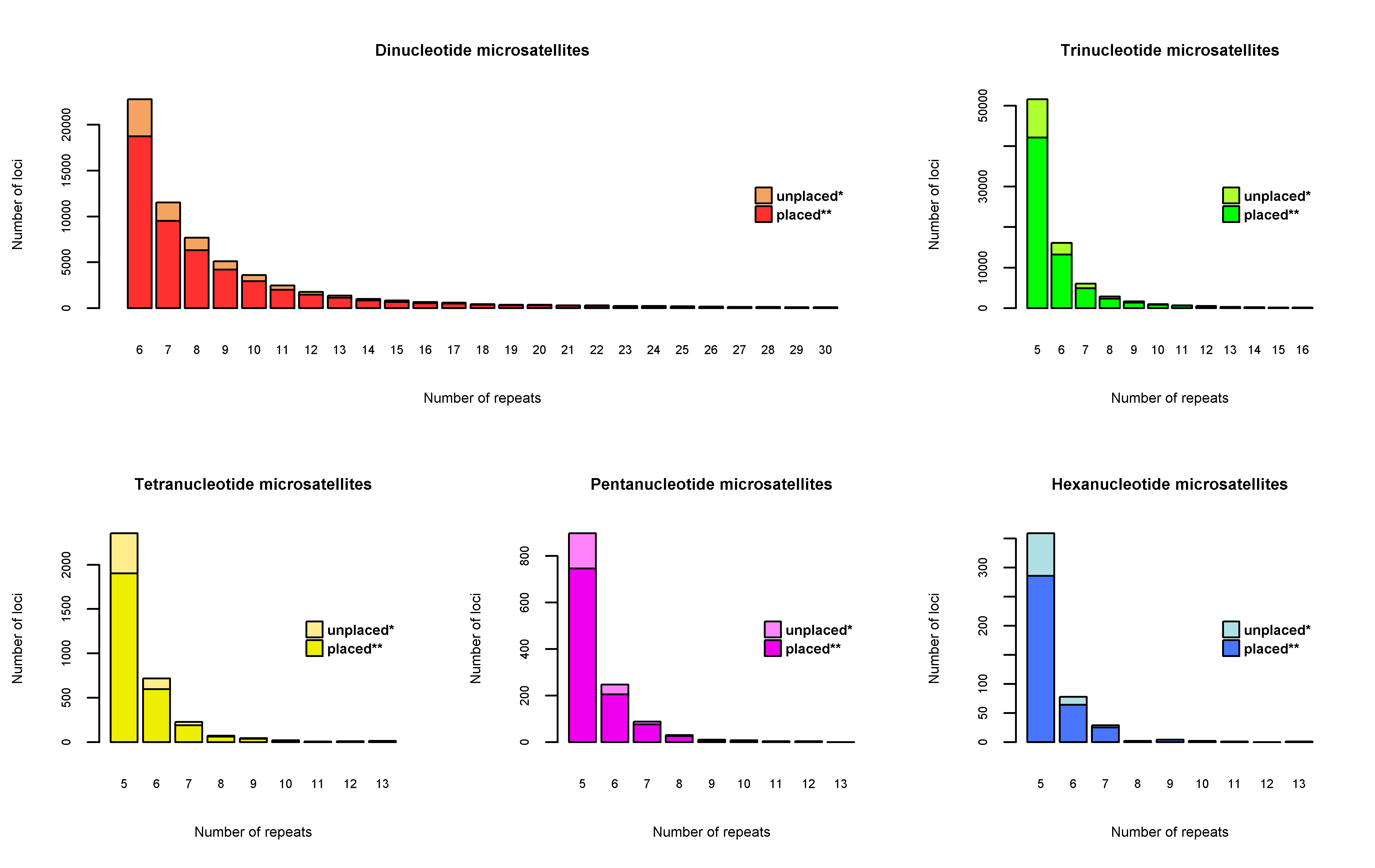


**Supplementary Figure S3.** Number of STRs in the poppy genome sorted according to the length of motifs and number of repeats. *STR with unknown position on chromosome, **STR with known position on chromosome. Please notice different scales on y axes. Created in R v4.0.3 (https://cran.r-project.org/)^1^.


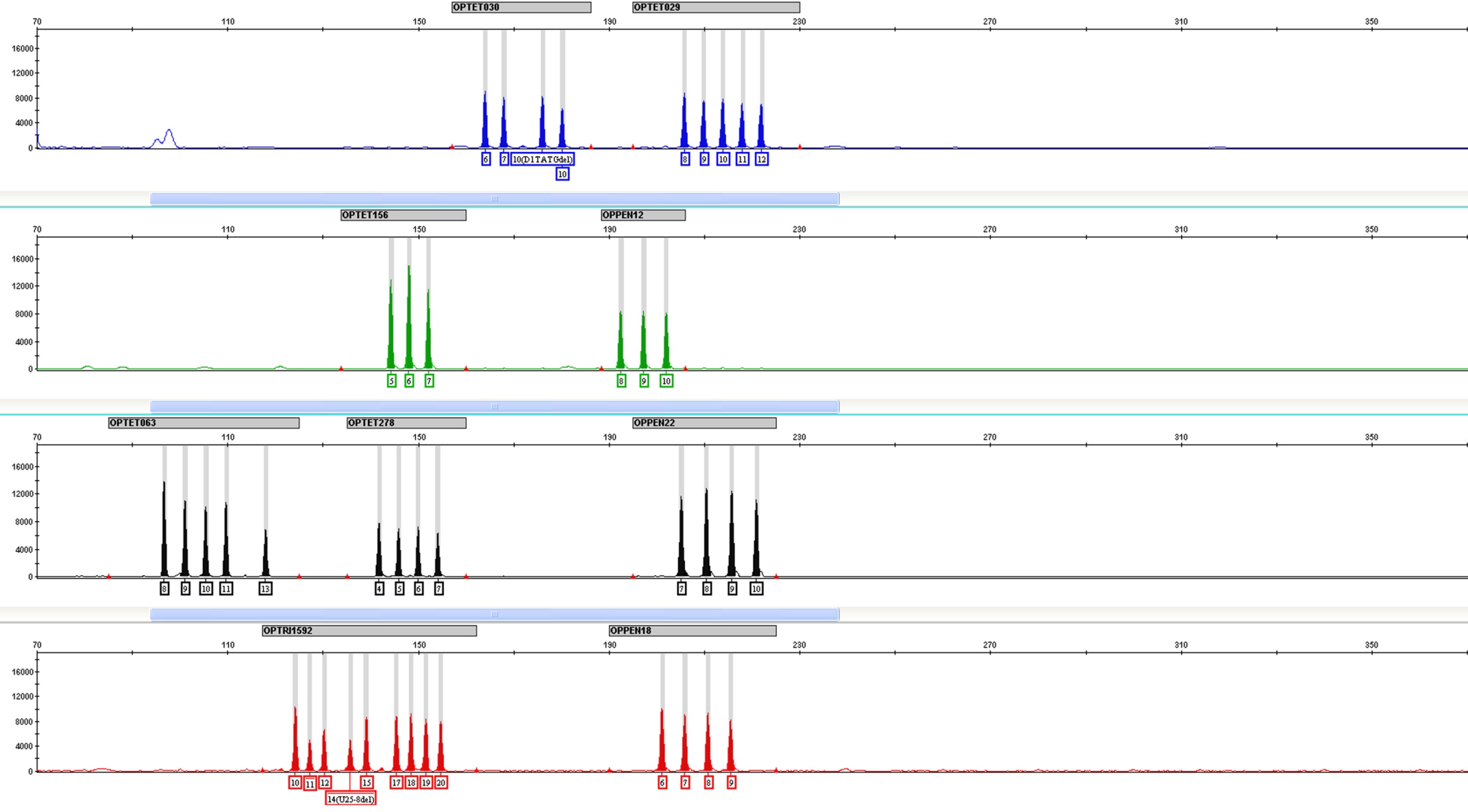


**Supplementary Figure S4.** MTP1 allelic ladder. Screenshot of GeneMapper v5.1 (Thermo Fisher Scientific) result.


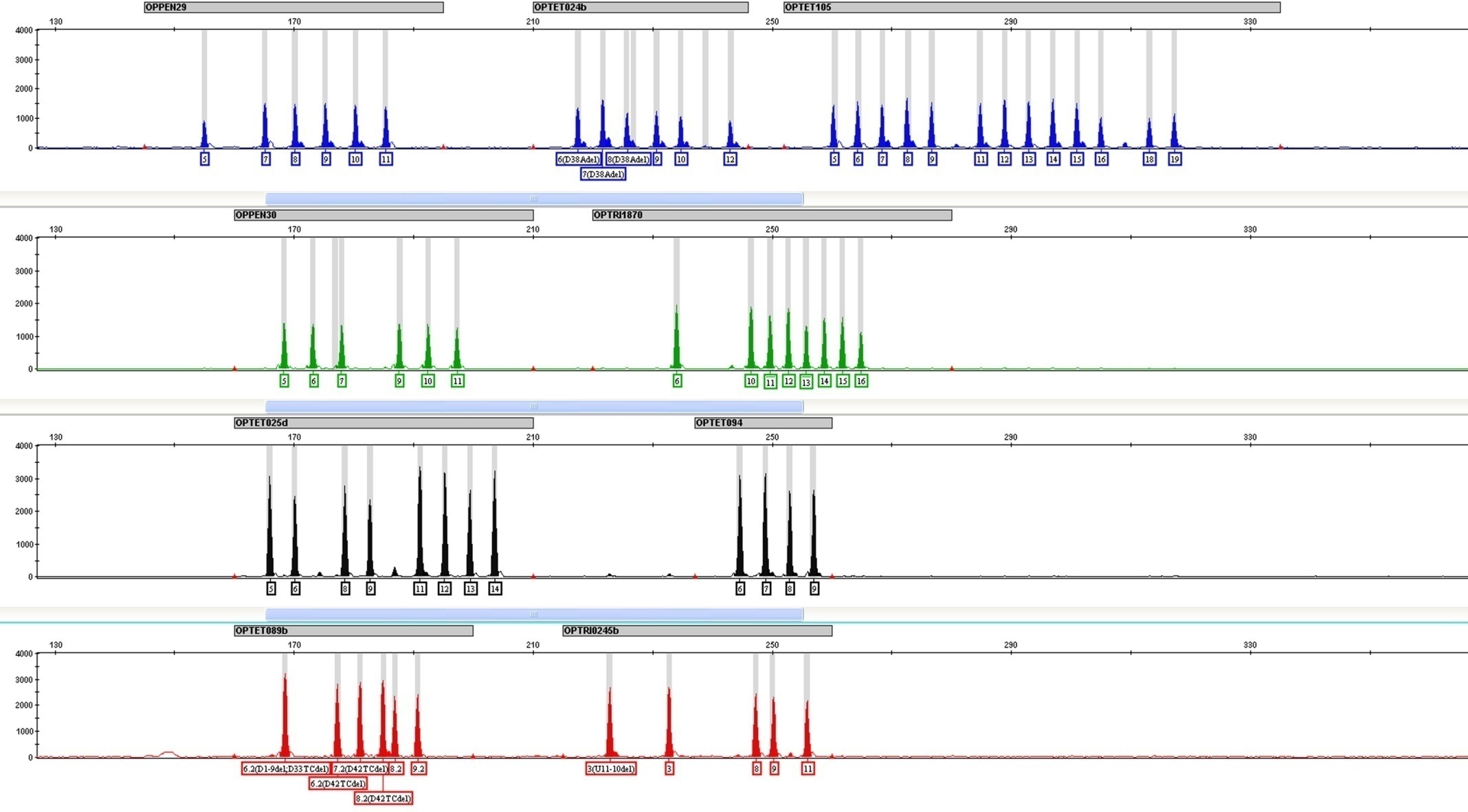


**Supplementary Figure S5.** MTP2 allelic ladder. Screenshot of GeneMapper v4.1 (Thermo Fisher Scientific) result.


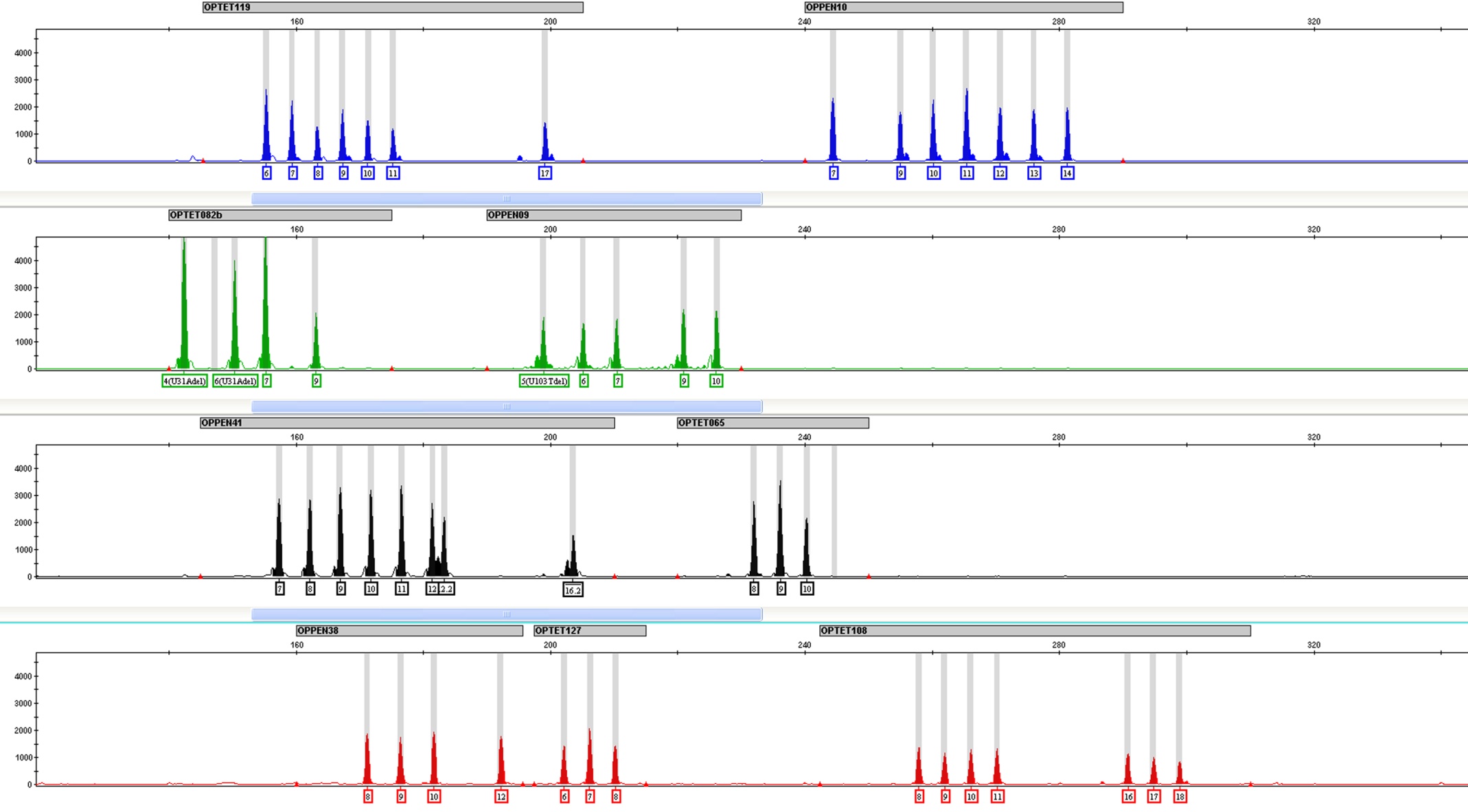


**Supplementary Figure S6.** MTP3 allelic ladder. Screenshot of GeneMapper v4.1 (Thermo Fisher Scientific) result.


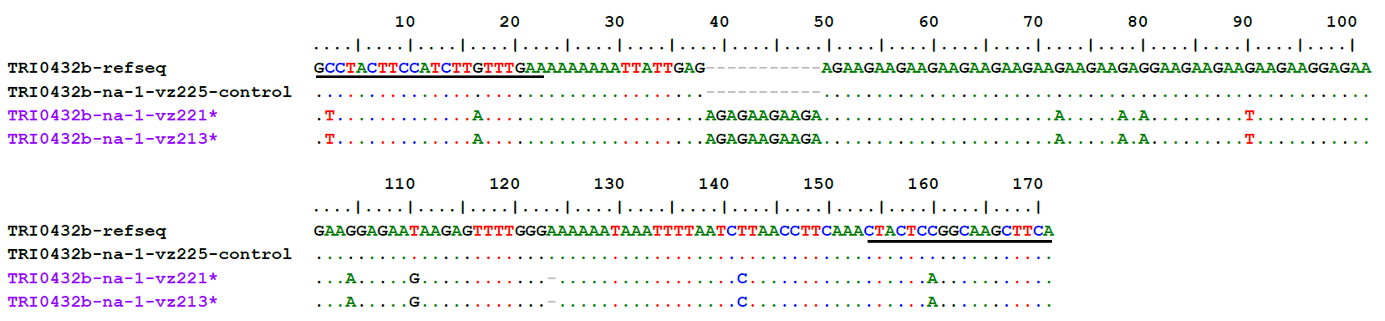


**Supplementary Figure S7.** Locus OPTRI0432b excluded from the profiling system due to null allele occurrence. Position of the original primers is underlined. *Null allele. Screenshot of BioEdit v7.0.9.0^2^ result.


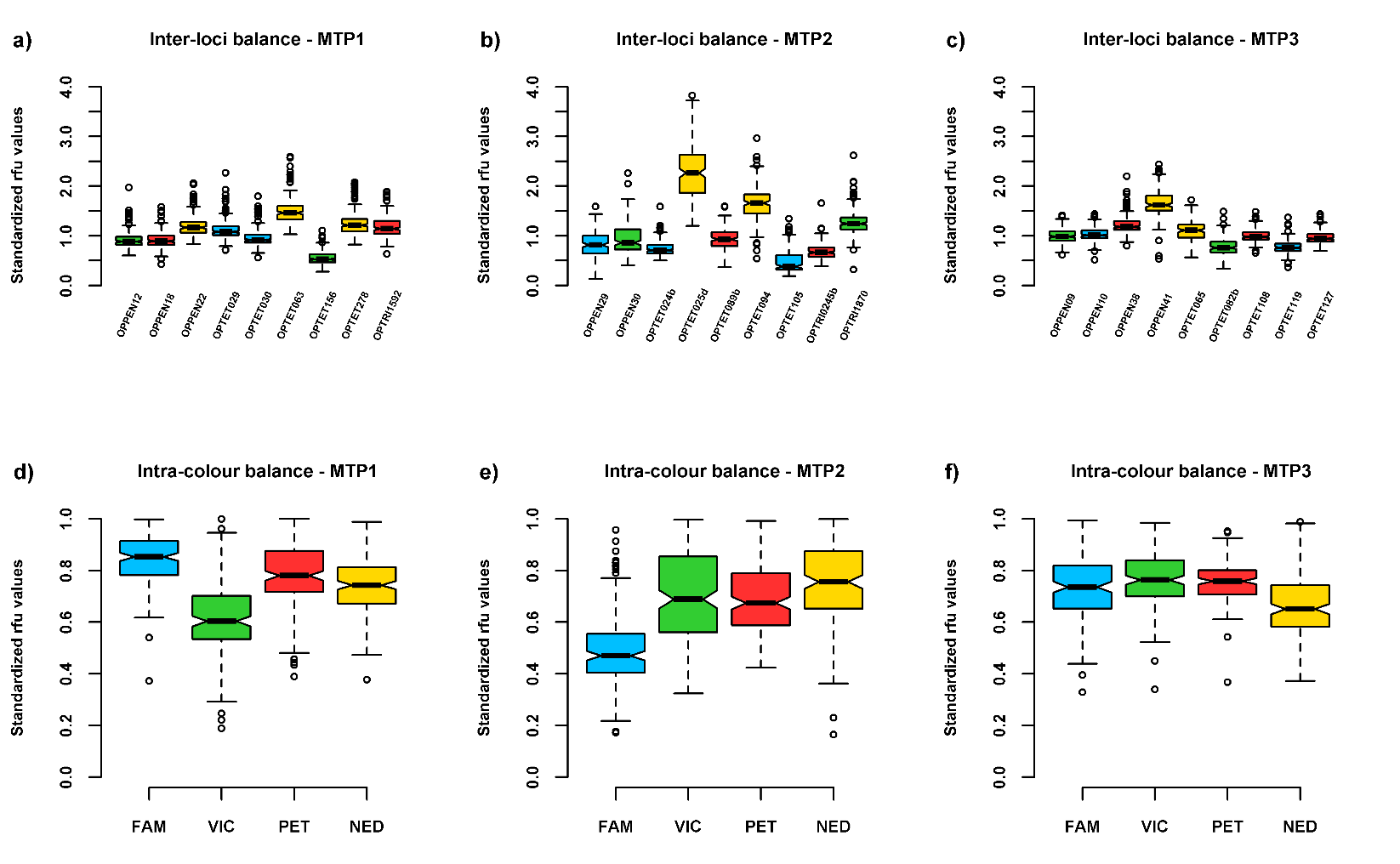


**Supplementary Figure S8.** Interloci balance (upper row) and intracolour balance (bottom row). Created in R v4.0.3 (https://cran.r-project.org/)^1^.


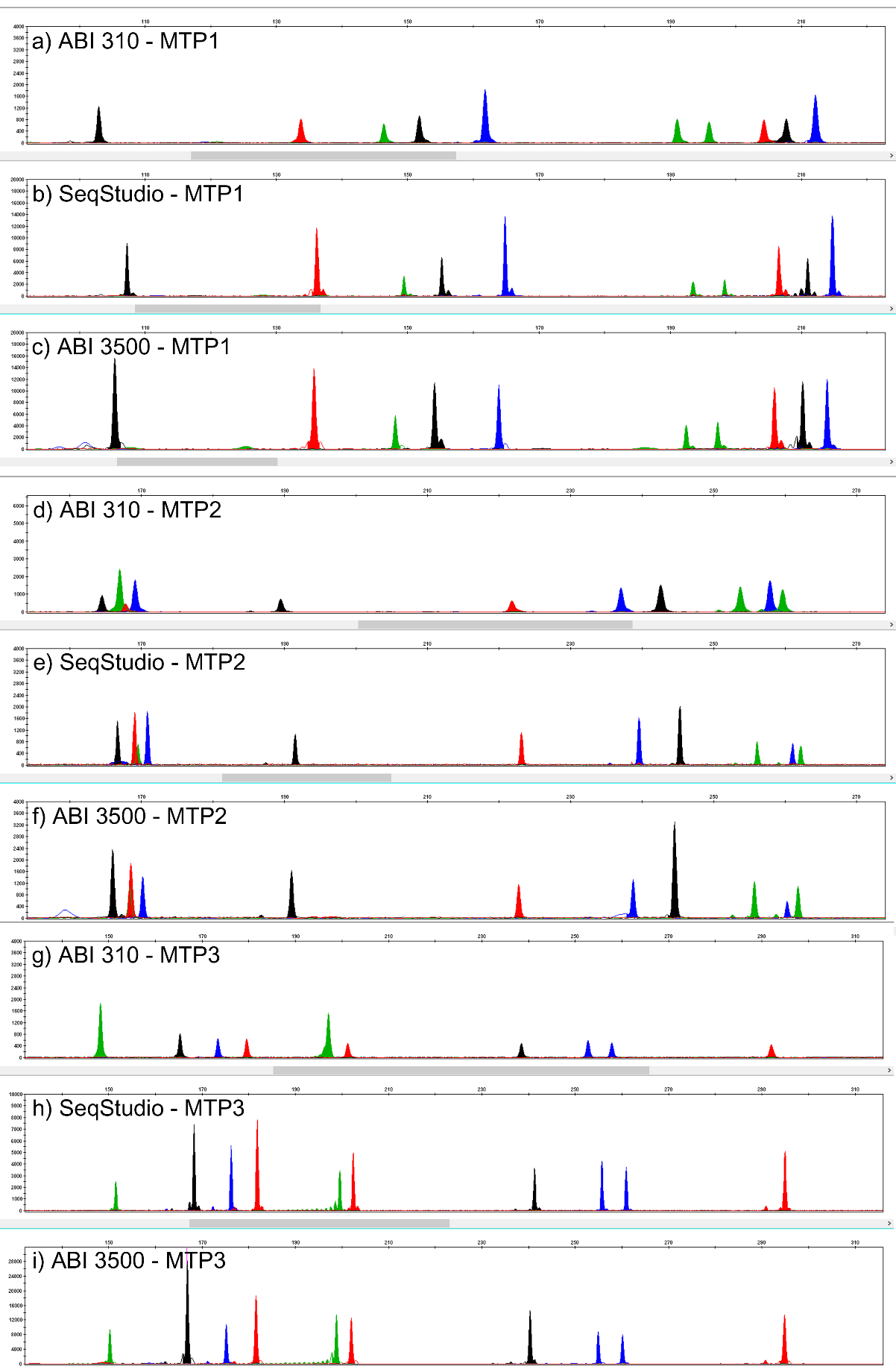


**Supplementary Figure S9.** Comparison of spectral calibration between ABI 310, SeqStudio and ABI 3500. Screenshot of GeneMapper v5.1 (Thermo Fisher Scientific) result.


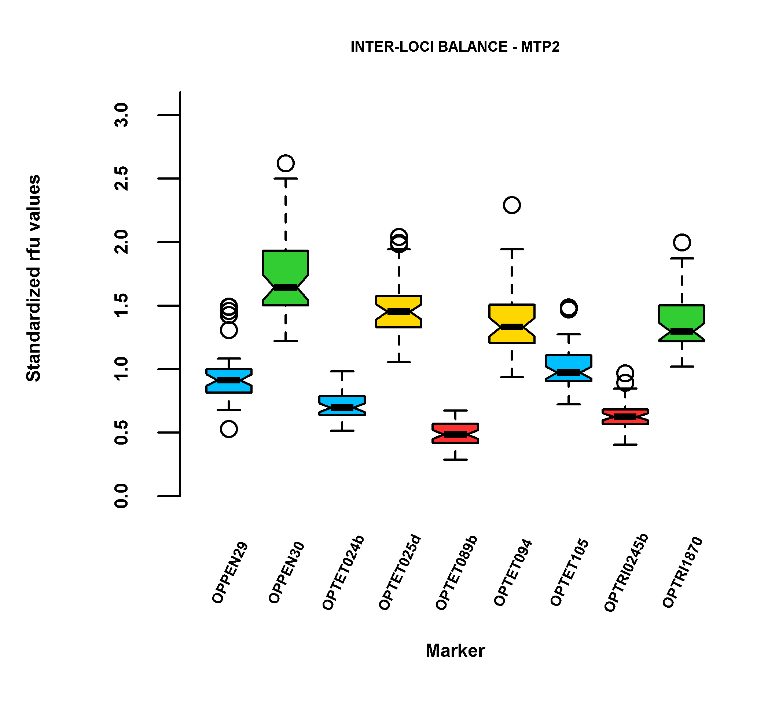


**Supplementary Figure S10.** IELB result of modified MTP2 formula for the ABI 3500. Created in R v4.0.3 (https://cran.r-project.org/)^1^.


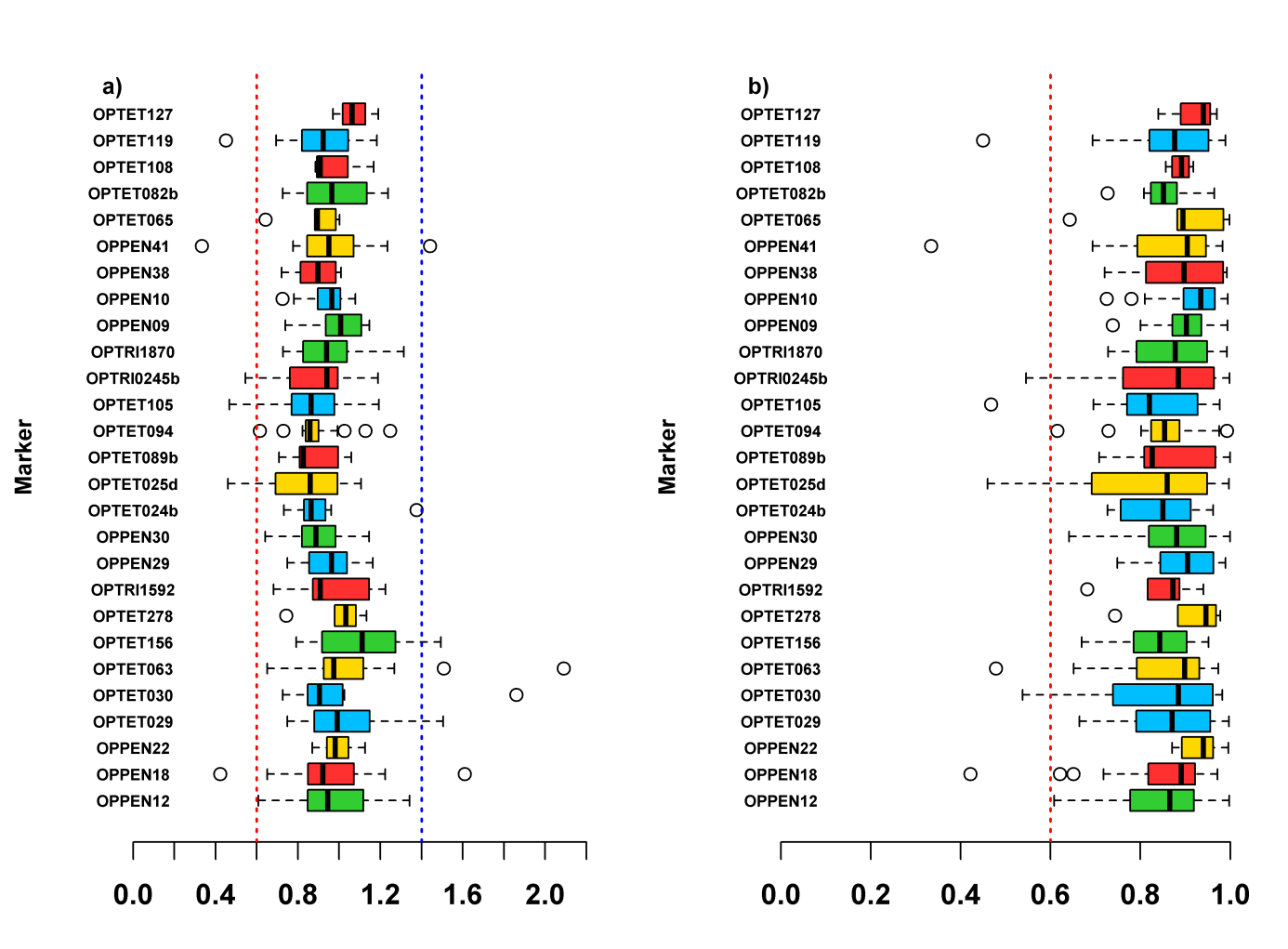


**Supplementary Figure S11.** Heterozygote balance according to the Hb (a) and Hb´ (b) methods. Dotted lines represent typically used lower (red) and upper (blue) boundaries. Outliers excluded from summary statistics are also depicted. Created in R v4.0.3 (https://cran.r-project.org/)^1^.


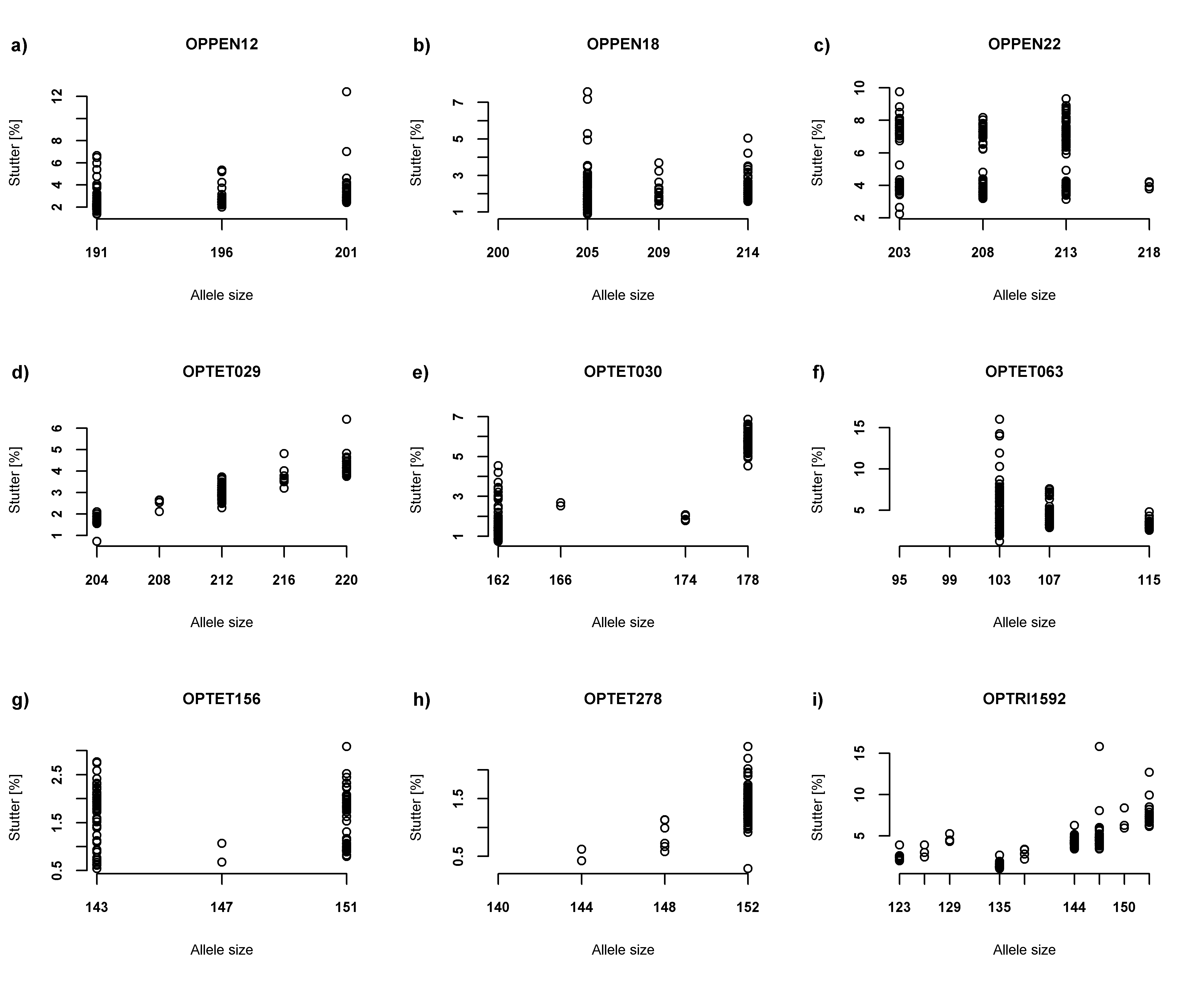


**Supplementary Figure S12.** Allele specific stutters of MTP1 markers. Created in R v4.0.3 (https://cran.r-project.org/)^1^.


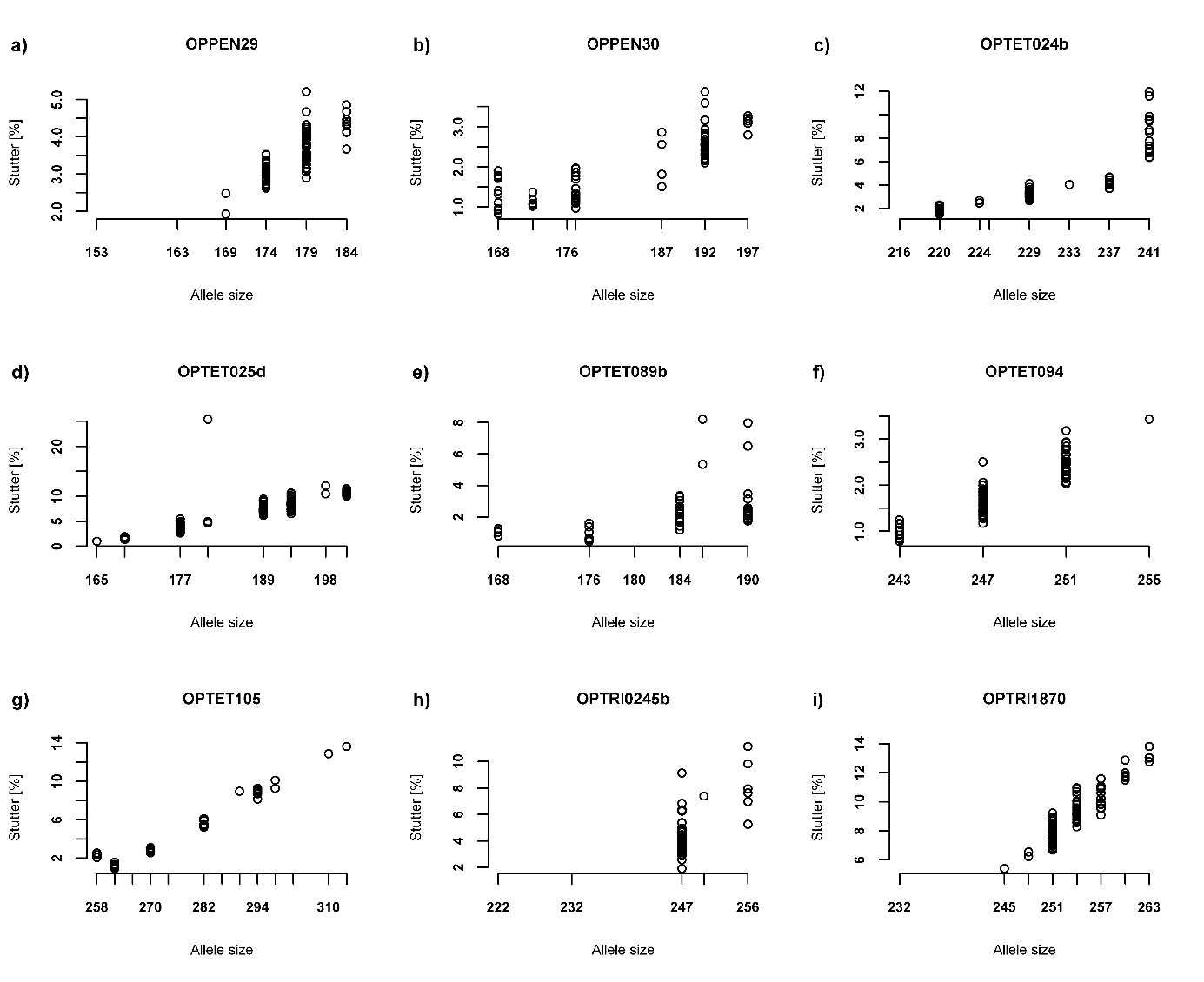


**Supplementary Figure S13.** Allele specific stutters of MTP2 markers. Created in R v4.0.3 (https://cran.r-project.org/)^1^.


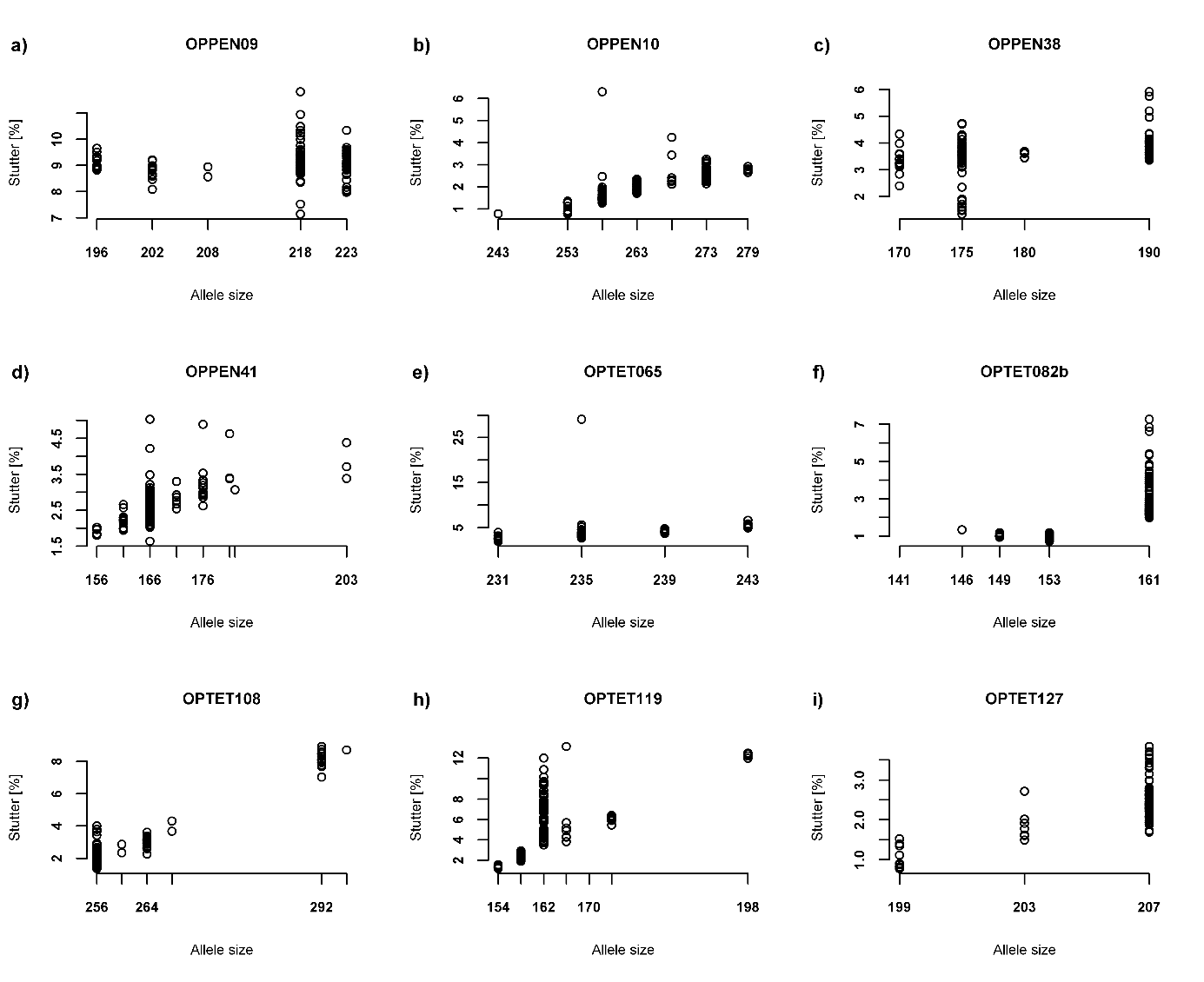


**Supplementary Figure S14.** Allele specific stutters of MTP3 markers. Created in R v4.0.3 (https://cran.r-project.org/)^1^.

**References**

1. R Core Team. R: *A language and environment for statistical computing*. (R Foundation for Statistical Computing, 2020).

2. Hall, T., A. BioEdit: a user-friendly biological sequence alignment editor and analysis program for Windows 95/98/NT. in Nucleic acids symposium series vol. 41 95–98 (1999).
